# Cerebrotypes in Cephalopods: Brain Diversity and Its Correlation With Species Habits, Life History, and Physiological Adaptations

**DOI:** 10.3389/fnana.2020.565109

**Published:** 2021-02-02

**Authors:** Giovanna Ponte, Morag Taite, Luciana Borrelli, Andrea Tarallo, A. Louise Allcock, Graziano Fiorito

**Affiliations:** ^1^Department of Biology and Evolution of Marine Organisms, Stazione Zoologica Anton Dohrn, Naples, Italy; ^2^Department of Zoology, Ryan Institute, National University of Ireland Galway, Galway, Ireland; ^3^Department of Research Infrastructures for Marine Biological Resources (RIMAR), Stazione Zoologica Anton Dohrn, Naples, Italy

**Keywords:** neuroecology, cephalopods, brain diversity, adaptation, evolution

## Abstract

Here we analyze existing quantitative data available for cephalopod brains based on classical contributions by J.Z. Young and colleagues, to cite some. We relate the relative brain size of selected regions (area and/or lobe), with behavior, life history, ecology and distribution of several cephalopod species here considered. After hierarchical clustering we identify and describe ten clusters grouping 52 cephalopod species. This allows us to describe cerebrotypes, i.e., differences of brain composition in different species, as a sign of their adaptation to specific niches and/or clades in cephalopod molluscs for the first time. Similarity reflecting niche type has been found in vertebrates, and it is reasonable to assume that it could also occur in Cephalopoda. We also attempted a phylogenetic PCA using data by Lindgren et al. (2012) as input tree. However, due to the limited overlap in species considered, the final analysis was carried out on <30 species, thus reducing the impact of this approach. Nevertheless, our analysis suggests that the phylogenetic signal alone cannot be a justification for the grouping of species, although biased by the limited set of data available to us. Based on these preliminary findings, we can only hypothesize that brains evolved in cephalopods on the basis of different factors including phylogeny, possible development, and the third factor, i.e., life-style adaptations. Our results support the working hypothesis that the taxon evolved different sensorial and computational strategies to cope with the various environments (niches) occupied in the oceans. This study is novel for invertebrates, to the best of our knowledge.

## Introduction

Cephalopoda is the most charismatic class of the phylum Mollusca. The richness of their behavioral repertoire inspired many aspects of human life including contemporary art (as provided by camouflage and body patterns, see Nakajima, 2018) and robotics (e.g., Cianchetti et al., 2012; Xie et al., 2020; as inspired by the study of soft, flexible, and muscular body). In recent years, increased interest for their commercial value inspired gastronomy (Mouritsen and Styrbæk, 2018; see also: Sörensen and Mouritsen, 2019 and Cephs & Chefs: https://www.cephsandchefs.com/). In addition, social media provided access to specialized information and growing interest in interdisciplinary academic fields, and images and videos where cephalopods represent a great example (Nakajima et al., 2018; McClain, 2019). Together with fishes, images of cephalopods have been “liked” more than other organisms (including sharks) on social media platforms (McClain, 2019).

Cephalopods are an ancient taxon that diverged from a monoplacophoran ancestor about 500 million years ago, during the late Cambrian (see also Allcock et al., 2015). The early Devonian saw the rise of the ammonites and nautiloids (Kröger et al., 2011), both still with chambered shells. The greatest structural innovation, the internalization of the cephalopod shell, likely occurred in the Permian or Carboniferous (Smith and Caron, 2010; Kröger et al., 2011; Tanner et al., 2017; Klug et al., 2019), exposing the mantle for the first time and providing a possible significant boost to their evolution, including cognitive abilities (Packard, 1972; Amodio et al., 2019a,b).

Cephalopods evolved several innovations, the most intriguing perhaps being their capability of exhibiting rapid and neurally-controlled changes in their body patterning (Packard and Hochberg, 1977; Packard, 1988; Messenger, 2001; Borrelli et al., 2006), and a large and complex nervous system (review in: Nixon and Young, 2003; Shigeno et al., 2018).

During their evolution the brain of cephalopods increased its complexity reaching the maximum agglomeration of the neural masses, as exemplified by comparing the outline of the “central nervous system” of *Nautilus* and that of *Octopus vulgaris* (Young, 1965, 1971). This resulted after the addition or loss of ganglia (molluscan origin and plan) that brought about the change in position and relative volume, achieving features considered to be unusual to molluscan, and invertebrate or even vertebrate, standards, but allowing significant functional analogies with vertebrates (see: Bullock, 1965; Young, 1971, 1974, 1976, 1977b, 1979; Messenger, 1979; Budelmann B., 1995; Nixon and Young, 2003; Shigeno et al., 2018; see also Supplementary Information).

These animals have been the preeminent “model” for cephalopod developmental (Naef, 1928), neurophysiological (e.g., Keynes, 1989; Pozzo-Miller et al., 1998; Brown and Piscopo, 2013) and behavioral studies (review in e.g., Huffard, 2013), including an early systematic attempt to develop a model of the brain (Young, 1964; review in Marini et al., 2017). Furthermore, cephalopods (and maybe octopuses especially) exhibit advanced cognitive faculties paralleling mammalian capabilities (Edelman and Seth, 2009; Amodio et al., 2019b). Cephalopods also provide a very interesting case study for the evolution of novelties/innovations in Metazoa (for review see for example: Shigeno et al., 2018; Zarrella et al., 2019; Albertin and Simakov, 2020). These innovations originated through an increase in genome complexity linked to polyploidy, differential arrangements of key genes, exceptional RNA editing capacities, and expansion of transposable elements, to cite some (e.g., Packard and Albergoni, 1970; De Marianis et al., 1979; Lee et al., 2003; Liscovitch-Brauer et al., 2017; Zarrella et al., 2019; Albertin and Simakov, 2020).

Cephalopods have the largest and most complex invertebrate nervous system. During evolution, the “brain” was assembled through the fusion of a number of molluscan ganglia to form lobes connected to the periphery by many nerve trunks regulating the arms, viscera and other parts of the animal's body. Although their nervous system is confined to the basic molluscan form comprising a set of (five to) six pairs of ganglia, it has a complexity akin to that of lower vertebrates (Bullock, 1965; Budelmann B. U., 1995), but with important functional analogies when compared with higher vertebrates (Shigeno et al., 2018). In addition, cephalopods are known for a brain-to-body weight ratio that exceeds that of fishes and reptiles (Packard, 1972). These features correlate with the sophisticated sensory equipment and complex behavior that cephalopods display (for review see for example: Budelmann et al., 1997; Williamson and Chrachri, 2004; Hanlon and Messenger, 2018).

The central nervous system of cephalopods is characterized by a high level of organization and is therefore considered to be a “proper” brain, which is unusual by molluscan, invertebrate, and even vertebrate standards (Young, 1967; Budelmann B. U., 1995; Hochner et al., 2006; Shigeno et al., 2018) for: (i) the highest degree of centralization compared with any other mollusc or invertebrate (insects excluded), achieved by the shortening of the connectives; (ii) the presence of very small neurons (3–5 micron of nuclear size) acting as local interneurons (Young, 1971, 1991); (iii) the reported absence of somatotopy (except for the chromatophore lobes) contrary to what appears to be the case for the insect or vertebrate brain (Plän, 1987; Zullo, 2004; Zullo et al., 2009); (iv) a blood-brain barrier, an exception for molluscs (Abbott and Pichon, 1987); (v) compound field potentials, similar to those of vertebrate brains (e.g., Bullock and Budelmann, 1991; Williamson and Chrachri, 2004; for review see Brown and Piscopo, 2013); (vi) an elevated efferent innervation of the receptors (e.g., the retina, the equilibrium receptor organs); (vii) peripheral first order afferent neurons (see: Young, 1971, 1991; Brown and Piscopo, 2013); (viii) a large variety of putative transmitters (review in Messenger, 1996).

The greatest centralization among cephalopods is found in the octopodiforms. It is achieved by the shortening of the connectives between the superior buccal and brachial lobes (Nixon and Young, 2003). In contrast, *Nautilus* has the simplest central nervous system characterized by three broad bands that are joined laterally, one dorsal (i.e., cerebral ganglia and commissure) and two ventral (i.e., pedal, anterior; palliovisceral, posterior) to the esophagus (Owen, 1832; Young, 1965).

During its evolution, the cephalopod brain increased in complexity and, in Coleoidea, became completely surrounded by a cartilaginous capsule. It attained maximum aggregation of the neural masses by fusing the supra– and suboesophageal regions, enclosed in a cartilaginous cranium, alongside expansion of the two large optic lobes (positioned behind the eyes) which extend laterally from the supraoesophageal mass. The change in position and relative volume of the different sections of the brain occurred as a result of the addition or loss of ganglia.

The neural mass forming the “brain” is subdivided into varying numbers of lobes in different species (from 12 in nautiluses to 24 in octopods, excluding the optic lobes).

Neuroanatomically the central nervous system varies between different cephalopod genera. Previous authors show that distinct differences exist between octopods and decapods, which correlate well with the different anatomies. The grades of complexity of the brain parallel the complexity of the sensory inputs received and the different behaviors controlled and exhibited (Young, 1977a; Maddock and Young, 1987; Budelmann B. U., 1995). Overall, the octopod brain is more centralized that the decapod brain i.e., its brachial and pedal lodes are joined, and the superior buccal lobe is united with the inferior frontal lobes. In addition, the brachial and pedal lobes of octopods, and their inferior frontal lobe system, are larger, reflecting the sophisticated use of their arms and tactile learning. Decapods, in contrast, have larger basal lobes and a larger, unfolded, vertical lobe. Their inferior frontal lobe system is simpler, and they have no suprabrachial commissure. Decapods also possess a ventral magnocellular commissure which is not found in octopods.

At the beginning of the twenty-first century, Clark et al. (2001) introduced the concept of a cerebrotype, defining it as a species-by-species measure of brain composition. This despite the fact that a number of studies, antecedent to Clark and coworkers, recognized that different groups of vertebrates possess specific patterns of brain composition that vary among clades and ecological niches.

Cerebrotypes have been shown, in one form or another, across a range of mammals and birds (e.g., Clark et al., 2001; Lundmark, 2001; Burish et al., 2004; Iwaniuk and Hurd, 2005; Willemet, 2012; Lewitus et al., 2014; Hamodeh et al., 2017), amphibians and fish (e.g., Charvet et al., 2010; Sylvester et al., 2010; Yopak, 2012). The degree to which phylogeny and ecology relate to species-specific cerebrotypes varies among studies and taxa examined. Despite the demonstration of cerebrotypes in vertebrates, no such analyses have been performed in invertebrates, to the best of our knowledge.

Here we attempt to explore such a possibility.

Several studies have provided a considerable amount of quantitative data on the brains of cephalopod molluscs (Wirz, 1959; Frösch, 1971; Maddock and Young, 1987). Nixon and Young's effort (lasting 30 years) to collect and compare the “brains and lives” of cephalopods further stimulated interest in this field of research (Nixon and Young, 2003).

Wirz, however, was the first to compare quantitative data of the brain of 34 species of cephalopods although her pioneering study was restricted to sub-adult and adult individuals from the Mediterranean Sea (Wirz, 1959). Frösch (1971) extended Wirz's work by calculating the volumes of the brain lobes in “Schlüpfstadien” (i.e., hatchlings) of ten species of Mediterranean cephalopods. Finally, Maddock and Young (1987) assembled the largest data set available on quantitative information of the brain in cephalopods, determining the volumes of the lobes of the brain for 63 cephalopod species. Like Wirz (1959) and Frösch (1971), the values were expressed as percentages of brain volume, but in addition to the two previous studies, Maddock and Young utilized species from more varied locations, including several deep-sea forms.

In our view, cerebrotypes are identifiable in this taxon. Their evolution could be related to a number of factors. First, phylogenetic constraints could largely dictate brain composition. In this case, closely related species should have a similar brain composition or architecture. Second, developmental constraints could exert the strongest influence on cephalopod brain composition. Constraining factors could include the developmental state of hatchlings, whether or not the species undergoes metamorphosis, the habitat that the eggs are deposited on, to mention some. Third, behavior and ecology could be instrumental in determining cephalopod “cerebrotypes” such that species occupying similar niches exhibit similar brain composition. Similarity reflecting niche type has been found in vertebrates (e.g., Gonzalez-Voyer et al., 2009b; Schuppli et al., 2016; Hamodeh et al., 2017; Kamhi et al., 2019) and it is reasonable to assume that it could also occur in Cephalopoda, the invertebrates with the highest degree of brain centralization.

In this study we focus on the third factor, behavior and ecology. We aim to relate the cephalopod cerebrotypes to their “adaptive” characters and niches that they occupy. Although we recognize that the other two constraints, phylogenetic, and developmental, could play an important role in the “evolution” of cerebrotypes, these aspects are not addressed in our study. Here we analyzed existing data available for cephalopod brain organization and considered the relative size of five major brain “functional” areas in relation to ecological variables in different species.

## Materials and Methods

### Data Set

Quantitative data of the brain of various cephalopod species were obtained from the three aforementioned studies (Wirz, 1959; Frösch, 1971; Maddock and Young, 1987; see Table 1) and compiled to build the “brain” dataset (see Supplementary Table 3). *Nautilus* was excluded *a priori* because of the differences in the nervous system with respect to that of coleoids (Young, 1965; for review see also: Budelmann B. U., 1995; Nixon and Young, 2003). *Sepiola* sp. and *Sepietta petersi* (included in Frösch, 1971) were also excluded as the data for these two species were based on juvenile specimens.

**Table 1:** List of species (√, *N* = 81) included in the three published reports including quantitative data of brains in cephalopods (Wirz, 1959; Frösch, 1971; Maddock and Young, 1987).

**Order**	**Suborder**	**Family**	**Subfamily**	**Current Species name**	**Wirz (1959)**	**Frösch (1971)**	**Maddock and Young (1987)**	**Nixon and Young (2003)**	**Lindgren et al. (2012)**
Nautilida		Nautilidae		*Nautilus pompilius*			**√**		
Spirulida		Spirulidae		*Spirula spirula*			**√**		**Y**
Sepiida		Sepiidae		*Sepia officinalis*	**√**	**√**	**√**		**Y**
Sepiida		Sepiidae		*Sepia elegans*	**√**				
Sepiida		Sepiidae		*Sepia orbignyana*	**√**				
Sepiida		Sepiolidae	Sepiolinae	*Sepiola* sp.		**√**			
Sepiida		Sepiolidae	Sepiolinae	*Sepiola rondeletii*	**√**		**√**		
Sepiida		Sepiolidae	Sepiolinae	*Sepiola affinis*	**√**				**Y**
Sepiida		Sepiolidae	Sepiolinae	*Sepiola robusta*	**√**				**Y**
Sepiida		Sepiolidae	Sepiolinae	*Sepietta oweniana*	**√**	**√**			
Sepiida		Sepiolidae	Sepiolinae	***Sepietta petersi*****^a^**		**√**			
Sepiida		Sepiolidae	Rossiinae	*Rossia macrosoma*	**√**				**Y**
Sepiida		Sepiolidae	Rossiinae	*Neorossia caroli*		**√**	**√**		
Sepiida		Sepiolidae	Heteroteuthinae	*Heteroteuthis (Heteroteuthis) dispar*	**√**		**√**		
Myopsida		Loliginidae		*Loligo vulgaris*	**√**	**√**			**Y**
Myopsida		Loliginidae		***Loligo (Alloteuthis) media*****^c^**	**√**	**√**	**√**		
Myopsida		Loliginidae		*Alloteuthis subulata*	**√**				
Myopsida		Loliginidae		*Lolliguncula (Lolliguncula) brevis*			**√**		**Y**
Myopsida		Loliginidae		*Sepioteuthis sepioidea*			**√**		
Myopsida		Loliginidae		*Pickfordiateuthis pulchella*			**√**		
Myopsida		Loliginidae		*Loligo forbesii*			**√**		**Y**
[unassigned]**^b^**		Bathyteuthidae		*Bathyteuthis* sp.			**√**		
[unassigned]**^b^**		Chtenopterygidae		*Chtenopteryx sicula*	**√**		**√**		**Y**
Oegopsida		Architeuthidae		*Architeuthis dux*			**√**		**Y**
Oegopsida		Brachioteuthidae		*Brachioteuthis riisei*	**√**				
Oegopsida		Chiroteuthidae		*Chiroteuthis veranii veranii*			**√**		**Y**
Oegopsida		Chiroteuthidae		*Grimalditeuthis bonplandii*			**√**		**Y**
Oegopsida		Cranchiidae	Cranchiinae	*Cranchia scabra*			**√**		**Y**
Oegopsida		Cranchiidae	Cranchiinae	*Leachia pacifica*			**√**		
Oegopsida		Cranchiidae	Taoniinae	*Taonius pavo*			**√**		**Y**
Oegopsida		Cranchiidae	Taoniinae	*Galiteuthis glacialis*			**√**	**√**	
Oegopsida		Cranchiidae	Taoniinae	*Helicocranchia papillata*			**√**		
Oegopsida		Cranchiidae	Taoniinae	*Bathothauma lyromma*			**√**	**√**	
Oegopsida		Cranchiidae	Taoniinae	*Sandalops melancholicus*			**√**		
Oegopsida		Cranchiidae	Taoniinae	*Egea inermis*			**√**		
Oegopsida		Cranchiidae	Taoniinae	*Megalocranchia* sp.			**√**		
Oegopsida		Cranchiidae	Taoniinae	*Teuthowenia megalops*			**√**		**Y**
Oegopsida		Cycloteuthidae		*Discoteuthis laciniosa*			**√**		**Y**
Oegopsida		Enoploteuthidae		*Abralia (Asteroteuthis) veranyi*	**√**				**Y**
Oegopsida		Enoploteuthidae		*Abraliopsis (Abraliopsis) morisii*	**√**		**√**	**√**	
Oegopsida		Gonatidae		*Gonatus (Gonatus) fabricii*			**√**		**Y**
Oegopsida		Histioteuthidae		*Histioteuthis miranda*			**√**		**Y**
Oegopsida		Joubiniteuthidae		*Joubiniteuthis portieri*			**√**		**Y**
Oegopsida		Lycoteuthidae	Lycoteuthinae	*Lycoteuthis lorigera*			**√**		**Y**
Oegopsida		Mastigoteuthidae		*Mastigoteuthis schmidti*			**√**		
Oegopsida		Neoteuthidae		*Neoteuthis thielei*			**√**		**Y**
Oegopsida		Octopoteuthidae		*Octopoteuthis sicula*	**√**				**Y**
Oegopsida		Octopoteuthidae		*Octopoteuthis danae*			**√**		
Oegopsida		Ommastrephidae	Illicinae	*Illex illecebrosus*			**√**		
Oegopsida		Ommastrephidae	Illicinae	*Illex coindetii*	**√**				**Y**
Oegopsida		Ommastrephidae	Todarodinae	*Todarodes sagittatus*	**√**				
Oegopsida		Ommastrephidae	Todarodinae	*Todaropsis eblanae*	**√**				**Y**
Oegopsida		Onychoteuthidae		*Onychoteuthis banksii*	**√**		**√**		**Y**
Oegopsida		Onychoteuthidae		*Ancistroteuthis lichtensteinii*	**√**				**Y**
Oegopsida		Pyroteuthidae		*Pyroteuthis margaritifera*	**√**		**√**		
Oegopsida		Pyroteuthidae		*Pterygioteuthis giardi*			**√**		**Y**
Octopoda	Cirrata	Cirroteuthidae		*Cirroteuthis* sp.			**√**		
Octopoda	Cirrata	Cirroteuthidae		*Cirrothauma murrayi*			**√**		**Y**
Octopoda	Cirrata	Opisthoteuthidae		*Opisthoteuthis* sp.			**√**		
Octopoda	Cirrata	Opisthoteuthidae		*Grimpoteuthis* sp.			**√**		
Octopoda	Incirrata	Argonautidae		*Argonauta argo*	**√**	**√**	**√**		
Octopoda	Incirrata	Alloposidae		*Haliphron atlanticus*			**√**		**Y**
Octopoda	Incirrata	Tremoctopodidae		*Tremoctopus violaceus*	**√**		**√**		**Y**
Octopoda	Incirrata	Ocythoidae		*Ocythoe tuberculata*	**√**		**√**		
Octopoda	Incirrata	Eledonidae		*Eledone moschata*	**√**		**√**		
Octopoda	Incirrata	Eledonidae		*Eledone cirrhosa*	**√**	**√**			**Y**
Octopoda	Incirrata	Octopodidae		*Octopus vulgaris*	**√**	**√**	**√**		**Y**
Octopoda	Incirrata	Octopodidae		*Octopus bimaculatus*			**√**		
Octopoda	Incirrata	Octopodidae		*Octopus salutii*	**√**		**√**		
Octopoda	Incirrata	Octopodidae		*Macrotritopus defilippi*	**√**		**√**		
Octopoda	Incirrata	Octopodidae		*Callistoctopus macropus*			**√**		
Octopoda	Incirrata	Octopodidae		*Pteroctopus tetracirrhus*	**√**		**√**		
Octopoda	Incirrata	Octopodidae		*Scaeurgus unicirrhus*	**√**		**√**		
Octopoda	Incirrata	Enteroctopodidae		*Enteroctopus dofleini*			**√**		**Y**
Octopoda	Incirrata	Amphitretidae	Amphitretinae	*Amphitretus* sp.			**√**		
Octopoda	Incirrata	Amphitretidae	Bolitaeninae	*Japetella* sp.			**√**		**Y**
Octopoda	Incirrata	Amphitretidae	Bolitaeninae	***Eledonella sp***.**^d^**			**√**		
Octopoda	Incirrata	Amphitretidae	Vitreledonellinae	*Vitreledonella richardi*			**√**		**Y**
Octopoda	Incirrata	Bathypolypodidae		*Bathypolypus sponsalis*	**√**		**√**		
Octopoda	Incirrata	Bathypolypodidae		***Benthoctopus piscatorum*****^e^**			**√**		
Vampyromorpha		Vampyroteuthidae		*Vampyroteuthis infernalis*			**√**		**Y**

*An orange tick (**√**) marks the work considered in the final data set (n = 78). Updates (**√**) on the measurements of the proportions of some lobes are provided by Nixon and Young (2003). Information on the taxonomy and current species nomenclature is given (WoRMS Editorial Board, 2020). Whenever the original species has been reassigned this is indicated in a footnote to the table, and the original species names are in boldface*.

*The last column marks (**Y**) species that resulted included by Lindgren et al. (2012) for obtaining the maximum-likelihood tree for cephalopods based on multiple loci. To be conservative we considered correspondence between Japetella sp. (Maddock and Young, 1987) and Japetella diaphana (Lindgren et al., 2012)*.

a *Originally attributed to Sepietta petersii by Frösch (1971), the species is currently “unaccepted” and considered synonym of Sepietta oweniana (source: WoRMS Editorial Board, 2020)*.

b *The Order for these species is reported as “unassigned” and attributed to the Superorder Decapodiformes, but these are closely related to oegopsid squid*.

c *Originally attributed to Loligo (Alloteuthis) media by Authors, the species is currently “unaccepted” with accepted name Alloteuthis subulata (source: WoRMS Editorial Board, 2020)*.

d *Originally attributed to Eledonella sp. by Nixon and Young (2003), the genus and species included is currently “unaccepted” with accepted name Bolitaena (source: WoRMS Editorial Board, 2020)*.

e *Originally attributed to Benthoctopus piscatorum by Nixon and Young (2003), the species is currently “unaccepted” with accepted name Bathypolypus bairdii (source: WoRMS Editorial Board, 2020). However the actual species to which this name was likely attributed is Muusoctopus normani (see Allcock et al., 2006)*.

The final data set comprised 78 species, grouped in 33 families and six orders.

The main issue with volumetric analysis in cephalopod brains is the variation in the volume of the brain, and of the single lobes within it, with the size and age of the individual (e.g., Packard and Albergoni, 1970; Frösch, 1971; Shigeno et al., 2001a). In addition, there is a general consensus that there is not a cephalopod “reference” or “type” body size at maturity, as occurs in many vertebrate species (see also Discussion). To circumvent these problems, we utilized only the a-dimensional measurements (percentages) of the different sections of the brain from Wirz (1959) and Maddock and Young (1987).

Maddock and Young (1987) grouped single brain lobes into functional sets, allowing for comparisons between taxa with different numbers of lobes. Wirz (1959) however did not consider the lobes of the brain in terms of functional sets.

In order to combine the data included in the two papers we (i) searched for correspondence between lobes, (ii) grouped each lobe in its functional set, and (iii) summed-up the values of the lobes per functional set.

Eight functional sets and their corresponding relative brain sizes were identified (Supplementary Table 2).

The values of the different functional sets were recalculated as proportions relative to the volume of the whole brain (i.e., the sum of supra- and sub-esophageal masses and optic lobes), without altering the order of magnitude of the data within and between functional sets and providing values that prevented overemphasizing certain values, such as the volume of the optic lobes, in the standardization procedures required by the assumptions of the clustering technique (Everitt, 1993; Everitt et al., 2001).

In order to circumvent the intrinsic differences in the brain size values of the two data sets, we arbitrarily chose the data of Maddock and Young (1987) for species in common to both papers rather than calculating the average of the percentages given in the two works.

### Ecological Variables

In order to relate the relative size of the different brain areas to ecological and life-history of different cephalopod species, we collated information available on the ecology, distribution, life history, behavior, morphological adaptations and reproductive strategies (see Supplementary Information for details) of the 78 taxa for which we had brain size data. In particular, we considered:

- ***Method of locomotion***: as indicator of the potential of a species to spread and adapt to new environments;
- ***Feeding habits***: whether a species has adapted to become a generalist or a specialist;
- ***Development***: as a potential indicator or the relative dispersal of hatchlings following spawning;
- ***Reproduction***: i.e., mating/spawning;
- ***Habitat***: i.e., vertical and horizontal distribution, that potentially affects gene flow and dispersal.

In total, more than 15 categories of data counting a total of 130 variables constituted the final matrix of life-habits data. These data were utilized as life-adaptation descriptors of the species considered (See Supplementary Information and Supplementary Table 3).

### Analyses of Data

The combination of relative brain size data and “ecological variables” for each of the 78 cephalopod species herein considered (Supplementary Table 3), represents our database, i.e., a multi-dimensional matrix including data on the diversity of brain lobe' size and life history attributes of 78 species representing different cephalopod families.

Data analysis followed approaches included in Zar (1999) and Everitt et al. (2001). In brief, we utilized principal component analysis (PCA) to reduce the dimensions of the anatomical data (i.e., eight functional sets), followed by Varimax rotation. The resulting factor scores (regression method, see Gorsuch, 1983) and the ecological variables (see list above and Supplementary Information) were analyzed through a hierarchical cluster analysis using Ward's method (Ward, 1963).

In analogy to similar studies carried out in vertebrates (e.g., Iwaniuk and Hurd, 2005), we selected PCA to help reduce the number of variables into “components” thus exploring internal structure of the data and possibly the variance. This is considered a useful method for examining cerebrotypes (see for example discussion in Iwaniuk and Hurd, 2005). As mentioned above, we also performed a cluster analysis. We selected Ward's Hierarchical method (Ward, 1963) as a general agglomerative hierarchical clustering procedure, where the criterion for choosing the pair of clusters to merge at each step is based on the optimal value of an objective function (*sensu* Ward, 1963). This is a method utilized in several “social” science and behavioral studies, and has the advantage of not being related to any “phylogeny” (as in our case), but linked to the variability/characteristic and structure of data.

Non parametric analysis of variance (Kruskal Wallis test) followed by *post hoc* pairwise comparisons (Dunn, 1964; Zar, 1999) was utilized to compare mean proportions of functional brain sets belonging to species attributed to different clusters.

All statistical analyses were carried out using SPSS (rel. 18.0 PASW—Predictive Analytics SoftWare, IBM, 2010), except for the hierarchical cluster analysis (CLUSTAN, Wishart, 1987).

### Phylogenetic PCA and Further Analysis of Data

This study is not primarily aimed at finding a direct link between brain diversity in cephalopods by means of a phylogenetic analysis. However, to attempt to control for phylogenetic dependence/independence of traits here considered (brains' diversity) and possibly ruling out bias in detecting relationships and inaccurate estimates of correlations (Rezende and Diniz-Filho, 2012; see also Adams and Collyer, 2017) we ran phylogenetic principal component analysis (pPCA, Revell, 2009). This aims to explore association between brain volume proportions and species by taking into account the phylogenetic relationship between species.

We utilized as reference tree (source of “phylogenetic signal” for our data) the multigene phylogeny based on maximum likelihood analysis by Lindgren et al. (2012); this is considered one of the most complete cephalopod phylogeny published so far. The original tree comprises 188 taxa (see Figure 1 in Lindgren et al., 2012).

Despite the large number of species included by Lindgren et al. (2012) only 38 species overlapped with the list of 78 cephalopods considered in our dataset (see Table 1 and Supplementary Table 6).

The phylogenetic PCA was carried out by pruning the original tree by Lindgren et al. (2012) to obtain one including exclusively the 38 species common to both datasets (Supplementary Figure 1). The pruned tree was utilized as input phylogeny for “phyloPCA” included in the phytools package (https://CRAN.R-project.org/package=phytools; September, 2020). For phyloPCA we set: method = “lambda,” mode = “corr,” rotate = “varimax.” Data for the brain functional sets were included, for corresponding species.

After pPCA, scores for the first three components were extracted, followed by a further cluster analysis (see Supplementary Information section: “Considerations taken for the phylogenetic PCA and subsequent analysis”).

## Results

### Cephalopod Relative Brain Size

**Figure 1:**
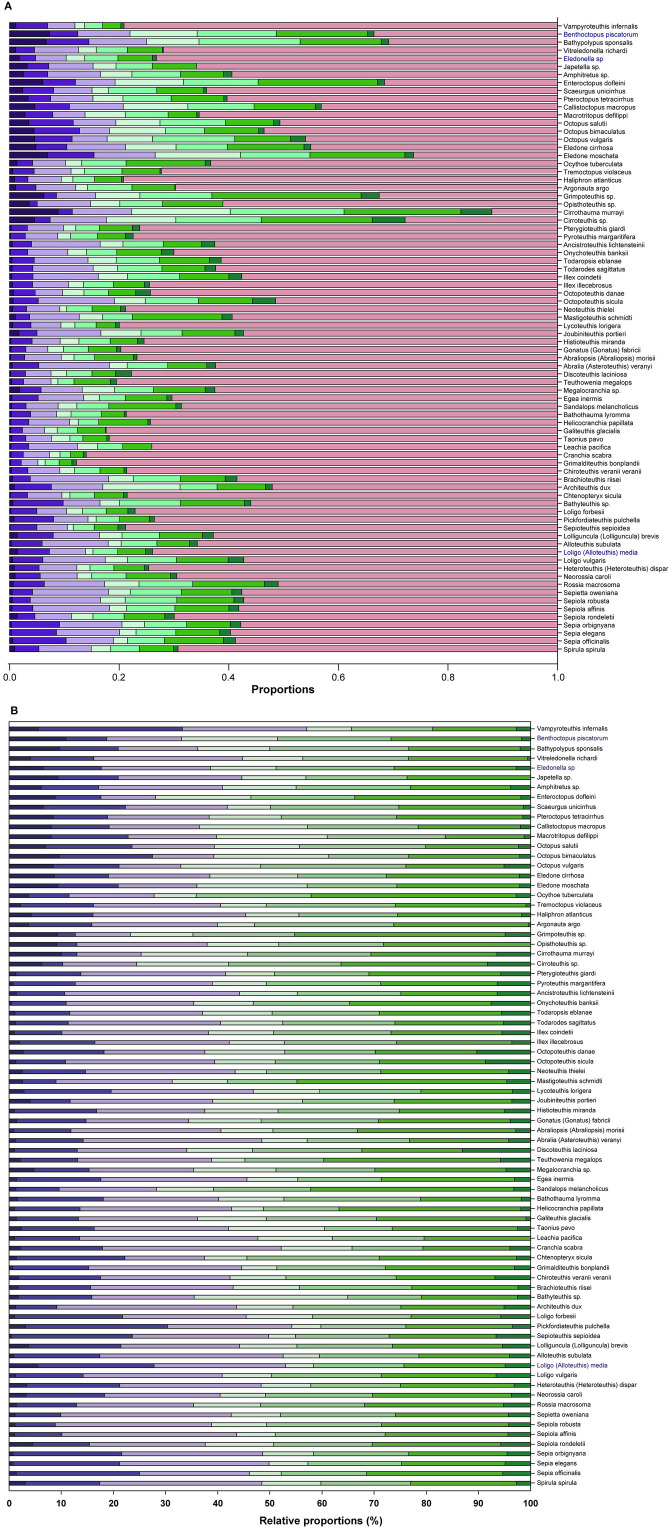
Proportions of the eight functional sets on the whole brain **(A)** in the 78 species of cephalopods considered in this study (see also Supplementary Table 3 for values). The functional sets (see Supplementary Table 2 for details) are color coded. For supraoesophageal mass: INFF (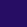), VERT (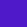), BASAL (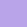); for suboesophageal mass: BRAC (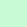), PEDAL (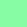), PALL (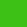), CHRF (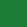); for optic lobes (OPTIC, 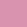). **(B)** 100% Stack Bar graph of the relative proportions of the functional brain sets excluding OPTIC, to illustrate variability of brain areas identified in supra- and suboesophageal masses between species.

Figure 1 presents an overview of the different proportions of brain areas (i.e., functional sets; see also Supplementary Table 2) in the 78 species of cephalopods considered. We also include a stacked bar summary of the relative proportions of brain functional sets in different species, excluding the contribution of the optic lobes (Figure 1B).

From these data PCA allowed three components to be identified, which together account for 87.3% of the total variance (Table 2). The first component (62.3% variance) is positively correlated with “inferior frontal” (INFF), “brachial” (BRAC), “pallial” (PALL) and “pedal” (PEDAL) lobe functional sets, but negatively correlated with the optic lobe. The second component accounts for roughly 13% of the variance and is correlated with “basal” (BASAL) and “chromatophore” (CHRF) lobes. The vertical lobe system (VERT) appears in the third component, which explains 12% of the total variance.

**Table 2:** Principal components analysis for the relative proportions of the eight brain-functional sets (for abbreviations and description see Supplementary Table 2) of the 78 species of cephalopods considered in this study (see also Borrelli, 2007).

	**Components**		
	**1**	**2**	**3**
INFF	**0.961**	−0.033	0.107
BRAC	**0.881**	0.215	0.158
OPTIC	**−0.849**	−0.468	−0.238
PALL	**0.838**	0.344	−0.039
PEDAL	**0.826**	0.420	0.193
BASAL	0.116	**0.859**	0.265
CHRF	0.362	**0.753**	−0.182
VERT	0.178	0.071	**0.949**
Eigenvalue	4.99	1.03	0.97
% Variance	62.30	12.90	12.10

*For relative proportions of brain sets see also Figures 1A,B and Supplementary Table 3. Bold type indicates correlations of variables with the principal components >0.50*.

**Figure 2:**
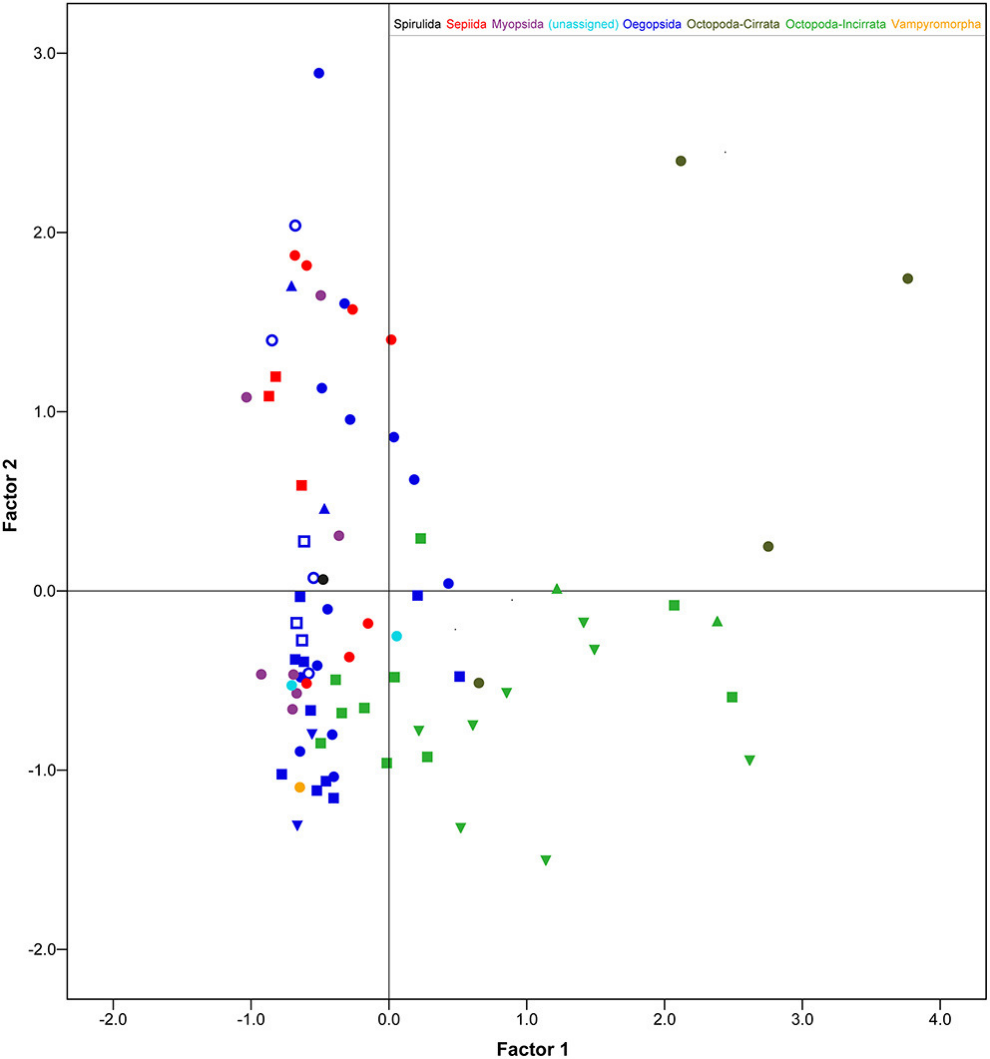
Scatter plot of factor score values (after regression following PCA, Gorsuch, 1983) of the 78 cephalopod species belonging to the six Orders (color-coded) considered in this study. Different symbols group various taxa for a given order, whenever applicable (see Supplementary Information and Supplementary Table 4 for details). Cirrata and Incirrata are coded with different grades of green. Only the first two factor scores are considered here. See text for details and Table 3 for relative proportions of cephalopod brains between species allocated in different clusters.

These results confirm the view that cephalopods possess largely diversified brains; a diversity particularly marked when the PCA scores were plotted against the six orders included in the data set (Figure 2; see also Table 3). Myopsid and oegopsid species included in this study are best separated by the second component (BASAL and CHRF), possibly due to the large contribution of the basal lobe system and chromatophore and fin lobes to the relative proportion of brain areas in these species. For similar reasons, octopods are widely distributed along the first component (correlated with the inferior frontal, brachial, pallial and pedal lobes, and negatively with the optic lobes), with the exception of two species (*Cirroteuthis* sp. and *Cirrothauma murrayi*) which are also widely separated by the second component, and therefore may be considered as outliers. This result may be linked to the fact that the two species had (in our data set, and to the best of our knowledge) the smallest optic lobes (38.6 and 13.5% of the brain, respectively; Supplementary Table 3; see also Maddock and Young, 1987). We can speculate that the vertical lobe system occupies a distinct component (the third) because of the large variability in relative proportions, within orders, of this structure: its size varies “more than four times among the decapods and six times in octopods” (Maddock and Young, 1987, p. 749).

**Table 3:** The mean proportions of the eight brain regions (brain-functional sets) for each of the ten clusters identified after the hierarchical cluster analysis (see Figure 3).

**Cluster**	**Taxa**	**Species**	**INFF**	**VERT**	**BASAL**	**BRAC**	**PEDAL**	**PALL**	**CHRF**	**OPTIC**
1 (*n* = 5)	Spirulidae Sepiolidae Brachioteuthidae Enoploteuthidae	*Spirula spirula* *Heteroteuthis (Heteroteuthis) dispar* *Brachioteuthis riisei* *Abralia (Asteroteuthis) veranyi* *Abraliopsis (Abraliopsis) morisi*	0.617 (0.243–0.991)	3.909 (2.706–5.112)	10.061 (5.772–14.349)	3.190 (2.094–4.286)	5.886 (3.341–8.430)	6.827 (5.593–8.061)	1.212 (0.427–1.998)	68.298 (58.633–77.963)
2 (*n* = 3)	Sepiidae	*Sepia officinalis* *Sepia elegans* *Sepia orbignyana*	0.414 (0.055–0.773)	8.919 (7.041–10.797)	10.529 (6.501–14.557)	3.214 (1.196–5.231)	7.233 (6.049–8.417)	8.912 (4.911–12.913)	2.025 (1.653–2.397)	58.755 (56.430–61.080)
3 (*n* = 5)	Sepiolidae	*Sepiola rondeletii* *Sepiola affinis* *Sepiola robusta* *Sepietta oweniana* *Rossia macrosoma*	0.698 (0.211–1.184)	3.946 (2.705–5.188)	11.658 (7.886–15.429)	4.369 (2.879–5.859)	8.610 (6.529–10.691)	9.985 (7.444–12.526)	1.937 (1.509–2.365)	58.797 (50.247–67.347)
4 (*n* = 6)	Loliginidae	*Loligo vulgaris* *Loligo (Alloteuthis) media* *Alloteuthis subulata* *Lolliguncula (Lolliguncula) brevis* *Sepioteuthis sepioidea* *Loligo forbesii*	0.678 (0.062–1.2934	5.535 (4.827–6.242)	8.236 (5.189–11.284)	2.646 (1.304–3.987)	5.835 (3.762–7.908)	6.092 (3.853–8.331)	1.706 (1.055–2.357)	69.272 (60.224–78.320)
5 (*n* = 6)	Architeuthidae Ommastrephidae	*Architeuthis dux* *Illex illecebrosus* *Illex coindetii* *Todarodes sagittatus* *Todaropsis eblanae* *Onychoteuthis banksii*	0.481 (0.241–0.721)	4.217 (2.872–5.562)	9.329 (7.164–11.494)	5.863 (1.501–10.226)	7.219 (5.553–8.886)	8.075 (6.716–9.434)	1.823 (1.203–2.444)	62.992 (54.435–71.549)
6 (*n* = 6)	Chiroteuthidae Cranchiidae	*Chiroteuthis veranii veranii* *Grimalditeuthis bonplandii* *Taonius pavo* *Galiteuthis glacialis* *Megalocranchia sp*. *Teuthowenia megalops*	0.586 (-0.049–1.221)^a^	2.604 (1.809–3.399)	5.011 (3.397–6.626)	2.812 (1.003–4.621)	3.890 (2.019–5.760)	5.355 (2.814–7.896)	0.811 (0.206–1.416)	78.932 (69.909–87.955)
7 (*n* = 6)	Cranchiidae	*Cranchia scabra* *Leachia pacifica* *Helicocranchia papillata* *Bathothauma lyromma* *Sandalops melancholicus* *Egea inermis*	0.339 (0.262–0.417)	3.264 (2.336–4.193)	6.666 (4.775–8.557)	2.688 (1.816–3.559)	4.395 (2.888–5.901)	6.749 (2.992–10.506)	0.561 (0.174–0.947)	75.336 (68.749–81.924)
8 (*n* = 4)	Gonatidae Tremoctopodidae Amphitretidae	*Gonatus (Gonatus) fabricii* *Tremoctopus violaceus* *Japetella sp*. *Eledonella sp*.	1.468 (-0.625–3.561)^b^	3.375 (2.374–4.376)	6.107 (3.344–8.869)	3.206 (1.991–4.422)	6.026 (4.377–7.675)	6.609 (4.643–8.576)	0.447 (-0.166–1.060)^c^	72.761 (63.750–81.772)
9 (*n* = 7)	Octopodidae Enteroctopodidae	*Octopus vulgaris* *Octopus bimaculatus* *Octopus salutii* *Octopus defilippi* *Callistoctopus macropus* *Pteroctopus tetracirrhus* *Enteroctopus dofleini*	4.212 (3.208–5.216)	6.383 (4.979–7.787)	7.181 (5.825–8.538)	9.089 (6.767–11.411)	10.924 (8.109–13.738)	10.944 (6.185–15.702)	1.187 (0.489–1.885)	50.080 (39.674–60.486)
10 (*n* = 4)	Eledonidae Bathypolypodidae	*Eledone moschata* *Eledone cirrhosa* *Bathypolypus sponsalis* *Benthoctopus piscatorum*	6.427 (4.666–8.187)	6.779 (4.208–9.349)	10.478 (9.382–11.575)	11.649 (6.972–16.326)	13.731 (7.699–19.763)	15.695 (13.179–18.210)	1.332 (1.038–1.626)	33.908 (21.171–46.645)

*For each cluster the table also includes: number of species, taxa represented (attribution to Families, see Table 1 for reference and current taxonomy and nomenclature), list of species, the 95% Confidence Intervals for each structure (in parentheses). Whenever the lower bound of the 95% Confidence Interval for the mean is negative, we also report as a footnote minimum and maximum values. The mean values are suggested to represent different cerebrotypes for the group of cephalopod species considered. Analysis of differences between mean proportions of cerebrotypes are shown in Supplementary Table 5. For abbreviations identifying cephalopod brain-functional sets see Supplementary Table 2*.

a Minimum = 0.222; maximum = 1.807.

b Minimum = 0.305; maximum = 3.209.

c *Minimum = 0.000; maximum = 0.793*.

### Correlating Cephalopod Cerebrotypes Their Life-Styles and Other Adaptations

To search for any possible relationship between the components extracted from the PCA depicting brain diversity in cephalopod and cephalopod' life adaptation descriptors (the “ecological” variables; see Supplementary Information) of the various organisms we considered, we carried out a hierarchical cluster analysis as an attempt to summarize patterns of similarity/dissimilarity among species.

The clustering was carried out only on 52 species out of the original list (*n* = 78): 26 species were excluded for missing data (233 null values, 2.3% of the whole dataset) in 32 of the 130 total number of variables/states included as indicators of cephalopod “life-style/adaptations.” We cannot ignore that the 26 species not included in the cluster analysis might have provided a different grouping and/or the identification of additional clusters (see discussion around missing data in Supplementary Information “Number of species included in the final clustering and reasons for exclusions”).

**Figure 3:**
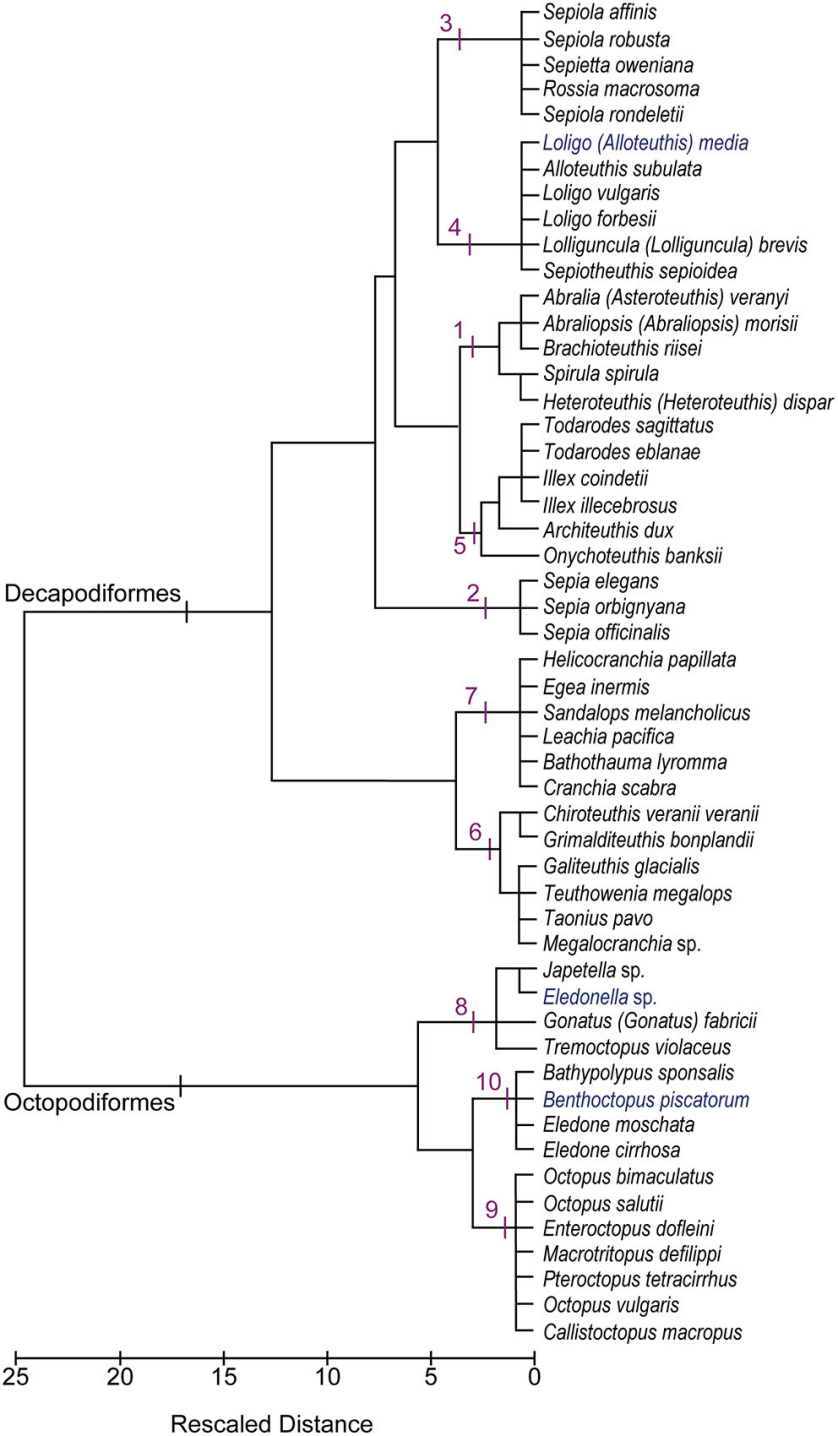
Dendrogram after Ward's hierarchical clustering showing the relationships among cephalopod species (*N* = 52) based upon the data set (*N* = 78). The clusters recognized are numbered from 1 to 10 and described in the text. See also Table 3 and Supplementary Table 5.

The cluster analysis yielded a dendrogram with ten distinct clusters (labeled from 1 to 10 in Figure 3). Table 3 summarizes mean values of the proportions for each of the brain regions belonging to the groups of species identified through hierarchical clustering to illustrate differences on the resulting “cerebrotype.”

A short description for each cluster follows in the following paragraphs. Hereunder, the species are referred according to original names as indicated by Authors (Wirz, 1959; Maddock and Young, 1987); for current valid taxonomy refer to Table 1.

The first group of species (*Cluster 1)* consists of a very diverse assemblage of species (i.e., *Spirula spirula, Heteroteuthis dispar, Brachioteuthis riisei, Abralia veranyi, Abraliopsis morisi*), belonging to three distinct orders of cephalopods (Spirulida, Sepiolida, Teuthida). The supraoesophageal mass of these species is characterized by a very small “inferior frontal” (INFF), medium-sized VERT, and relatively large “basal” (BASAL) lobes. The functional brain sets pertaining to the suboesophageal mass are, on average, very similar within the species included in this cluster. The chromatophore lobe (the fin lobe is the major contributor to CHRF in most of these species) is almost absent.

Species in this cluster possess relatively large optic lobes representing about 65% of total brain size.

All five species are known to be oceanic (mesopelagic), achieving buoyancy by active swimming (dynamic lift) with the aid of broad fins, with the exception of *Spirula* which has near-neutral buoyancy with a chambered shell and short, subterminal fins. Their geographic distribution is variable, ranging from 3 to 10 Large Marine Ecosystems (LME; see Sherman and Duda, 1999 see also Supplementary Information), and, as they are reported to feed almost exclusively on pelagic crustaceans, the species should be considered diet specialists, although the diet is slightly richer in *H. dispar* (in *H. dispar*, the lower beak is characterized by a medium-narrow rostrum, curved hood, shallow or absent notch, and obtuse or right jaw angle). Finally, this group clusters the only species (from the 78 analyzed) with a “*Sthenoteuthis* type” reproductive strategy (sensu Nigmatullin and Laptikhovsky, 1994; see also Nesis, 2002) and multiple spawners *sensu* Rocha et al. (2001). The eggs are small, single, unencapsulated and pelagic (laid at or near the surface).

*Cluster 2* groups the three *Sepia* species: *S. officinalis, S. elegans*, and *S. orbignyana*. In this case, the supraoesophageal mass is characterized by a very small inferior lobe system, and large vertical and basal lobes. Typical of the decapod subesophageal mass, the brachial lobe is not particularly developed (PEDAL and PALL being on average similar to the other clusters). However, in these species prominent chromatophore (about 2%) and fin (3%) lobes exist, accompanied by moderately large optic lobes (about 60% of brain size). All three species are reported as having “near-neutral buoyancy” via a chambered shell, with fringed fins. They live in coastal, benthic habitats (mainly littoral and continental shelf), and are quite widely distributed (occurring in 10 LMEs on average). Cuttlefish are known to have a relatively broad diet breadth (i.e., generalists), and the lower beaks are characterized by a long and broad rostrum, curved hood, shallow notch, and curved jaw angle. All the three species have an “*Illex*-type” reproductive strategy (*sensu* Nesis, 2002) and are intermittent terminal spawners (*sensu* Rocha et al., 2001). The eggs are mostly intermediate in size, single, encapsulated and laid in batches on the bottom.

The third group (*Cluster 3*; Figure 3) includes all benthic bobtail squids in our dataset: *Sepiola rondeletii, S. affinis, S. robusta, Sepietta oweniana*, and *Rossia macrosoma*. In these species the supraesophageal mass is characterized by a relatively small INFF (the exception being *S. rondeleti*), large BASAL and moderately large VERT. Within the suboesophageal mass, the PEDAL and PALL are prominent in comparison to other decapods. The chromatophore and fin lobes are similar in size to those observed in *Sepia* species (see *Cluster 2*). The five species included in *Cluster 3* possess moderately large optic lobes (about 60% of the entire brain). They are considered to be “dense” (see Supplementary Information and Supplementary Table 3), bottom living, finned species, inhabiting both coastal waters (littoral and continental shelf) and the slope (bathybenthic). *Sepiola rondeletii, S. oweniana*, and *R. macrosoma* occupy a moderately extended geographical range while *S. affinis* and *S. robusta* are known to be distributed within a slightly narrower range. Data available to us suggest the diet is restricted to crustaceans (with the exception of *Rossia*); the lower beak being characterized by a long and moderately broad rostrum, curved hood, absent notch, and obtuse jaw angle. The life-style and reproductive strategies (sensu: Nigmatullin and Laptikhovsky, 1994; Nesis, 2002), spawning pattern and egg morphology of the five species is the same as for cuttlefish (as for *Cluster 2*).

*Cluster 4* comprises six coastal loliginids (*Loligo vulgaris, L. forbesii, L*. (*Alloteuthis*) *media, L*. (*Alloteuthis*) *subulata*^1^, *Lolliguncula brevis*, and *Sepioteuthis sepioidea*). To compensate for their dense body tissues, they swim actively (dynamic lift), by flapping their elongated fins to remain afloat. These squids inhabit neritic and shallow waters (epi-mesopelagic). Species included in this cluster are widely distributed across LMEs; *S. sepioidea* and *L*. (*Alloteuthis*) *media* are reported to have more restricted distributions. The supraoesophageal mass is characterized by a small inferior frontal system, with the exception of *L*. (*Alloteuthis*) *media* and *L. brevis* for which proportions are about four times those of the other species in the group. The species are reported to have large vertical lobes (as compared with other species) and basal lobes of variable relative size. Within this cluster the chromatophore lobes are smaller than the larger fin lobe (together reaching about 6% of the cerebral masses). The optic lobes are comparatively large, ranging between 57 and 78% of the total brain size. Diet is diverse among species, but is generally considered to be intermediate in breadth with *L. vulgaris* and *L. forbesii* having the most diverse diet, and *L. brevis* the most specialist diet. The lower beaks of these species have a long, broad rostrum, curved hood, shallow notch, and obtuse jaw angle. The species reproduce and spawn following similar strategies (see above and Supplementary Information) to the other neritic species (sepiids and sepiolids) although the eggs are laid on the bottom in collective capsules rather than singular capsules.

*Cluster 5* groups six squids which occupy the same zone (epi-mesopelagic) as loliginids, but which are oceanic. This cluster includes the most voracious cephalopod species, such as the giant squid (*Architeuthis dux*), the muscular, flying squids (*Illex illecebrosus, I. coindetii, Todarodes sagittatus, Todaropsis eblanae*), and the common clubhook squid (*Onychoteuthis banksii*). They occupy moderately wide geographic distributions (*O. banksii* with a particularly broad distribution). Our data sources describe these species as feeding exclusively on pelagic organisms such as crustaceans, fish, and mollusks (including cephalopods). The lower beaks have a long, narrow rostrum (except for *A. dux*), curved hood, and acute (or right) jaw angle. They are fast swimmers (with broad triangular fins) and move using dynamic lift (because they are dense), with the exception of *A. dux* (near-neutral; chemical lift; ammonium). The supraoesophageal mass has a relatively small “inferior frontal” (INFF), moderately variable in size VERT and basal lobes; chromatophore lobes quite reduced when compared with the fin lobe (with the exception of *O. banksii* in which they are equivalent in size), and optic lobes representing about 60% of the total brain size. The reproductive strategy (sensu: Nigmatullin and Laptikhovsky, 1994; Nesis, 2002) of these species is similar to that of loliginid squids (see *Cluster 4*), with the exception of *A. dux*, which like other “ammoniacal” squids, reproduces with a “*Gonatus*-type” strategy (sensu Nigmatullin and Laptikhovsky, 1994; see also Nesis, 2002).

The sixth group of species (*Cluster 6*) includes the chiroteuthid squids (*Chiroteuthis veranyi, Grimalditeuthis bonplandi*) and four Cranchiidae (*Taonius pavo, Galiteuthis glacialis, Megalocranchia* sp., *Teuthowenia megalops*); the other Cranchiidae species here considered grouped in *Cluster 7*. The six species are “near-neutral” and achieve buoyancy by chemical lift (ammonium)^2^. Their fins are short, rounded and subterminal (as in the transparent glass squids) or secondary (chiroteuthids). They are “oceanic” (epi-bathypelagic), with a quite variable geographic distribution, with the exception of *T. pavo*. The supraoesophageal mass has the smallest VERT and BASAL compared with both muscular (see clusters 4 and 5) and glass squids (see *Cluster 7*). The chromatophore lobe is reduced or absent while the fin lobe is relatively developed, when compared with species included in *Cluster 7* (see also: Maddock and Young, 1987; Nixon and Young, 2003). The optic lobes are large, representing about 80% of the entire “brain.” Diet breadth (hypothetical for many species) is listed in our data set as relatively wide, restricted to pelagic prey items (i.e., cephalopods, crustaceans, fish). The lower beak is reported as short but broad rostrum, curved hood, broad notch and obtuse jaw angle. All six species are reported to reproduce with an “*Gonatus*-type” strategy (sensu Nigmatullin and Laptikhovsky, 1994; see also Nesis, 2002) and are intermittent terminal spawners (*sensu* Rocha et al., 2001). The eggs are small (the size is hypothetical for many species) and are released as collective capsules in deep layers of the water column.

*Cluster 7* groups the six remaining Cranchiidae species (*Cranchia scabra, Leachia pacifica, Helicocranchia papillata, Bathothauma lyromma, Sandalops melancholicus, Egea inermis*). They occupy the same area of the marine realm as *Cluster 6*, and have similar modes of locomotion, reproductive and spawning patterns, egg morphology, and site of deposition. The supraoesophageal mass is characterized by the smallest INFF, but larger VERT and basal lobes compared with other cranchiids. The chromatophore lobe is reduced or absent, the fin lobe is relatively small (except in *C. scabra*); the optic lobes are fairly large, accounting for about 75% of the total brain size. The diet is hypothetical although similar lower beak morphology (variable rostrum, curved hood, variable notch, and obtuse jaw angle) to species included in *Cluster 6* would suggest similar diet breadth (see also above).

Armhook squid, *Gonatus fabricii*, the gelatinous octopuses (*Japetella* sp., *Bolitaena* sp.) and the common blanket octopus *Tremoctopus violaceus* are included in *Cluster 8*. These species are finless except for *G. fabricii*, free-swimming (near-neutral by chemical lift via lipids or chlorine) and live-in open waters, mainly occupying surface or intermediate layers (epi-mesopelagic), although bolitaenids are also reported to live in deeper waters (bathypelagic). All species are reported as widely distributed around the world's oceans (7-17 LMEs; but *G. fabricii* is currently known as restricted to the North Atlantic). In our data the diet breadth, similar to glass squids, is reported as relatively broad, with a focus on pelagic organisms, such as pteropods (molluscs), amphipods, copepods and euphausiids (crustaceans), chaetognaths and fish. Lower beak morphology is distinctive in these species: a short rostrum, curved or flat hood, notch absent. The supraoesophageal mass is characterized in these species by a small INFF (with the exception of *Japetella* sp.), small basal lobes and a medium-sized vertical lobe system. The chromatophore and fin lobes are reduced or absent. The optic lobes are large reaching about 70% of the total size of the brain. *Japetella* sp., *Bolitaena* sp., and *T. violaceus* reproduce with an “*Octopus*-type” strategy (*sensu* Nigmatullin and Laptikhovsky, 1994; see also Nesis, 2002) and are simultaneous terminal spawners (*sensu* Rocha et al., 2001), while *G. fabricii* reproduces with a “*Gonatus*-type” strategy (*sensu* Nigmatullin and Laptikhovsky, 1994; see also Nesis, 2002) and is an intermittent terminal spawner (*sensu* Rocha et al., 2001). Recent studies reported that *G. fabricii* exhibit a geographically localized reproduction, relatively uncommon for deep-water squids (Golikov et al., 2019).

*Cluster 9* groups seven species of benthic octopuses: *Octopus vulgaris, O. bimaculatus, O. defilippi^3^, O. macropus*^3^*, “Octopus” salutii, Enteroctopus dofleini, and Pteroctopus tetracirrhus*. All of them are “dense,” finless, bottom living species inhabiting coastal waters (littoral and continental shelf, with only *O. macropus, O. salutii* and *P. tetracirrhus* extending to bathybenthic layers). *Octopus vulgaris, O. bimaculatus, O. defilippi, O. macropus*^3^ have a wide geographical distribution; *O. bimaculatus, O. salutii, P. tetracirrhus*, and *E. dofleini* occupy a more restricted range (but see also Jereb et al., 2016).

The supraoesophageal mass is characterized by a large “inferior frontal” (INFF) and vertical lobe systems (VERT; although this is quite variable among species) and a well-developed chromatophore lobe (fin lobe absent). The optic lobes are moderately large as they represent about 50% of the entire brain. *Octopus vulgaris* is reported to be a generalist species, while the other taxa in this cluster are described in our dataset with a more restricted diet^4^. The lower beak is described with a short-broad rostrum, narrow curved hood, broad notch, and obtuse jaw angle. All seven species reproduce following an “Octopus-type” strategy (*sensu* Nigmatullin and Laptikhovsky, 1994; see also Nesis, 2002) and are simultaneous terminal spawners (*sensu* Rocha et al., 2001). The eggs, small-intermediate in size, are laid in clusters on the substrate (e.g., females of *O. vulgaris* lay their egg strings in their den).

*Cluster 10* groups the remaining octopods: *Eledone moschata, E. cirrhosa, Bathypolypus sponsalis, Benthoctopus piscatorum*^5^. These species (like in *Cluster 9*) are also benthic (dense body tissues) and finless. However, they are known to occupy deeper layers than the species included in *Cluster 9*; particularly an exclusively bathybenthic distribution is known for *B. sponsalis* and *B. piscatorum*. In contrast to *Cluster 9*, these species occupy a relatively restricted geographical distribution (*E. moschata* and *B. sponsalis* being even more restricted).

The supraoesophageal mass is characterized by large INFF, VERT and basal lobes. The chromatophore lobe is moderately large; the fin lobe is absent. The optic lobes are the smallest in our dataset: 26-33% of the total brain size; 45% for *E. cirrhosa*. The diet is reported as specialized^6^, with the exception of *E. cirrhosa*. The lower beak is described as short, with broad rostrum, variable curved hood, broad notch, and acute or recessed jaw angle. The reproductive and spawning strategies of the two *Eledone* species are comparable to those of shallow water octopods (see cluster 9). Contrarily, the deep-sea octopuses (*B. sponsalis* and *B. piscatorum*) are reported to be continuous spawners in our source of data. There is no consensus on the spawning pattern of deep-sea octopuses due to lack of data. The eggs of all four species are large (when compared with other octopods), and are in clusters which are laid on substrate.

At first glance, the dendrogram (Figure 3) reveals the strong division of coleoid cephalopods into Decapodiformes (clusters 1–7) and Octopodiformes (clusters 8–10), which corresponds to a high dissimilarity index (Squared Euclidean Distance coefficient = 2579.3). The sole exception to this general pattern is represented by the oegopsid squid, *Gonatus fabricii*, which is clustered with pelagic octopuses. As already described (see *Cluster 8*), this association may be explained by the fact that Armhook squids share with free-swimming octopuses the common behavior of females brooding eggs on their arms, instead of releasing them in the water column (as do other oceanic squids) or laying them on the ground (as do neritic squids). However, a more attentive analysis of the figure suggests that the separation of coleoids in two distinct lineages (i.e., decapods vs. octopods) is less clear-cut than expected. The relative affinity or relatedness among species—within and between clusters—seems largely to depend on the life adaptations they share in common (e.g., buoyancy mechanisms, habitats occupied, reproductive strategies) that, in turn, has brought about a similar differentiation in the lobes of the brain, as for our hypothesis.

For example, Bobtail squids (cluster 3) and inshore squids (cluster 4) are grouped together because they share several features in common. They both live in coastal waters (although sepiolids may reach deeper layers of the water column), have “dense” bodies and fins (see Supplementary Table 3). Both sepiolids and loliginids are intermittent terminal spawners (*Illex*-type strategy) so that the eggs are laid, in separate clutches, over a relatively long-time frame (Rocha et al., 2001). The eggs (small-intermediate in size) are laid on the substrate in batches (sepiolids) or collective capsules (loliginids). Moreover, both taxa are reported in our data-set with brains characterized by a very small “inferior frontal,” medium-sized vertical lobe system and considerable basal lobes. The optic lobes are also well developed in these species, representing roughly 60 and 70% of the total brain size in sepiolids and loliginids, respectively. The fin lobe is rather more conspicuous in loliginids which can be explained by their strictly pelagic life style, in contrast to sepiolids live mostly in contact with the bottom.

### Differences Between Cephalopod Cerebrotypes as Identified by Hierarchical Cluster Analysis

To illustrate whether the ten clusters identified after Ward's hierarchical method correspond to a characteristic “cerebrotype,” we calculated mean values of the proportions for each of the brain functional sets of species belonging to every cluster identified (Table 3). Differences between the proportions for each of the eight brain regions (brain-functional sets) shown for species belonging to the clusters identified, were significant according to Kruskal–Wallis one-way analysis of variance (Supplementary Table 5). *Post hoc* pairwise comparisons further confirmed a composite variation of cephalopod brain areas attributed to different clusters (see Supplementary Table 5.2). In particular we found significant differences for INFF (clusters 9 and 10 vs. others), VERT in more than 12 pairwise comparisons (26% of the total), BASAL (cluster 6 vs. 1-3, 5, 10; 2 vs. 7, 8; 3 vs. 6-9, to mention some; see Supplementary Table 5.2) when considering the supraoesophageal mass. Differences between areas also emerged when suboesophageal mass was considered: e.g., PEDAL - cluster 1 vs. 9, 10; cluster 3 vs. 6, 7; cluster 4 vs. 9, 10; cluster 5 vs. 6, 7; cluster 6 vs. 2, 5, 9, 10, cluster 7 vs. 3, 9, 10 (see Supplementary Table 5 for details).

### Phylogenetic PCA

The phylogenetic Principal Component Analysis (Revell, 2009) was carried out on a reduced number of species (*n* = 38; 48.7% of the total) because of missing correspondence between organisms selected by Lindgren et al. (2012) and our dataset.

The resulting first two components accounted for 77% of variance (Eigenvalues, %Variance: PC1 = 5.20, 65.0; PC2 = 0.97, 12%); a third component (eigenvalue = 0.82) was also considered accounting for a total of 88% of cumulative variance (for extracted scores see Supplementary Table 7). Because of pruning and lack of overlap between our dataset and the species considered by Lindgren et al. (2012) a further reduction in the list of species was required for the following cluster analysis (*n* = 24 out 52; 46% of the species included in Figure 3)^7^.

The resulting dendrogram (Supplementary Figure 2) shows limited similarities with the clustering of Figure 3.

In brief, the grouping corresponding to clusters 3 and 4 was almost retained, despite some loss in species (i.e., *Sepiola affinis, S. robusta, Rossia macrosoma, Loligo vulgaris, Lolliguncula brevis*; Supplementary Figure 2), and is nested with *Abralia veranyi* and *Spirula spirula*, originally belonging to cluster 1, but within the same branching. Species originally included in cluster 5 appear in a different branch mixing with some other members of cluster 1 (see Supplementary Figure 2). Furthermore, octopuses and cuttlefishes mixed together (with the exception of *O. vulgaris*), and with different branching when compared with the maximum-likelihood topology as shown by Lindgren et al. (2012).

## Discussion

Our study indicates that the cephalopod brain is largely differentiated among species (Figure 1 and Table 3; see also: Maddock and Young, 1987; Nixon and Young, 2003) and evolved specific cerebrotypes in disparate taxa (Table 3 and Supplementary Table 5), similar to what has been reported in vertebrates (e.g., Burish et al., 2004; Iwaniuk et al., 2004; Iwaniuk and Hurd, 2005; Yopak, 2012; Kotrschal et al., 2017).

Many comparative studies on brain evolution in vertebrates utilized multivariate statistics (e.g., van Dongen, 1998; Burish et al., 2004; Iwaniuk et al., 2004; Iwaniuk and Hurd, 2005; Lisney and Collin, 2006; Gonzalez-Voyer et al., 2009a; Yopak, 2012; Steinhausen et al., 2016; Kotrschal et al., 2017; Mai and Liao, 2019). The approach is useful to investigate brain evolution for two main reasons: (i) there are contingencies among brain regions that result in correlated evolution among some areas; (ii) a multitude of selection pressures and constraints determine the composition and evolution of the brain.

Our data reflect the view of Maddock and Young (1987) that there are significant quantitative differences between the brains of different cephalopod species, and that - despite individual variations due to growth or other factors (e.g., seasonal), these differences should reflect the habitat the cephalopod occupies (as also suggested in Nixon and Young, 2003). Most of the clusters identify cerebrotypes that map on to ecological and/or behavioral similarities among cephalopod species. By using a hierarchical cluster analysis approach, we recognize 10 groups of species that reveal differences and analogies among the 52 cephalopod species included in our final data set. The topology of the relationship among species we observed (see dendrogram in Figure 3) strongly supports J.Z. Young's view (Young, 1977a) and our working hypothesis that analysis combining relative brain size and life strategies may provide the basis for assumptions on the pressures and adaptations that drove cephalopods to evolve.

Evolutionary speculations are beyond our data and approach. However, to control for phylogenetic dependence/independence of traits here considered—i.e., cephalopod brain diversity—and possibly ruling out bias in detecting relationships we ran phylogenetic principal component analysis (Revell, 2009) thus to explore association between brain proportions of different cephalopods taking into account the phylogenetic relationship between species. Our pPCA benefits from the data of Lindgren et al. (2012). Unfortunately, the currently available phylogenetic data and limited correspondence with detailed “brain data” did not allow us to achieve enough resolution in the phylogeny to be utilized as additional information in this work. The data we present have to be considered a preliminary outcome and the basis of future work.

Octopods are characterized by large brachial and inferior frontal lobes and smaller optic lobes as opposed to decapods (see Figures 1, 2; see also Maddock and Young, 1987). It appears evident that differences in relative proportions of the inferior frontal lobe system and brachial lobes of octopuses, as compared with decapods, are largely linked with the large use of arms associated with the benthic habitat (Young, 1977a; see also Hanlon and Messenger, 2018).

Figure 1 highlights a large variability in the proportions of the lobes within decapods (see also Table 3 for differences between cerebrotypes). This was already noticed by Maddock and Young (1987) and is not surprising considering the numerous families included within the taxon. However, the most striking differences among cephalopod species emerged when considering the vertical lobe system (e.g., about four times differences between species included in cluster 6 vs. cluster 2; Table 3). The five lobules of the vertical lobe in *O. vulgaris*—for example—allow a packing-effect and a volume reduction of the structure by increasing the surface area and the corresponding number of cells counting the lobe (Young, 1963). The small cells (amacrine) also minimize the length of connections, increase connectivity and computational abilities (Young, 1991, 1995; see also Shigeno et al., 2018), and reduce neuropilar space, as occurs in higher vertebrates (e.g., Hofman, 1985; Sherman and Duda, 1999; Hof et al., 2005; Toro and Burnod, 2005; Molnar et al., 2006; Geschwind and Rakic, 2013; Van Essen et al., 2018; Amiez et al., 2019). In *Loligo* (and *Sepia*) we find the opposite: there is no folding of the surface of the vertical lobe, an estimated reduced number of cells, a correspondingly huge neuropil (Young, 1979). In the words of J.Z. Young: “The octopod condition seems to favor a large number of small cells, the decapod a large number of large cells. The large cells are numerous even within the neuropil. These differences are very striking and call for further knowledge of fine structure and experiments on function” (Young, 1979, p. 352; see also for example Shomrat et al., 2011).

Figure 3 presents the outcome of the hierarchical cluster analysis and highlights several common features, for example, between cuttlefish (cluster 2) and shallow-water octopuses (cluster 9), which probably reflect similar adaptations to the environment and/or common mechanisms evolved to counteract predation. Both are reported to have a wide distribution and to have colonized mainly coastal waters of both temperate and tropical regions, although cuttlefish are completely absent from the Americas (but fossil records provide evidence of their existence in those areas). As bottom living organisms, both have had to adapt to various types of substrate (e.g., rubble, rocky reefs, open sand plains) and prey (generalists). The “ecological” demands, in terms of relative habitat complexity and predation pressure, have brought to the evolution of cephalopods in both taxa, development of rich behavioral repertoires, complex cognitive capabilities (for review see for example: Marini et al., 2017; Mather and Dickel, 2017; Hanlon and Messenger, 2018) and reproductive strategies (i.e., intermittent and simultaneous terminal spawning; review in Rocha et al., 2001) capable of dealing with unstable environments. Again as an example, it is not surprising that *O. vulgaris* has the most conspicuous chromatophore lobe (5%) and that *S. officinalis* is characterized by the largest VERT complex (9.7% of total brain size; see Supplementary Information and Figure 1) among the cephalopods included in this study. Following Maddock and Young (1987) “cirrates are sharply distinguished from other octopods by their relatively large brachial lobes and small vertical lobes. […]” and “among the octopods other than cirrates, the benthic species are distinct from the pelagic, largely on a basis of greater brachial and inferior frontal systems. […] epipelagic and bathypelagic [octopods are] broadly separate [… with] the inferior frontal systems […] reduced and the optic lobes […] large. It would not be legitimate to separate the epipelagic from the bathypelagic octopods on the brains alone […] but there are other features that clearly separate the groups such as the arms and web, […] the whole body form and habitat” (Maddock and Young, 1987, p. 765).

The application of a phylogenetic PCA provided an additional interesting approach. We had access to a large dataset (Lindgren et al., 2012), but the convergence in terms of species included was very limited (38 out 188 species corresponding to 20% of species; only 52% of the species of the dataset of this study were retained for pPCA), limiting the potential of this approach.

Our analyses suggest that the phylogenetic signal alone is not a justification for the grouping of species we found (see Figure 3 and Supplementary Figure 2). Due to the limited set of data available to us, we can only hypothesize that brains evolved in cephalopods on the basis of different factors including phylogeny, development and the third factor (life-style adaptations).

Future research will be required extending the dataset by including all different categories of variables here considered and a strong set of phylogenetic signals as recently applied to decapodiforms by Anderson and Lindgren (2021).

In her original study, Borrelli (2007) attempted to correlate cephalopod' relative brain size with species richness (e.g., Lynch, 1990; Owens et al., 1999; Nicolakakis et al., 2003; Sol et al., 2005; Sayol et al., 2019). Species richness, and possibly subspecies (Sol et al., 2005) appears to be affected by behavioral flexibility, so that taxa appearing more flexible to environmental changes (i.e., opportunistic species) are also those that are represented by a higher number of species as opposed to those characterized by specialist species, and which are less speciose. In a preliminary analysis, Borrelli calculated the total number of species per family in the entire class Cephalopoda noting that species counts were differently distributed among the class with some families more speciose. Borrelli was able to obtain mantle length and brain size of 32 species (a single species per family/subfamily was chosen as representative of the taxon) belonging to 28 families (data deduced from Maddock and Young, 1987). Standardized residuals of brain size^8^ were regressed against the log transformed values of the number of species per family/subfamily providing a significant relationship between the two (Linear regression: β = 0.31 ± 0.10, *F*_1,31_ = 8.97, P = 0.005, R^2^ = 0.23; see Figure 2.1 in Borrelli, 2007) that—according to the original study—supports the idea of the behavioral drive hypothesis in cephalopods, *sensu* Wilson (1985): the most speciose cephalopod families were also those having larger brains, as opposed to families with less species. Unfortunately, such an approach was not possible in this work.

During the analysis of the data set and in agreement to what was reported by Borrelli (2007), we faced a problem in attempting to correlate the relative size (volume) of brain areas with body size. A similar issue was also encountered by Maddock and Young (1987) when comparing their data with “previous measurements”; in the words of the Authors “for the optic lobes (which are relatively easy to measure) our figures are about equal to hers [Wirz, 1959, *NdA*] in eight genera, rather more in four genera and less in nine genera. The only serious discrepancies are for *Eledone* and *Bathypolypus* where she records values equivalent to 154 and 94 compared with our 35 and 44. We have checked our figures and, finding no reason to doubt their accuracy, conclude that the differences may be due to differences in the sizes of animals. We intend to undertake a study brain/body sizes in *Eledone* and other cephalopods, which should help to clear up this point” (Maddock and Young, 1987, p. 762-763). The Authors also extend this remark by comparing with data from Frösch (1971) pointing out that the “vertical lobes were considerably smaller in all the newly-hatched forms than in the adults, but the superior frontals and subverticals were larger […]. The subfrontals, like the vertical lobes, were much smaller in the younger animals. Presubably, they develop with learning experience” (Maddock and Young, 1987, p. 764).

The main reason is that “brain scaling” has to be accurately assessed in cephalopod species given the marked variation in the volume of the brain, and of the single lobes within it, with the size and age of the individual (e.g., Packard and Albergoni, 1970; Frösch, 1971; Dickel et al., 1997, 2006; Shigeno et al., 2001a). As a consequence, it is not possible to identify a “reference” or “type” body size at maturity for cephalopods, as it is in many vertebrate species including fish (e.g., Huber et al., 1997), birds (e.g., Portmann, 1947) and mammals (e.g., Stephan and Pirlot, 1970; Marino, 1998). Therefore, we utilized only a-dimensional measurements (i.e., percentages) of the different sections of the brain from our data sources.

It could be extremely interesting and informative in the future to focus attention on a qualitative assessment of the gross brain morphology in cephalopods. This may help in assessing the degree of inter-specific variability in gross brain structures and in finding potential similarities among morphotypes, other than those that result from comparing the typical decapod vs. octopod brain (i.e., *Loligo* or *Octopus*, respectively).

In spite of the wealth of data available from the literature on the organization of the nervous system of cephalopods (e.g., Young, 1971, 1974, 1976, 1977b, 1979; Messenger, 1979; Shigeno et al., 2001a), complete atlases and accurate 3D rendering of the brain morphology are not available (but see Chung et al., 2020). Therefore, a quantitative assessment of gross brain morphology based on the degree of “encephalization” (*sensu lato*), as recently carried out for example in fish (Lisney and Collin, 2006) is still not possible in cephalopods.

An experimental and data-analysis strategy similar to what has been carried out in vertebrates (e.g., Barton, 1996; Clark et al., 2001; Iwaniuk and Hurd, 2005; Kalisinska, 2005; Ratcliffe et al., 2006; Macrì et al., 2019) may reveal important and significant scientific outcomes for cephalopod biology.

## Closing Remarks

Despite the intrinsic limitations of our dataset, the results provide support for a close relationship between “cerebrotypes” and life styles in cephalopods. This, again, supports our working hypothesis that this taxon evolved different sensory (and computational) strategies to cope with the demands of life in the ocean (Amodio et al., 2019a,b). These resemble similar adaptations achieved by fish (e.g., Lisney and Collin, 2006) and other vertebrates. By sharing the same environments and ecological niches, octopuses, squids and their allies were forced to compete with fish (their primary predators), which drove cephalopods to colonize and radiate across the world's oceans (Packard, 1972; O'Dor and Webber, 1986; see also, e.g., Aronson, 1991). Our results strongly support Young's view (1977a) of the evolution of the cephalopod brain.

The evolution of cephalopod cerebrotypes could be related to a number of factors. As mentioned in the introduction, phylogenetic (e.g., closely related species should have a similar brain composition) and developmental (e.g., paralarvae or miniature adults at hatching; see for example: Frösch, 1971; Young and Harman, 1988; Sweeney et al., 1992; Shigeno et al., 2001b) constraints could largely dictate brain composition. In addition, behavior and ecology—as a third factor—appeared to influence cephalopod “cerebrotypes”: species occupying similar niches appear to possess similar brain organization/composition.

Here we attempted to relate the cephalopod cerebrotypes to their “adaptive features” and niches that species occupy. We selected only a number of possible variables to consider, based on the data available for the largest number of species included in this study and our aim: a first attempt to provide a revisited glance to the outcome of observational approach originally driven by Young (1977a). We are fully aware that the other two constraints (phylogenetic and developmental) play an important role in the “evolution” of cerebrotypes in this taxon.

A comparison between the dendrograms (Figure 3 and Supplementary Figure 2) and a phylogenetic tree of the species (see Figure 1 in Lindgren et al., 2012), clearly show that the overall clustering pattern is not congruent with phylogeny to some extent, and suggest that many of the clusters reflect similarities in brain and their relation with behavior and ecology. We found relationships between clustering pattern and behavior and ecology analagous to those found in fish (e.g., Huber et al., 1997), birds (e.g., Iwaniuk and Hurd, 2005) and mammals (e.g., de Winter and Oxnard, 2001).

A future effort should focus on testing the interplay between the above-mentioned factors, with a focus on evolution and phylogeny, thus to test whether the aforementioned constraints are independent or interlinked in the overall evolution of cephalopod brains.

Cephalopod molluscs represent a promising group among invertebrates for studies concerning the organizing principles that underlie the architecture and ontogeny of complex brains. In a similar fashion to multivariate analyses of brain composition in other taxa, our study indicates that the cephalopod brain evolved specific cerebrotypes that have evolved in disparate taxa.

## Data Availability Statement

All the data presented in this study are included in the paper and in the Supplementary Material. Further inquiries can be directed to the corresponding author/s.

## Ethics Statement

Ethical review and approval was not required for the animal study because the study is based on historical data obtained from the literature.

## Author Contributions

LB built the original dataset. GP and MT drafted the manuscript. AT helped with the phylogenetic PCA analysis. All authors contributed to this study and finalized the manuscript.

## Conflict of Interest

The authors declare that the research was conducted in the absence of any commercial or financial relationships that could be construed as a potential conflict of interest.

## Footnotes

^1^Current valid species: *Alloteuthis media, Alloteuthis subulata*.

^2^*Grimalditeuthis* also has flotation devices on its tail; see: http://tolweb.org/Grimalditeuthis_bonplandi/19463.

^3^Current valid species: *Macrotritopus defilippi, Callistoctopus macropus*.

^4^But see: Quetglas et al. (2009); Scheel and Anderson (2012); Villegas et al. (2014).

^5^The species is currently “unaccepted” with accepted name *Bathypolypus bairdii* (see also Table 1).

^6^The diet of these species is defined as ‘specialized’, but not restricted, in our original data source. Recent studies expand knowledge available of habits of deep-sea species (e.g., Quetglas et al., 2001; Valls et al., 2017).

^7^Species retained (in alphabetical order): *Abralia veranyi, Architeuthis dux, Chiroteuthis veranyi veranyi, Cranchia scabra, Eledone cirrhosa, Enteroctopus dofleini, Gonatus fabricii, Grimalditeuthis bonplandi, Illex coindetii, Japetella sp, Loligo vulgaris, Lolliguncula brevis, Octopus vulgaris, Onychoteuthis banksii, Rossia macrosoma, Sepia elegans, Sepia officinalis, Sepiola affinis, Sepiola robusta, Spirula spirula, Taonius pavo, Teuthowenia megalops, Todaropsis eblanae, Tremoctopus violaceus violaceus*.

^8^Factoring out body size. Mantle length and total brain volume were log transformed to normalize variances and consequently regressed (Borrelli, 2007).
