# Supplementary material for: Cerebrotypes in Cephalopods: Brain Diversity and Its Correlation With Species Habits, Life History, and Physiological Adaptations
Source: Front Neuroanat. 2021 Feb 2;14:565109. doi: 10.3389/fnana.2020.565109 (PMC7884766; doi:10.3389/fnana.2020.565109)
Supplement: Supplementary file 1 [file Data_Sheet_1.pdf]

Cerebrotypes in cephalopods: brain diversity and its correlation with species habits, life history and physiological adaptations

Supplementary Information

Giovanna Ponte[1, #], Morag Taite[2,#], Luciana Borrelli[1], Andrea Tarallo[3], A. Louise Allcock[2], Graziano Fiorito[1,*]

[1] Department of Biology and Evolution of Marine Organisms, Stazione Zoologica Anton Dohrn, Villa Comunale, Napoli, Italy

[2] Department of Zoology, Ryan Institute, National University of Ireland Galway, University Road, Galway, Ireland

[3] Department of Research Infrastructures for MArine Biological Resources (RIMAR), Stazione Zoologica Anton Dohrn, Villa Comunale, Napoli, Italy

[#]**Author statement**: These Authors contributed equally to the manuscript

***Correspondence**: Dr. Graziano Fiorito - email: graziano.fiorito@szn.it

Keywords: Neuroecology; cephalopods; Brain diversity; Adaptation

Table of Contents

General organization of cephalopod 'brains' ... 3
Comparative Information ... 5
Supplementary Table 1 – Outline of cephalopod central nervous system ... 8
Pitfalls and workaround ... 13
Building the cephalopod brain data set ... 16
'Functional' brain sets ... 16
Supplementary Table 2 – Functional sets of the cephalopod brain ... 16
Calculation of the relative proportion of 'functional' brain sets ... 17
Cephalopods' life adaptations descriptors ... 18
1. Way of Locomotion ... 19
2. Feeding Habits ... 20
3. 'Development' ... 23
4. Reproduction ... 23
5. Habitat ... 26
Number of species included in the dendrogram and reasons for exclusions ... 27
Supplementary Table 3 – Species-brain and life-adaptation descriptors database ... 31
Supplementary Table 4 – Factor scores after PCA ... 34
Supplementary Table 5 – Differences between 'cerebrotypes' identified by clusters ... 36
Supplementary Table 6 – Correspondence between species included by Lindgren et al. (2012) and this study ... 41
Considerations taken for the phylogenetic PCA and subsequent analysis ... 49
List of References ... 53

General organization of cephalopod 'brains'

J.Z. Young estimated the number of nerve cells to be some 500 million (in an individual of *O. vulgaris* of about 500 g, body weight): nearly half (200 million neurons) form the central nervous system of the animal (Young, 1963). This number appears to be ten thousand times higher than that found in another mollusc, *Aplysia*, and still remains two hundred times higher when compared with the neuronal counts in the brain of the honeybee (*Apis mellifera*). It is rivaling the total number of neurons in higher vertebrates such as the goldcrest and the rat (164 and 200 million neurons, respectively: Olkowicz et al., 2016).

However, it is not only for the large number of neurons that the nervous system of cephalopods is commonly considered the most complex among invertebrates (Young, 1971; Nixon and Young, 2003; see also: Jaaro and Fainzilber, 2006; Katz, 2007; Deryckere and Seuntjens, 2018).

Although somewhat differentiated among the roughly 800 living species, the central nervous system of cephalopods is characterized by a high level of organization so as to be considered by many authors as a "proper" brain, with close affinities to that of vertebrates (e.g., Shigeno et al., 2018). At the same time, it shares the basic molluscan Bauplan[1] with the other members of its class.

As classically illustrated by Pelseneer (1888), the different ganglia of the putative ancestor of molluscs started to fuse together. This was brought about by the shortening of the connectives and commissures that were clustered tightly around the anterior part of the oesophagus, with the cerebral and buccal ganglia mostly arranged above and the remaining ganglia below (for review see also: Bullock, 1965; Budelmann, 1995).

The simplest organization in the central nervous system of cephalopods is that of *Nautilus*. It is characterized by three broad bands, one dorsal (cerebral ganglia and commissure) and two ventral to the oesophagus (pedal anterior and pallioviscera posterior) that are joined laterally (Owen, 1832). It is the lack of lobes protruding from

[1] The typical nervous system of molluscs is characterized by five to six pairs of ganglia (cerebral, buccal, pedal, pleural, parietal and visceral); a member of each pair is connected to the other by a commissure, while connectives allow the link between ganglia of the same side (left or right) of the animal along the antero-posterior axis (for review see Kandel, 1979).

these bands together with the absence of the fusion of the ventral structures in a single suboesophageal mass that are considered primitive features of the cephalopod brain. However, a closer examination of the assemblage of the three bands allows one to identify various differentiated lobes that closely resemble similar structures in coleoids (Young, 1965; Nixon and Young, 2003).

During the course of its evolution, the brain of cephalopods increased its complexity becoming completely surrounded by a cartilaginous capsule in coleoids[2]. It reached the maximum agglomeration of the neural masses by being fused in a supra– and suboesophageal part and two large optic lobes (one on each side)[3], extending laterally from the supraoesophageal mass. This occurred as a result of the addition or loss of ganglia that brought about their change in position and relative volume. It is outside the aims of this work to provide details of the structure of cephalopod nerve cells, lobes and ganglia. A number of monumental works are available from J.Z. Young and coworkers (Young, 1971; 1974; 1976; 1977b; Messenger, 1979; Young, 1979); reviews are provided by Bullock (1965) and Nixon and Young (2003), to cite some. Supplementary Table 1 provides a schematic overview of the major divisions of the “brain” of dibranchiate cephalopods using the terminology adopted by Young and co-workers.

The nervous system of cephalopods shows a series of features that are considered to be unusual to molluscan, and invertebrate or even vertebrate standards (Budelmann, 1995; Hochner et al., 2006). These are: ***i.*** the highest degree of centralization compared with any other mollusc or invertebrate (insects excluded), achieved by the shortening of the connectives; ***ii***. the presence of very small neurons (3-5 micron of nuclear size) acting as local interneurons; ***iii.*** the absence of somatotopy (except for the chromatophore lobes) contrary to what appears to be the case for the insect or vertebrate brain (Plän, 1987; Zullo, 2004); ***iv.*** a blood-brain barrier (an exception for molluscs; Abbott and Pichon, 1987); ***v.*** compound field potentials (similar to those of vertebrate brains); ***vi.*** an elevated efferent innervation of the receptors (e.g., the retina, the equilibrium receptor

[2] In *Nautilus* the cartilaginous capsule is present only on the ventral side of the brain.
[3] Each lobe is placed outside of the cartilaginous box, just behind the eye.

organs); ***vii.*** peripheral first order afferent neurons[4]; ***viii***. a large variety of putative transmitters (review in: Messenger, 1996; Ponte, 2012; Ponte and Fiorito, 2015).

Such a sophisticated central nervous system coupled with a battery of well-developed sense organs is the by-product of cephalopods' life style as voracious marine predators. In the words of J.Z. Young: «It is perhaps even yet not realized what an enormous variety the cephalopods exhibit, inhabiting every part of the ocean, its surface, midwaters and depths, its shores and sea bottoms. Each habitat requires different behaviour. ... By examining the details of the organization of the brain appropriate to each habitat we can draw conclusions about the significance of the patterns of connectivity. … At the same time the brain provides much evidence about evolution» (Young, 1977a, p. 378).

Young and co-workers spent more than three decades collecting and sectioning samples of the brains of as many species of cephalopods as possible with the goal to produce a book describing the central nervous system, sense organs and life history of octopuses, squids, cuttlefish and their allies. Unfortunately, the impressive result of this enterprise only found light after Young's death (Nixon and Young, 2003).

Comparative Information

Nixon and Young's enormous effort (lasting 30 years) to collect and compare the "brains and lives" of cephalopods stimulated interest in this field of research. However, previous studies provided a considerable amount of quantitative data on cephalopod brains (Wirz, 1959; Frösch, 1971; Maddock and Young, 1987).

The three aforementioned publications are somewhat complementary to each other. In her "Étude biométrique du système nerveux des Céphalopodes", Katharina Wirz (1959) was the first to compare quantitative data of the brain of 34 species of cephalopods. Her pioneering study was restricted however to cephalopods of the Mediterranean Sea and, with a few exceptions (juvenile individuals were considered by the Author for: *Chtenopteryx sicula*, *Onychoteuthis banksii*, *Octopoteuthis sicula*, and *Brachioteuthis riisei*), to sub-adult and adult individuals. Frösch (1971) extended Wirz's work by

[4] For example: the equilibrium receptor organs are composed mainly of secondary sensory cells, an unusual configuration even for vertebrate standards. This allows a significant amount of information processing outside the brain centers, i.e. at the level of the receptor epithelia (Budelmann, 1995).

calculating the volumes of the brain lobes in "Schlüpfstadien" (i.e. hatchlings) of ten species of Mediterranean cephalopods. For the species in common with Wirz, Frösch was able to comment about the variations in brain size that occur during growth. Such variations are mainly due to changes in the allometric rate of the different parts of the brain in respect to body (Young, 1963; Packard and Albergoni, 1970; Giuditta et al., 1971), but also due to modifications in the neural "structures" and in the relationship between lobes because of changes in life style with growth (Frösch, 1971). This has been also found in the following years by studying species such as *Sepia officinalis* (Messenger, 1973; Dickel et al., 1997; Dickel et al., 2006), *Idiosepius paradoxus* (Yamamoto et al., 2003), *Todarodes pacificus* (Shigeno et al., 2001), *Amphioctopus fangsiao* (at the time of the study reported as *Octopus ocellatus*, Yamazaki et al., 2002). Frösch (1971) further suggested that the changes in the reciprocal organization of the lobes could reflect Haeckel's theory of recapitulation. For example, in octopods the palliovisceral lobe and the higher motor centers are all relatively larger in juveniles than in adults. This, as suggested by Frösch, may be related to the planktonic (early postembryonic) phase of these species (e.g., *Octopus vulgaris*, *Eledone cirrhosa*, *Argonauta argo*). In addition, the larvae of *A. arg*o have an extremely large inferior frontal lobe at the moment of hatching that is enormously reduced in the adult; again a case of a 'structural' recapitulation of an ancestral pattern.

Maddock and Young (1987) assembled the largest data set available on quantitative information of the brain in cephalopods, determining the volumes of the lobes of the brain for 63 cephalopod species. In analogy to Wirz (1959) and Frösch (1971), the values were expressed as percentages of brain volume. Contrary to the two studies, Maddock and Young did not utilize only Mediterranean species. This allowed them to increase the number of families considered, almost tripling the original diversity encompassed by Wirz, and also included several deep-sea forms. Juveniles (or very young individuals) were not considered in their work.

Maddock and Young (1987) depicted the brain of cephalopods as having: ***i.*** distinct degrees of complexity between species that ***ii.*** parallel the richness in behavior, and ***iii.*** the variety of environments and niches occupied within the marine realm (for review see also Nixon and Young, 2003).

For example, their comparative overview of the quantitative measurements of the lobes revealed that benthic forms of Octopodidae differ from pelagic ones whereby brachial and inferior frontal lobes appear smaller in the latter.

Despite minor differences, the three data sets can be considered as a unicum since the quantitative measurements they provide are deduced by applying similar methods. In addition, we assume that the criteria to assess the limits and attributions of the lobes within each histological section were the same for the three studies. Finally, problems due to shrinkage are not pertinent because the relative size of the different lobes is estimated in percentages and because, if shrinkage did occur, one would not expect different parts of the brain to shrink differentially (Maddock and Young, 1987).

Supplementary Table 1 – Outline of cephalopod central nervous system

A schematic outline of the central nervous system of dibranchiate cephalopods with its possible analogies to the ancestral molluscan ganglia, and the main functions ascribed to the major lobes. The set of lobes are listed in the table from anterior to posterior, dorsal to ventral essentially following Young (1971). The optic lobes are here considered as extensions of the supraoesophageal mass and not as separate lobes of the 'proper brain' (as in Wirz, 1959; Frösch, 1971; Maddock and Young, 1987), following the indications provided by Young (1971, p. 443).
For the different lobes we also refer to 'functional sets' following Maddock and Young (1987; see also Supplementary Table 2). The presence/absence of lobes in decapods and octopods has been deduced from the works of Young (1971; 1974; 1976; 1977b; 1979) and also from the information reviewed by Nixon and Young (2003). The origin of the different parts has been attributed following Bullock (1965). Finally, the function/s of the lobes is derived from Young (1971), Wells (1978), Nixon and Young (2003), and as reviewed by Shigeno et al. (2018).

Lobes	Functional sets	Decapods	Octopods	Origin	Function
**I. Supraoesophageal mass**					
Optic lobes	OPTIC	√	√	New ganglia associated with orbit	Visual analysis and learning
Peduncle lobe	PARA	√	√	New ganglia associated with orbit	Part of motor control system, coordinates motor activity, colour changes and ink-ejection
Olfactory lobe	PARA	√	√	New ganglia associated with orbit	Part of motor control system, coordinates motor activity, colour changes and ink-ejection [?]
Optic gland	LATB	√	√	Probably of nervous origin	Endocrine function
Subradular ganglia		√	√	Ancestral buccal and labial ganglia [?]	Control of movement of the buccal apparatus (*i.e.* jaws, radula, palps) [?]
Inferior buccal ganglia = true buccal		√	√	Ancestral buccal and labial ganglia	Contribution to the movement of beak and radula in feeding
Superior buccal lobe = old labial	INFF	√	√	Ancestral buccal and labial ganglia	Motor control of feeding

Lobes	Functional sets	Decapods	Octopods	Origin	Function
**Supraoesophageal mass** – continued					
INFERIOR FRONTAL LOBE SYSTEM					
Lateral inferior frontal lobes	INFF	√	√	Ancestral cerebral ganglion	Closely inter-related lobes which together form a functional system concerned with chemotactile information from arms
Median inferior frontal lobe	INFF		√	Ancestral cerebral ganglion	Closely inter-related lobes which together form a functional system concerned with chemotactile information from arms
Posterior buccal lobes	INFF	√	√	Ancestral cerebral ganglion [?]	Closely inter-related lobes which together form a functional system concerned with chemotactile information from arms
Subfrontal lobes	INFF		√	Ancestral cerebral ganglion	Closely inter-related lobes which together form a functional system concerned with chemotactile information from arms
SUPERIOR FRONTAL-VERTICAL LOBE SYSTEM					
Superior frontal lobe	VERT	√	√	Ancestral cerebral ganglion	Regulation of exploratory and learning behaviour and memory
Median and paired lateral superior frontal lobes			√		
Anterior and posterior superior frontal lobes		√			

Lobes	Functional sets	Decapods	Octopods	Origin	Function
**Supraoesophageal mass** – continued					
SUPERIOR FRONTAL-VERTICAL LOBE SYSTEM - continued					
Vertical lobe	VERT	√	√	Ancestral cerebral ganglion	Regulation of exploratory and learning behaviour and memory
Subvertical lobe	VERT	√	√	Ancestral cerebral ganglion	Regulation of exploratory and learning behaviour and memory
BASAL LOBE SYSTEM					
Anterior basal lobe	PARA	√	√	Ancestral cerebral ganglion	Control of posture and movement of head and eyes, control of movements related to feeding
Precommissural lobe	VERT	√	√	Ancestral cerebral ganglion	Considered as the main output channel of the vertical lobe system
Dorsal basal lobe	MEDB	√	√	Ancestral cerebral ganglion	Probably controls actions of defence and avoidance
Subpedunculate lobe	MEDB	√	√	Probably of nervous origin	Neurosecretion, sexual maturity [?]
Median basal lobe	MEDB	√	√	Ancestral cerebral ganglion	Concerned in istitution and control of movements of funnel and mantle
Interbasal lobe	MEDB	√	√	Ancestral cerebral ganglion	Dubious (*sensu* Young, 1971)
Lateral basal lobes	LATB	√	√	Ancestral cerebral ganglion	Control of chromatophores and muscle of skin

Lobes	Functional sets	Decapods	Octopods	Origin	Function
**II. Perioesophageal mass**					
Magnocellular lobe	PALL	√	√	Ancestral pleural and visceral ganglia	Intermediate motor centre between supraoesophageal higher and suboesophageal lower motor centres; involved in defence and fast escape reactions. In Decabranchia the giant fibre system originates here
**III. Suboesophageal mass**					
ANTERIOR SUBOESOPHAGEAL MASS = Brachial ganglion					
Prebrachial lobe	BRAC	√	√	New ganglia associated with arms	Actions of arms and suckers
Postbrachial lobe	BRAC	√	√	New ganglia associated with arms	Actions of arms and suckers
MIDDLE SUBOESOPHAGEAL MASS = Pedal ganglion					
Anterior pedal lobe	PEDAL	√	√	Ancestral pedal ganglion	Intermediate and lower motor centres involved in most actions of animal
Anterior chromatophore lobes	CHROM	√	√	Ancestral pedal ganglion	Colour changes of head and arms, textural changes
Lateral pedal lobes	PEDAL	√	√	Ancestral pedal ganglion	Intermediate and lower motor centres involved in most actions of animal
Posterior pedal lobe	PEDAL	√	√	Ancestral pedal ganglion	Intermediate and lower motor centres involved in most actions of animal

Lobes	Functional sets	Decapods	Octopods	Origin	Function
**Suboesophageal mass** – continued					
POSTERIOR SUBOESOPHAGEAL MASS = Palliovisceral ganglion					
Palliovisceral lobe	PALL	√	√	Ancestral pleural and visceral ganglia	Actions of mantle and some of those of the viscera
Posterior chromatophore lobes	CHROM	√	√	Ancestral pleural and visceral ganglia	Colour changes of fins and mantle, textural changes
Fin lobe	FINL	√		Ancestral pleural and visceral ganglia	Movements of fins
Vasomotor lobes			√	Ancestral pleural and visceral ganglia	Control of blood vessels

Pitfalls and workaround

As mentioned in the main text, data from Frösch (1971) are not included in this study since that work was focused only on the brain volumes of hatchlings, thus not comparable to the other two papers (Wirz, 1959; Maddock and Young, 1987), which provided information on mature (?) individuals.

In the two studies, the values of brain size and relative proportions of lobe-areas appear not attributable to a given body size. In fact, neither of the two works provided clear indication of the number of individuals per species utilized in the data sets and from which the brain and body size had been deduced. In more simple terms, was the value assigned to the vertical lobe, for example, derived from a single or 100 individuals of a given species? Maddock and Young (1987) were contradictory on this point; the values of the brain volume and body size included in their data set referred to a single individual although these values were not necessarily «the same as those used in the analyses which were sometimes averaged over several individuals» (Maddock and Young, 1987, p. 741). Nixon and Young (2003) did not help in clearing up the point or in adding information, apart from making a few corrections to the percentages provided for some species.

In Wirz's (1959) data the values of the total volumes of the brain (excluding the optic lobes) and mantle lengths were reported as ranges of the smallest and largest specimen of her sample.

Due to the above, Wirz (1959) and Maddock and Young (1987) data appear to be representative of a given cephalopod species, and any attempt of an estimation of the 'average' brain in reference to a given body size for each species is not possible.

As mentioned in the main text, in cephalopods the volume of the brain (and of the single lobes within it) varies with the size and age of the individual (e.g., Packard and Albergoni, 1970; Frösch, 1971; Dickel et al., 1997; Shigeno et al., 2001; Dickel et al., 2006). In addition, there is general consensus that cephalopods do not present a 'reference' or 'type' body size at maturity, as occurs instead in many vertebrate species such as fish (e.g., Huber et al., 1997; York et al., 2019), birds (e.g., Portmann,

1947), mammals: (e.g., Stephan and Pirlot, 1970; Marino, 1998). The size of individual cephalopods is traditionally indicated as maximum length (or weight; Jereb and Roper, 2005; 2010; Jereb et al., 2016); however, although their growth is assumed to be better described by a slow asymptotic function based on a von Bertalanffy equation (Guerra, 1979; see also Lipinski and Roeleveld, 1990), it is commonly accepted that the observed scattering of points from the predicted function is an index of the variability in the relationship between growth (body size) and age.

In fact, as reviewed by Forsythe and Van Heukelem (1987), a series of biotic (age, size, sex, shape, food, activity, inter- and intra-specific interactions, populational and geographical effects) and abiotic (temperature, light, salinity, water quality) factors appear to affect cephalopods' growth. Among them, temperature and food availability (and quality) appear to be the most important. Changes in temperature are important not only when seasonal changes or latitudinal variations are considered: monthly or even weekly temperature changes may have significant effects on the life history of animals (for review see Forsythe and Van Heukelem, 1987; for details see Forsythe and Hanlon, 1988). Following Dr John W. Forsythe (1993) cephalopod individuals that hatch during periods of warmer seawater temperatures (e.g., as in late spring) produce cohorts that grow faster and become larger than individuals that are born during periods of relatively colder temperatures (as in early spring). At its extreme, hatchlings born at the end of spring could even probably surpass the size of older cohorts that are hatched at the beginning of spring. This hypothesis ("Forsythe Hypothesis" or Forsythe Effect), has been validated during the last years by a series of studies (Arkhipkin, 2004; Moltschaniwskyj, 2004; Pecl et al., 2004; Semmens et al., 2004), providing strong evidence in support of the view that cuttlefish, squid and octopuses, or more generally molluscs (e.g., Lazareth et al., 2006; Killam and Clapham, 2018), are good archives of environmental changes (Richardson, 2001; Forsythe, 2004).

Maddock and Young (1987) could not be aware of this problem. Notwithstanding, the authors noticed some discrepancies when comparing their data with that of Wirz (1959). For example, the volumes of the optic lobes, for the species in common between works, found correspondence for eight but not for 13 genera. Regarding two

genera (*Eledone* and *Bathypolypus*) the authors noticed "serious discrepancies": Wirz calculated the optic lobes to be about five times larger for *Eledone* and slightly more than two times for *Bathypolypus*. In the words of Maddock and Young: «We have checked our figures and, finding no reason to doubt their accuracy, conclude that the differences may be due to differences in the sizes of the animals. We intend to undertake a study of brain/body sizes in *Eledone* and other cephalopods, which should help to clear up this point» (1987, p. 763). We found that: ***i.*** Wirz utilized individuals of *E. moschata* with mantle lengths ranging between 66 and 107 mm and with corresponding volumes of the brain ranging between 65.9 and 121.2 mm^3 (see p. 90 of Wirz, 1959); ***ii.*** Maddock and Young reported the brain volume without listing mantle lengths for all the species of their data set, and referring to their "reference" exemplar with a mantle length of 43.8 mm^2 (see p. 741 of Maddock and Young, 1987). Because of the Forsythe Effect, these differences are not only ascribable to differences in size, but more importantly to differences in age (and maybe population) and to the consequent allometric changes of the cerebral masses during post-hatchling development and growth (for a general review see Gould, 1966; for cephalopods see for example: Packard and Albergoni, 1970; Giuditta et al., 1971), as noticed by Frösch (1971) and as clearly pointed out by Maddock and Young (1987; see also Nixon and Young, 2003).

The study of the evolution of the cephalopod brain and of its problematics due to allometry (for a discussion in other taxa see for example: Clark et al., 2001; Mares et al., 2005) still remains little explored. A renewed effort in this sense should be made in the light of modern approaches.

To circumvent and workaround these problems we utilize only percentages of the different parts of the brain of the cephalopod species included in both papers (Wirz, 1959; Maddock and Young, 1987) with the final aim to search for a potential correspondence between brain size (considered as cerebrotypes) and life adaptations within cephalopods (see also main text).

Building the cephalopod brain data set

In order to merge data provided by the two studies and to construct a species-brain database the following considerations were made.

'Functional' brain sets

Maddock and Young (1987) grouped single brain lobes in functional sets; this resulted to be advantageous for allowing comparisons between species belonging to different taxa within the class. For example, the vertical lobe system (VERT, Supplementary Table 2) includes the superior frontal, vertical, subvertical and precommissural lobes (*sensu* Young, 1971; Maddock and Young, 1987). Wirz (1959), by contrast, did not group the lobes of the brain into functional sets.

We adopted the functional sets approach of Maddock and Young (1987) and determined the correspondence between the two works (Supplementary Table 2) and then the corresponding brain values (see Supplementary Table 3).

Supplementary Table 2 – Functional sets of the cephalopod brain

List of the eight functional sets and corresponding cephalopod brain lobes as compiled for the two data sets (Wirz, 1959; Maddock and Young, 1987). The work of Maddock and Young (1987) is here considered prior to Wirz (1959) since the former authors introduced the concept of "functional sets" to the taxon, to represent the various lobes of the brain in various species of cephalopods.

Functional sets	Maddock and Young, 1987	Wirz, 1959[1]
Supraoesophageal mass		
INFF	Inferior frontal	L. frontal inf.
	Subfrontal	L. sous-frontal
	Superior buccal	L. buccal
	Median inferior frontal	
VERT	Superior frontal	L. frontal sup.[2]
	Vertical	L. vertical[2]
	Subvertical	L. sous-vertical[2]
	Precommissural	
BASAL (=PARA)[3]	Anterior basal	L. basal ant.
	Peduncle	
	Olfactory	

Functional sets	Maddock and Young, 1987	Wirz, 1959[1]
***Supraoesophageal mass** - continued*		
BASAL (=MEDB)[3]	Median basal<br>Dorsal basal<br>Interbasal<br>Dorsolateral<br>Subpedunculate	L. basal post.
BASAL (=LATB)[3]	Lateral basal<br>Optic gland[4]	
Suboesophageal mass		
BRAC	Brachial	L. brachial
PEDAL	Pedal	L. pédieux (6)
PALL	Palliovisceral<br>Magnocellular	L. pall.-vis. (3)<br>L. magnocell.
CHRF[5]	Chromatophore[6]<br>Fin	L. chrom. post.[7]
Optic lobes		
OPTIC	Optic	L. optique

1. The numbers in parentheses reported in this column refer to the original footnotes described by the author (Wirz, 1959) and are included herein to explain which lobes we utilized in the construction of this brain data set.
2. Wirz (1959) also lists a further column (Lobes verticaux) that reports the sum of the lobes of the vertical system, probably also including the precommissural lobe.
3. The measurements of the two papers were here combined by summing up the values of the three columns of Table VI of Maddock and Young (1987; see also Table 2.6 of Nixon and Young, 2003) with the "Lobes basaux" of Wirz (1959) to produce a single functional set: BASAL.
4. The optic gland is listed in Table II of Maddock and Young (1987), but is not included in the equivalent list of Nixon and Young (2003).
5. Following Wirz (1959), the values of the chromatophore and fin lobes were summed up together in the same functional set (here called CHRF) and not considered separately as CHROM and FINL (*sensu:* Maddock and Young, 1987; Nixon and Young, 2003).
6. As reported at p. 758 of Maddock and Young (1987), it should be the posterior chromatophore lobe.
7. As reported in Tableau III of Wirz (1959) includes the fin lobe in the column of the posterior chromatophore lobe (L. chrom. post.).

Calculation of the relative proportion of 'functional' brain sets

Both Wirz and Maddock and Young calculated the size of each lobe of the brain (for a given species) as percentage relative to the volume of the whole brain (i.e. the sum of the supra, peri- and suboesophageal masses). The optic lobes were, instead, considered separately and quantified, again as percentage, by comparing their size to

the whole brain (taken this time only as reference). By doing so, Wirz (1959) quantified the optic lobes of the different species to be within a range between 47 and 202% (in the original work, *Eledone moschata* and *Pyroteuthis margaritifera*, respectively). In a similar way, Maddock and Young (1987) measured the optic lobes as ranging between 13 and 610% (*Cirrothauma murrayi* and *Cranchia scabra*, respectively).

We recalculated the values of the different functional sets as proportions relative to the volume of the whole brain, *id est* as the sum of both the masses and optic lobes. In this way, we did not alter the order of magnitude of the data within and between functional sets and provided values that prevented overemphasizing certain variables (e.g., the volume of the optic lobes) in the standardization procedures required by the assumptions of the clustering technique (Everitt, 1993; Everitt et al., 2001).

Moreover, in order to circumvent the intrinsic differences in the brain size values of the two data sets (see 'Pitfalls and workaround' above), we arbitrarily chose the data of Maddock and Young (1987) for species in common between papers instead of calculating the average of the percentages given by the two works, as is done in common practice with other taxa.

Data show a marked diversification of cerebrotypes among cephalopods (see: Figure 1; Table 3; Supplementary Table 3).

Cephalopods' life adaptations descriptors

The variables chosen to correlate life adaptations to the cephalopods cerebrotypes were deduced from the literature following Borrelli (2007). We considered: way of locomotion, feeding habits, development, reproduction (mating/spawning), habitat (i.e. vertical and horizontal distribution). We do not consider common ancestry, molecular phylogeny, and fossil record. A future study may attempt to combine more type of data including cephalopods cerebrotypes.

We are aware that further knowledge has been acquired over the last few years (see for example: Annex 11 in ICES, 2019; Young et al., 2019), but to be consistent with the

original analysis we preferred to largely refer to most of the information included by Borrelli (2007).

A brief description of each variable included in the final cephalopod species-brain database (Supplementary Table 3) and the source of information included is provided below. In the following list, we also indicate the way characters or character states were coded (binary/multistate variables).

1. Way of Locomotion

Buoyancy and fin morphology were considered to describe cephalopods' way of locomotion.

Buoyancy: According to the knowledge available (Clarke et al., 1979; Maddock and Young, 1984; Clarke, 1988; Seibel et al., 2004) three different characters (Buoy_1, Buoy_2, Buoy_3) were coded as follows:

**Buoy_1**	**Buoy_2**	**Buoy_3**
Dense (1)	Dynamic lift (1)	(0)
	Bottom living (2)	(0)
Near-neutral (2)	Vacuum-gas lift (1)	Chambered shell (1)
		Gas chamber (2)
		Swim bladder (3)
	Chemical lift (2)	Ammonium (1)
		Chlorine (2)
		Lipids/methylamines (3)

Numbers in parenthesis refer to coding adopted

For species with dense body tissues the third character (Buoy_3) was scored as 0 (zero). Moreover, the species belonging to the family Sepiolidae were coded as benthic (i.e. bottom living) with the exception of the subfamily Heteroteuthinae (free-swimming) that were coded as having "dynamic lift" (see also Clarke, 1988).

Fin morphology: Two descriptors of fin morphology (Fin_1, Fin_2) were here considered, following several reviews (Roper et al., 1984; Clarke, 1988; see also: Hanlon and Messenger, 1996; Jereb and Roper, 2005; 2010; Jereb et al., 2016;

Hanlon and Messenger, 2018). Fins (Fin_1) were coded as either absent (0) or present (1). The list of scores (numbered from 1 to 9) of the multistate variable describing the morphology of the fins (Fin_2) was arranged on the basis of fins' origin and evolution and following Clarke (1988). In brief, the fringing fins of *Sepia* (coded as 1) are considered the most ancestral while the elongated and flapping fins of loliginids (coded as 9) are the most recent (see Clarke, 1988 for details). When present (Fin_2 = 0 if fins were absent) they could be: fringing (1), paddle-shaped (2), oval (3)[5], secondary (4)[6], circular and mantle long (5), broad and triangular (6), broad and rounded (7), short, rounded and subterminal (8) or, finally, elongated and flapping (9)[7].

2. Feeding Habits

Diet breadth and beak morphology were both taken into account to describe cephalopods' feeding habits.

Diet breadth - As far as diet richness is concerned the PhD Thesis of Dr Borrelli accurately reviewed available source of data available. For the sake of this work, we deliberately avoided to extend original data.

Following Borrelli (2007), up to ten different prey categories resulted from the literature and from the CephBase database that was still fully available at that time (Nixon and Dilly, 1977; Nixon, 1987; Kear, 1992; Lu and Williams, 1994; Lordan et al., 1998; Wood and Day, 2003; 2006). These 'prey categories' (from zooplankton, to molluscs - including cephalopods -, polychaetae worms, crustaceans, chaetognaths, sea urchins, or fish) appear to be consumed by the cephalopod species included in our database. A small number of prey species (e.g., algae, jellyfish) figured only occasionally and were lumped in the general category 'Diet_other taxa'. Since each prey item was scored following a binary coding

---

[5] Members belonging to the families Cycloteuthidae and Mastigoteuthidae typically present oval fins. However, *Discoteuthis* sp. (belonging to the former family) and *Idioteuthis hjorti* (belonging to the latter one) are exceptions in that the fins are circular and extend the entire length of the mantle (Clarke, 1988).

[6] Secondary fins are typical and well developed in the species *Grimalditeuthis bonplandi* (that was once elevated in the separate family Grimalditeuthidae branching from the Chiroteuthidae). It is only present at a larval stage in Chiroteuthis sp., but is lost in the adult (Clarke, 1988).

[7] Loliginids present long, flapping fins with the exception of *Pickfordiateuthis pulchella* that is characterized by short, rounded and subterminal fins and *Sepioteuthis* sp. in which the fins extend along the whole length of the body (Clarke, 1988).

(present/absent), the estimation of the relative diet variety of each cephalopod species was here accomplished by summing up the number of prey items that resulted per species (V_Diet in Supplementary Table 3). Thus, low scores resulted for species that are reported to have a diet restricted to certain prey items (specialists, e.g., *Spirula spirula*) as opposed to more opportunistic species (generalists, e.g., *Sepia officinalis*) that scored higher values.

As clearly stated by Borrelli (2007), it should be noted that this estimation of diet richness may have been biased by research effort, i.e. by the fact that research in cephalopods has been mainly focused on certain species, and only very recently new information has been added to current knowledge. As originally discussed by Dr Borrelli, the bias is linked to large use of the common cuttlefish (*Sepia officinalis* 93), the common octopus (*Octopus vulgaris* 91) and some squids (e.g., *Doryteuthis pealeii* 25) in respect to other species[8]. Whether our knowledge of diet richness of a given cephalopod species in the wild is affected or not by research effort remains to be explored (see also Villanueva et al., 2017).

Beak morphology - The buccal mass of cephalopods is a complex (roughly spherical) muscular "organ" lying just anterior to the brain and located within a sinus at the base of the arms. It is made of a pair of strong chitinous "U-shaped troughs" that are commonly termed "beak" because of their resemblance to that of parrots. The two beaks (upper and lower) are attached to one another by a series of mandibular muscles that allow their opening and closure. When fitted into each other, their arrangement creates an internal buccal cavity that comprises several parts including the radula, another typical molluscan feature (for review see: Nixon, 1998; Messenger and Young, 1999). The general plan and shape of the buccal complex is well conserved through phylogeny as results from the oldest fossil traces dated back to the Carboniferous (Uyeno and Kier, 2005). However, some differences emerge between decapods and octopods. In the words of Clarke and Maddock: «It might be expected that beak shape would be closely related to the

[8] A short outlook at the papers indexed in Zoological Record (Clarivate Analytics) over the last five (5) years confirm Borrelli et al. (2006) and Borrelli (2007) preliminary analysis of research effort. We found for *Sepia officinalis* 93 indexed papers, for *Octopus vulgaris* 91 works, for *Doryteuthis pealeii* 25 studies, 17 papers for *Euprymna scolopes* and only 3 papers for *Sepioteuthis sepioidea*, to mention some.

kind of food eaten, but although little detail of cephalopod food has been published … no great differences in the food are known which would seem likely to account for the differences in lower beak shape. In absence of any clear relationship with function, one would expect their form to reflect evolutionary relationships» (Clarke and Maddock, 1988, p. 123). A systematic analysis between the food eaten by cephalopods and the specific adaptations linked to feeding (i.e. morphology of the upper and lower beaks, and radula) should be carried out. Unfortunately, a large bulk of knowledge is to date only available on the morphology of the lower beak due to the effort of Dr M.R. Clarke who edited the results of a workshop focused on the identification and description of this structure in 40 cephalopod families, thus providing information for most genera. For the aims of this study and following Borrelli (2007) we considered some of the most distinguishing characters concerning lower beak identification as deduced from Dr M.R. Clarke reference work (Clarke, 1986; for review see also Clarke and Maddock, 1988). The following ten characters were included in the database and coded as described below:

- *i.* Rostrum (R_code): very short (1), short (2), medium (3), long (4);
- *ii.* Rostrum aperture (R_width): narrow (1), moderately broad (2), broad (3);
- *iii.* Hood (H_code): narrow (1), broad (2), very broad (3);
- *iv.* Hood curvature (H_curve): flat (1), curved (2);
- *v.* Notch (N_code): absent (0), shallow (1), normal (2), broad (3), deep (4);
- *vi.* Wing-fold presence (W_presence): absent (0), present (1);
- *vii.* Wing-fold (W_code): absent (0), low or very low (1), fairly high (2), high or very high (3), broad (4);
- *viii.* Lateral wall fold/ridge presence (L_presence): absent (0), present (1);
- *ix.* Lateral wall fold/ridge (L_code): absent (0), marked infold (1), slight (2), normal (3), thick (4), roof-like (5), distinct or very distinct (6);
- *x.* Jaw angle (J_code): absent (0), curved (1), obtuse (2), right (3), acute (4), recessed (5).

3. 'Development'

The three variables included in the database were chosen as an index of the ecological adaptability (i.e. flexibility) of each cephalopod taxon. Species were distinguished in those developing via a paralarval stage (Onto_1 = 1) and those having a direct development (Onto_1 = 2); in the latter case (e.g., *Sepia officinalis*), the two other variables (Onto_2 and Onto_3) were both coded as 0. In addition, species whose paralarval stage underwent metamorphosis (e.g., *Architeuthis dux*) were scored as 0 for the third variable. The variables were nested and coded (see below) following the classification of Young and Harman (1988) and Nesis (1995; 2002) although other sources were also taken into consideration (e.g., Hochberg et al., 1992; O'Shea et al., 2006).

**Onto_1**	**Onto_2**	**Onto_3**
Paralarva (1)	No metamorphosis (0)	Development w/o discontinuity (1)
		Shortened development (2)
	Metamorphosis (1)	(0)
Direct (2)	(0)	(0)

Numbers in parenthesis refer to coding adopted

4. Reproduction

Differences in cephalopods' mating and spawning habits were assessed by considering: the presence/absence of the hectocotylus and/or penis, the spermatophore attachment, the size, grouping and 'habit' of the eggs, the reproductive strategy and the spawning pattern. The information compiled regarding the presence/absence of the hectocotylus/penis and the way spermatophores were attached to the female was found in Nesis (1995).

Hectocotylus - Two attributes (Hecto_1, Hecto_2) were necessary to describe this character. The hectocotylus (**Hecto_1**) was either absent (0) or present (1).

Hecto_2 (0 if absent) is utilized to specify the its origin: originating by modification of the ventral arm/s (1), lateral arm/s (2), or dorsal arm/s (3).

Penis (Pen_1): absent (0), present (1).

Spermatophore attachment - Three different states were coded (see below) following Nesis (1995).

Sperm_1	Sperm_2	Sperm_3
Outside body (1)	Buccal membrane (1)	Seminal receptacle (1)
		Ring (2)
	Head (2)	(0)
Inside body (2)	Mantle (3)	Nuchal cartilage (1)
		Mantle cavity or any place (2)
		Pharetra (3)
		Ovary/oviducal gland/s (4)
		Pre-determined sites (5)

Numbers in parenthesis refer to coding adopted

Sperm_3 was scored as 0 for *Vampyroteuthis infernalis*, the only species (of our sample) in which spermatophores are attached to the female's head.

Eggs - we considered:

Eggs' size (Eg_1): Cephalopods' egg size was here considered as eggs' length following the classification proposed by Hochberg et al. (1992). In particular, eggs' size was ranked into three classes: small (0.2-4 mm, coded as '1'), intermediate (3.5-9 mm, coded as '2'), and large (8-21 mm, coded as '3'). The information compiled was derived from several sources: Roper et al. (1984); Hochberg et al. (1992); Lu et al. (1992); Roper and Sweeney (1992); Young and Mangold (1996a); Boletzky (1998).

Eggs' grouping: Following S. von Boletzky (1998), we considered how eggs are arranged and laid (singly or collectively) and coded (Eg_2, Eg_3) according to the following scheme:

Eg_2	Eg_3
Single eggs (1)	Unencapsulated (1)
	Encapsulated, laid singly (2)
	Encapsulated, laid in batches (3)
Clusters (2)	(0)
Collective capsules (3)	(0)

Numbers in parenthesis refer to coding adopted

Eg_3 was scored as 0 for cephalopods that laid eggs in clusters or collective capsules.

Eggs' habit: Finally, the habitat of eggs' deposition (Eg_4, Eg_5) was also considered among the different species[9] of the data set following the reviews of Nesis (1995) and Boletzky (1998):

**Eg_4**	**Eg_5**
Pelagic (1)	At/near surface (1)
	Intermediate layers (2)
	Deep water (3)
Benthic (2)	(0)
Animal body (3)	Female arms (1)
	Shell (2)
	Ovoviviparity (3)

Numbers in parenthesis refer to coding adopted

Eg_5 was scored as 0 for cephalopods that laid their eggs on the bottom.

Reproductive strategy - Reproductive strategies of cephalopod species have been traditionally attributed to the r-K continuum (MacArthur and Wilson, 1967; Pianka, 1970) as discussed by Boletzky (1981); Mangold (1987); Laptikhovsky (2002). Nigmatullin and Laptikhovsky (1994, cited by Nesis, 2002) suggested another framework that has been further expanded by Nesis (1995). The coding adopted in this work follows Nesis (1995). The variable (**Rep_1**) allows to identify six different reproductive strategies as follows:

i. Illex type (1);
ii. Sthenoteuthis type (2);
iii. Octopus type (3);
iv. Gonatus type (4);
v. Opisthoteuthis type (5);
vi. Argonauta type (6).

[9] Recent findings from many sources report of huge floating masses of eggs from several species that float in currents (e.g., Vijai, 2016; Ringvold and Taite, 2018).

Spawning pattern (Spawn_1): We follow Rocha et al. (2001) in distinguishing four different spawning patterns in cephalopods, as indicated below:

i. Continuous spawning (1);

ii. Multiple spawning (2);

iii. Intermittent terminal spawning (3);

iv. Simultaneous terminal spawning (4).

5. Habitat

Vertical distribution - Cephalopods' distribution in the water column was coded (binary coding: 1 present, 0 absent) in the database by considering the classification proposed by Nesis (2003). According to the Author, cephalopods may either live near the coast in inshore waters (neritic species) or away from the coast in open waters (oceanic species). Both neritic and oceanic communities include species that swim in the water column (pelagic) or have contact with the bottom (benthic).

However, following other Authors cephalopods may also be simply distinguished in 'pelagic' and 'benthic' and each domain can be divided by depth in several 'ecological zones' that define the species vertical distribution (Roper et al., 1984; Wood and Day, 2003; Jereb and Roper, 2005; Quetglas et al., 2005; Wood and Day, 2006; Jereb and Roper, 2010; Jereb et al., 2016). Both views are considered here. Following Borrelli (2007) we scored ten different ecological zones (five for each domain) as follows:

Pelagic:

i. Neritic;
ii. Oceanic: Epipelagic (0-100 m);
iii. Oceanic: Mesopelagic (100-1000m);
iv. Oceanic: Bathypelagic (1000-4000m);
v. Oceanic: Abyssopelagic (4000-6000m);

Benthic:

vi. Littoral system;
vii. On continental shelf;
viii. Deep sea system: Bathybenthic (200-4000m);
ix. Deep sea system: Abyssobenthic (4000-6000m);
x. Deep sea system: Hadobenthic (6000-11500m).

Horizontal distribution - We also considered the geographical distribution as a measure of cephalopods relative dispersal and flexibility to adapt to different temperature zones. Data on distribution were derived by Borrelli (2007) from several sources (Nesis, 1987; Young and Mangold, 1996a; b; 1999b; a; Tsuchiya, 2000; Young and Vecchione, 2001; Nesis, 2003), including the Distribution Range feature of available from the CephBase Species Database (Wood and Day, 2003; 2006). The information was arranged in a distribution sheet where each column represented a specific geographical area (Sherman and Duda, 1999; Sea Around Us, 2016; Pauly et al., 2020). Since each geographical region was binary coded the estimation of the relative dispersal of each cephalopod species was deduced by summing up the number of geographical areas from which each species was reported (**V_Geo** in Supplementary Table 3). Fourteen variables were coded for the world's oceans: six (each) for the Atlantic and Pacific Oceans (i.e. Northwest/east, Western/Eastern Central, Southwest/east) and two for the Indian Ocean (i.e. Western and Eastern). Seas, currents, plateau etc. were grouped in five main marine ecoregions: Polar, Temperate Shelf and Seas, Temperate Upwelling, Tropical Upwelling and Tropical Coral. The Agulhas Current, in particular, was listed in the table twice (see Supplementary Table 3) since it grades between two marine ecoregions: the Temperate Upwelling (in the South Temperate Indo-Pacific) and the Tropical Coral (in the Western Indo-Pacific).

Number of species included in the dendrogram and reasons for exclusions

Supplementary Table 3 includes data of 78 cephalopod species belonging to 41 Families and 11 Subfamilies (see Table 1 for reference). The table is aimed at collecting and making fully available for the scientific community the largest available set of data belonging to cephalopods including 'brain' and 'life-adaptation' descriptors.

The database is organized in a table counting 10764 elements: 624 values related to the 'brains' (eight brain functional sets × 78 species); one hundred-thirty rows

(variables and their states) represent descriptors of life-adaptations of the species considered (see 'Cephalopods' life adaptations descriptors' above).

As thoroughly illustrated, our work is based on previous studies. It provides for the first time a representation of brain diversity in cephalopods with the attempt to find a correlation with their life-style adaptations.
Despite some limitations, the database offers a platform to explore new avenues for a comparative analysis in the study of cephalopod adaptations and novelties by focusing on brain organization, life styles and other 'ecological' variables.
An update of its content, based on recent findings and knowledge currently available, is desirable.

Based on the data available to us (see Borrelli, 2007) a few missing data were encountered: 233 'null' values are counted in the final database, corresponding to 2.3% of the whole dataset.

The cluster analysis excludes cases (i.e. species = rows of the matrix) in the occasion of missing (even) a single value in a given row and the following computations eliminate relevant case(s). Solutions are available to fill-in the missing values (imputation) or adopting marginalization, i.e. ignoring the missing data (e.g., Wagstaff, 2004). In addition, strategies have been developed for the search of the most appropriate method for handling missing data (e.g., Wagstaff, 2004; Basagaña et al., 2013; Manly and Wells, 2015; Fiero et al., 2016; Boluki et al., 2019; Hughes et al., 2019).
Despite CLUSTAN and SPSS offer the possibility of missing data estimation and/or prediction, we preferred to not adopt this strategy. Our choice was based on the fact that the data included in the set of variables here considered and the diversity of species represent a large source of variability, that is difficult to control for in cases of imputation of null values.
Thus, our decision was to adopt the most conservative approach and do not use data/species where null values occurred.

As a consequence of the above mentioned 2.3% of missing data, 26 species (out of the total number of cephalopod species included, n = 78) had missing data. This appeared restricted in a limited set (32) of the 130 variables considered as indicators of 'life-style/adaptations.

These species were then excluded from the final cluster analysis resulting in the dendrogram in Figure 3.

Despite the loss in species, the majority of taxa included in the dataset were taken into account in the final analysis. The species excluded belong to 15 Families (out 41 Families considered) corresponding to 19 species: Bathyteuthidae (1), Chtenopterygidae (1), Cycloteuthidae (1), Histioteuthidae (1), Joubiniteuthidae (1), Lycoteuthidae (1), Mastigoteuthidae (1), Neoteuthidae (1), Octopoteuthidae (2), Pyroteuthidae (2), Cirroteuthidae (2), Opisthoteuthidae (2), Argonautidae (1), Alloposidae (1), Ocythoidae (1) [number of species indicated in parenthesis; see Table 1 and Supplementary Table 3].

In the other cases a single species, member of a given Family and/or Subfamily, resulted with missing data and excluded from the analysis.

Based on the data available, the missing species belonging to taxa included in the twenty-six Families retained in the final analysis represent only a very limited set of cases, and we assume have not biased the final outcome. In all instances a single species attributed to a given taxon was excluded because of missing data, leaving other representatives with enough numerosity (see Table 1 and Supplementary Table 3) for the grouping to be considered consistent.

In addition, the diversity of the brain functional sets belonging to these six species (*Neorossia caroli*, *Pickfordiateuthis pulchella*, *Ancistroteuthis lichtensteini*, *Pterygioteuthis giardia*, *Scaeurgus unicirrhus*, *Vitreledonella richardi*) did not result with significant difference to the other species (n = 52; data not shown), within the limitation of the set we have available.

We cannot rule out the fact that the 26 species excluded from the cluster analysis might have provided a different grouping or the identification of additional clusters,

perhaps suggesting that the divergence in cerebrotypes of cephalopods is more marked than the one we can estimate with the data available to us.

However, our analysis suggests that in terms of completeness of species – and possible inter-species variability – the final loss appeared limited and seem to have only partially influenced the final outcome (clusters). Furthermore, the loss in terms of families/subfamilies (and related species) accounts for about 40% of the total estimated species diversity, and we have no other approaches available to fill it, apart from suggesting future studies.

Supplementary Table 3 – Species-brain and life-adaptation descriptors database

In the following pages, the species-brain database is presented in tabular form together with relative brain areas size (proportions), ecology, life history, feeding, reproductive strategies, and distribution provided for each of the 78 cephalopod species included in this study. Data are included for species (N = 52) included in the cluster analysis (cluster grouping is also included: 1-10; see also Figure 3) and for those (N = 26) excluded for lack of information (in light blue following cluster 10).
For each species (rows), we report:
- the percentages of each of the eight functional sets of lobes (see also Figure 1);
- the way of locomotion (buoyancy and fin morphology);
- the feeding habits (diet breadth and beak morphology);
- the development (ontogenesis);
- the reproduction (hectocotylus/penis; spermatophore attachments; size, grouping and habit of the eggs; reproductive strategy/spawning pattern);
- the habitat (vertical and horizontal distribution).

For definitions, describing single characters or states of a given character, and coding (binary or multistate variables) see 'Cephalopods' life adaptations descriptors' in this Supplementary Information. In the table, Northeast Australian Shelf/Great Barrier Reef = Northeast Australian Shelf/GB Reef.

Cluster	Species	Brain data set	INFF	VERT	BASAL	BRAC	PEDAL	PALL	CHRF	OPTIC
1	Spirula spirula		0.959	4.394	9.561	3.465	5.291	6.250	0.835	69.245
1	Heteroteuthis (Heteroteuthis) dispar		0.864	4.521	6.883	2.413	4.369	5.537	0.813	74.600
1	Brachioteuthis riisei		0.556	3.232	14.299	4.490	8.602	8.174	2.141	58.506
1	Abralia (Asteroteuthis) veranyi		0.496	4.838	12.883	3.256	7.398	7.124	1.598	62.406
1	Abraliopsis (Abraliopsis) morisii		0.209	2.559	6.678	2.327	3.769	7.050	0.675	76.733
2	Sepia officinalis		0.580	9.735	8.658	2.527	6.752	10.771	2.196	58.782
2	Sepia elegans		0.319	8.242	11.528	3.004	7.242	8.024	1.964	59.677
2	Sepia orbignyana		0.342	8.781	11.401	4.110	7.705	7.941	1.916	57.806
3	Sepiola rondeletii		1.382	3.245	6.671	3.936	5.679	7.422	1.713	69.952
3	Sepiola affinis		0.464	3.795	14.000	3.088	8.816	9.862	1.816	58.159
3	Sepiola robusta		0.487	3.299	12.803	4.521	9.402	10.423	1.799	57.265
3	Sepietta oweniana		0.483	3.712	13.843	3.992	9.356	9.178	1.809	57.627
3	Rossia macrosoma		0.672	5.681	10.971	6.309	9.799	13.039	2.549	50.980
4	Loligo vulgaris		0.564	5.526	11.368	4.021	8.996	9.415	2.846	57.265
4	**Loligo (Alloteuthis) media**		1.442	5.819	6.579	1.389	4.535	5.059	1.284	73.893
4	Alloteuthis subulata		0.325	5.640	12.038	2.370	6.527	5.959	1.387	65.753
4	Lolliguncula (Lolliguncula) brevis		1.379	6.595	8.458	4.098	6.818	7.824	2.012	62.817
4	Sepioteuthis sepioidea		0.106	4.901	5.538	1.082	3.819	4.350	1.400	78.803
4	Loligo forbesii		0.252	4.727	5.438	2.914	4.314	3.947	1.308	77.100
5	Architeuthis dux		0.863	6.759	9.396	14.094	6.759	8.821	1.246	52.061
5	Illex illecebrosus		0.519	3.684	6.563	2.698	5.499	5.629	0.934	74.475
5	Illex coindetii		0.419	3.864	11.911	5.318	9.521	8.992	2.347	57.627
5	Todarodes sagittatus		0.477	3.796	10.977	4.483	8.095	7.828	1.962	62.383
5	Todaropsis eblanae		0.456	4.050	9.803	5.166	7.919	9.046	2.170	61.390
5	Onychoteuthis banksii		0.150	3.151	7.323	3.421	5.522	8.133	2.281	70.018
6	Chiroteuthis veranii veranii		0.386	2.959	5.833	2.724	4.611	4.353	0.515	78.619
6	Grimalditeuthis bonplandii		0.222	1.923	3.033	1.307	2.576	2.318	0.838	87.784
6	Taonius pavo		0.437	2.534	4.685	3.354	2.370	4.375	0.456	81.790
6	Galiteuthis glacialis		0.247	2.098	4.020	2.327	3.720	5.060	0.159	82.370
6	Teuthowenia megalops		0.416	2.157	5.006	1.247	2.948	6.609	1.128	80.491
6	Sandalops melancholicus		0.408	2.607	5.842	3.423	5.842	12.217	1.005	68.656
7	Cranchia scabra		0.296	2.215	4.796	1.904	1.904	2.328	0.564	85.992
7	Leachia pacifica		0.286	3.222	8.888	3.690	4.574	5.301	0.000	74.038
7	Helicocranchia papillata		0.258	3.245	7.468	1.571	3.734	8.962	0.489	74.272
7	Bathothauma lyromma		0.343	3.511	4.710	2.655	5.588	4.110	0.385	78.698
7	Egea inermis		0.446	4.786	8.294	2.883	4.727	7.580	0.922	70.363
7	Megalocranchia sp.		1.807	3.953	7.492	5.911	7.116	9.413	1.770	62.538
8	Gonatus (Gonatus) fabricii		0.305	2.683	4.004	2.825	4.553	5.142	0.793	79.695
8	Japetella sp.		3.209	3.941	8.092	4.186	6.627	8.092	0.000	65.853
8	**Eledonella** sp.		1.744	3.003	5.586	3.390	6.070	6.264	0.743	73.200
8	Tremoctopus violaceus		0.613	3.874	6.745	2.425	6.856	6.940	0.251	72.297
9	Octopus vulgaris		4.597	6.814	6.382	8.275	15.035	10.168	2.758	45.971
9	Octopus bimaculatus		4.490	8.279	5.472	10.243	7.109	9.869	0.982	53.555
9	Macrotritopus defilippi		2.817	5.078	5.878	7.339	7.826	5.217	0.452	65.391
9	Callistoctopus macropus		4.624	6.301	9.827	11.792	12.081	11.156	1.098	43.121
9	**"Octopus" salutii**		3.493	8.134	7.784	8.034	11.976	8.782	1.148	50.649
9	Enteroctopus dofleini		6.086	5.945	7.219	12.456	13.659	21.798	1.274	31.564
9	Pteroctopus tetracirrhus		3.377	4.132	7.708	5.483	8.780	9.615	0.596	60.310
10	Eledone moschata		6.925	8.488	11.169	15.562	12.658	17.349	1.564	26.284
10	Eledone cirrhosa		4.808	5.648	10.692	9.236	9.368	13.978	1.214	45.055
10	Bathypolypus sponsalis		6.690	7.805	10.523	9.547	18.467	14.774	1.394	30.801
10	**Benthoctopus piscatorum**		7.284	5.174	9.530	12.253	14.432	16.678	1.157	33.492
**Species not included in the final Cluster**										
	Neorossia caroli		1.008	4.582	6.750	2.657	6.261	8.125	1.130	69.487
	Pickfordiateuthis pulchella		0.845	7.186	6.288	1.480	4.306	5.390	0.925	73.580
	Bathyteuthis sp.		0.618	9.175	6.705	3.573	11.160	11.601	1.191	55.977
	Chtenopteryx sicula		0.151	3.132	6.286	1.447	4.472	5.314	0.670	78.527
	Discoteuthis laciniosa		0.223	2.699	4.662	2.811	4.662	4.283	2.922	77.738
	Histioteuthis miranda		0.251	3.870	5.102	3.443	5.705	4.951	1.231	75.446
	Joubiniteuthis portieri		1.714	3.299	11.654	7.326	7.498	9.640	1.542	57.326
	Lycoteuthis lorigera		0.583	3.335	5.465	2.552	3.878	3.516	0.703	79.968
	Mastigoteuthis schmidti		1.059	2.608	9.046	4.279	5.420	16.341	1.874	59.372
	Neoteuthis thielei		0.549	2.557	6.065	1.268	4.628	5.199	0.888	78.846
	Octopoteuthis sicula		0.646	4.602	13.888	5.631	9.699	9.864	4.214	51.456
	Octopoteuthis danae		0.698	3.981	4.964	3.930	4.473	4.990	2.663	74.302
	Pyroteuthis margaritifera		0.181	2.691	5.925	2.329	4.953	5.043	1.447	77.431
	Ancistroteuthis lichtensteinii		0.509	3.502	12.524	4.150	7.431	6.944	2.393	62.547
	Pterygioteuthis giardi		0.310	2.954	6.600	2.216	4.313	6.004	1.358	76.245
	Cirroteuthis sp.		4.542	2.884	10.238	12.689	15.573	20.260	5.984	27.830
	Cirrothauma murrayi		8.873	2.573	10.825	18.012	20.763	21.295	5.679	11.979
	Opisthoteuthis sp.		3.581	1.479	9.731	5.333	7.824	10.977	0.000	61.074
	Grimpoteuthis sp.		6.267	2.291	7.143	8.086	13.140	27.224	3.302	32.547
	Haliphron atlanticus		0.891	2.461	6.090	2.122	3.947	4.944	0.361	79.185
	Amphitretus sp.		2.526	4.442	9.658	5.705	8.883	7.824	1.548	59.413
	Argonauta argo		1.135	3.682	7.272	2.148	8.101	7.824	0.123	69.715
	Scaeurgus unicirrhus		2.377	5.646	7.021	2.972	8.841	8.581	0.520	64.042
	Ocythoe tuberculata		1.358	2.863	5.984	2.974	8.076	14.427	1.028	63.289
	Vitreledonella richardi		1.146	3.411	8.025	3.239	5.675	6.420	0.172	71.912
	Vampyroteuthis infernalis		1.171	5.769	4.996	1.777	3.261	3.344	0.585	79.097

Cluster	Species	'Ecological' data set	Buoyancy	Buoy_1	Buoy_2	Buoy_3	Fin morphology	Fin_1	Fin_2	Diet breadth	Diet_zooplancton + eggs	Diet_pelagic molluscs	Diet_benthic molluscs	Diet_cephalopods	Diet_polychaete worms	Diet_pelagic crustaceans	Diet_benthic crustaceans	Diet_chaetognaths	Diet_echinoderms	Diet_fish	Diet_other taxa	V_Diet	Beak morphology	R_code	R_width	H_code	H_curve	N_code	W_presence	W_code	L_presence	L_code	J_code
1	Spirula spirula			2	1	1		1	8		0	0	0	0	0	1	0	0	0	0	0	1		3	1	1	2	1	0	0	1	5	2
1	Heteroteuthis (Heteroteuthis) dispar			1	1	0		1	7		0	0	0	1	0	1	1	0	0	1	0	4		4	2	2	2	0	1	2	1	5	2
1	Brachioteuthis riisei			1	1	0		1	6		0	0	0	1	0	1	0	0	0	0	0	2		4	1	1	2	1	1	1	1	6	2
1	Abralia (Asteroteuthis) veranyi			1	1	0		1	6		0	0	0	1	0	1	0	0	0	1	0	3		3	1	2	2	1	1	1	1	6	3
1	Abraliopsis (Abraliopsis) morisii			1	1	0		1	6		0	0	0	0	0	1	0	0	0	0	0	1		3	1	2	2	1	1	3	1	6	3
2	Sepia officinalis			2	1	1		1	1		0	1	1	1	1	1	1	0	0	1	1	8		4	3	2	2	1	1	4	0	0	1
2	Sepia elegans			2	1	1		1	1		0	0	0	0	0	1	1	0	0	1	1	4		4	3	2	2	1	1	4	0	0	1
2	Sepia orbignyana			2	1	1		1	1		0	1	1	1	0	1	1	0	0	1	0	6		4	3	2	2	1	1	4	0	0	1
3	Sepiola rondeletii			1	2	0		1	7		0	0	0	0	0	1	0	0	0	0	0	1		4	2	2	2	0	1	2	0	0	2
3	Sepiola affinis			1	2	0		1	7		0	0	0	0	0	1	0	0	0	0	0	1		4	2	2	2	0	1	2	0	0	2
3	Sepiola robusta			1	2	0		1	7		0	0	0	0	0	1	0	0	0	0	0	1		4	2	2	2	0	1	2	0	0	2
3	Sepietta oweniana			1	2	0		1	7		0	0	0	0	0	1	0	0	0	0	0	1		4	2	2	2	0	1	1	0	0	2
3	Rossia macrosoma			1	2	0		1	7		0	0	0	1	0	1	1	0	0	1	0	4		4	2	2	2	0	1	1	0	0	2
4	Loligo vulgaris			1	1	0		1	9		0	0	1	1	1	1	0	0	1	1	0			4	3	2	2	1	1	1	0	0	2
4	**Loligo (Alloteuthis) media**			1	1	0		1	9		0	0	0	1	0	1	0	0	0	1	0	3		4	3	2	2	1	1	1	0	0	2
4	Alloteuthis subulata			1	1	0		1	9		0	0	0	1	0	1	0	0	0	1	0	3		4	3	2	2	1	1	1	0	0	2
4	Lolliguncula (Lolliguncula) brevis			1	1	0		1	9		1	0	0	0	0	0	0	0	0	0	0	1		4	3	2	2	1	1	1	0	0	2
4	Sepioteuthis sepioidea			1	1	0		1	5		0	0	0	0	0	1	0	0	0	1	0	2		4	3	2	2	1	1	1	0	0	2
4	Loligo forbesii			1	1	0		1	9		0	0	0	1	1	1	1	1	0	1	0	6		4	3	2	2	1	1	1	0	0	2
5	Architeuthis dux			2	2	1		1	3		0	0	0	1	0	0	0	0	0	1	0	2		4	3	3	2	4	1	1	0	0	4
5	Illex illecebrosus			1	1	0		1	6		0	0	0	1	0	1	0	0	0	1	0	3		4	1	2	2	2	1	1	1	3	3
5	Illex coindetii			1	1	0		1	6		0	1	0	1	0	1	0	1	0	1	1	6		4	1	2	2	2	1	1	1	3	3
5	Todarodes sagittatus			1	1	0		1	6		0	0	0	1	0	0	0	0	0	1	0	2		4	1	2	2	2	1	1	1	3	3
5	Todaropsis eblanae			1	1	0		1	6		0	1	0	1	0	1	0	0	0	1	0	4		4	1	2	2	2	1	1	1	3	3
5	Onychoteuthis banksii			1	1	0		1	6		0	0	0	1	0	0	0	0	0	1	0	2		4	1	1	2	0	1	1	1	3	3
6	Chiroteuthis veranii veranii			2	2	1		1	4		0	0	0	1	0	1	0	0	0	1	0	3		2	3	2	2	3	1	3	1	6	2
6	Grimalditeuthis bonplandii			2	2	1		1	4		0	0	0	1	0	1	0	0	0	1	0	3		2	3	2	2	3	1	3	1	3	4
6	Taonius pavo			2	2	1		1	8		0	0	0	1	0	1	0	1	0	1	1	5		2	1	1	2	3	1	4	1	6	2
6	Galiteuthis glacialis			2	2	1		1	8		0	0	0	1	0	1	0	1	0	1	1	5		2	1	1	2	3	1	1	1	3	2
6	Teuthowenia megalops			2	2	1		1	8		0	0	0	1	0	1	0	1	0	1	1	5		2	3	2	2	3	1	1	1	3	2
6	Sandalops melancholicus			2	2	1		1	8		0	0	0	1	0	1	0	1	0	1	1	5		3	3	2	2	0	1	3	0	0	5
7	Cranchia scabra			2	2	1		1	8		0	0	0	1	0	1	0	1	0	1	1	5		4	3	2	2	3	1	1	0	0	5
7	Leachia pacifica			2	2	1		1	8		0	0	0	1	0	1	0	1	0	1	1	5		3	3	2	2	0	1	1	0	0	2
7	Helicocranchia papillata			2	2	1		1	8		0	0	0	1	0	1	0	1	0	1	1	5		3	3	2	2	1	1	1	0	0	2
7	Bathothauma lyromma			2	2	1		1	8		0	0	0	1	0	1	0	1	0	1	1	5		2	2	2	2	1	1	1	0	0	4
7	Egea inermis			2	2	1		1	8		0	0	0	1	0	1	0	1	0	1	1	5		2	3	2	2	4	1	1	0	0	3
7	Megalocranchia sp.			2	2	1		1	8		0	0	0	1	0	1	0	1	0	1	1	5		2	3	2	2	4	1	1	1	6	2
8	Gonatus (Gonatus) fabricii			2	2	3		1	3		0	1	0	0	0	1	0	1	0	1	0	4		2	1	2	2	0	0	0	1	6	2
8	Japetella sp.			2	2	2		0	0		0	1	0	0	0	1	0	1	0	1	0	4		1	3	2	1	0	0	0	1	6	0
8	**Eledonella** sp.			2	2	2		0	0		0	0	0	1	0	1	0	0	0	0	0	2		1	3	2	1	0	0	0	1	6	0
8	Tremoctopus violaceus			2	2	2		0	0		0	1	0	0	0	0	0	0	0	1	0	2		1	3	2	2	0	0	0	0	0	0
9	Octopus vulgaris			1	2	0		0	0		0	0	1	1	1	1	1	0	1	1	1	8		2	3	1	2	3	0	0	1	3	2
9	Octopus bimaculatus			1	2	0		0	0		0	0	1	0	0	0	0	0	0	0	0	1		2	3	1	2	3	0	0	1	3	2
9	Macrotritopus defilippi			1	2	0		0	0		0	0	0	0	0	0	1	0	0	0	0	1		2	3	1	2	3	0	0	1	3	2
9	Callistoctopus macropus			1	2	0		0	0		0	0	0	0	0	0	1	0	0	0	0	1		2	3	1	2	3	0	0	1	3	2
9	**"Octopus" salutii**			1	2	0		0	0		0	0	0	0	0	0	1	0	0	0	0	1		2	3	1	2	3	0	0	1	3	2
9	Enteroctopus dofleini			1	2	0		0	0		0	0	1	1	0	1	1	0	0	1	0	5		2	3	1	2	3	0	0	1	3	2
9	Pteroctopus tetracirrhus			1	2	0		0	0		0	0	0	0	0	1	1	0	0	0	0	2		2	3	1	2	3	0	0	1	3	2
10	Eledone moschata			1	2	0		0	0		0	0	0	0	0	1	1	0	0	0	0	2		2	3	2	2	3	0	0	0	0	5
10	Eledone cirrhosa			1	2	0		0	0		1	0	1	1	1	1	1	0	1	1	0	8		2	3	2	2	3	0	0	0	0	5
10	Bathypolypus sponsalis			1	2	0		0	0		0	1	0	0	0	0	0	0	0	0	0	1		1	3	1	2	3	0	0	0	0	4
10	**Benthoctopus piscatorum**			1	2	0		0	0		0	0	0	0	1	0	0	0	1	0	0	2		1	3	1	2	3	0	0	0	0	4
	Neorossia caroli			1	2	0		1	7		0	0	0	1	0	1	1	0	0	1	0	4		4	2	2	#	#	#	#	#	#	#
	Pickfordiateuthis pulchella			1	1	0		1	8		#	#	#	#	#	#	#	#	#	#	#	#		4	3	2	2	0	1	1	0	0	2
	Bathyteuthis sp.			2	2	3		1	8		#	#	#	#	#	#	#	#	#	#	#	#		4	1	2	2	0	1	1	0	0	2
	Chtenopteryx sicula			1	1	0		1	1		#	#	#	#	#	#	#	#	#	#	#	#		3	3	1	2	0	1	1	0	0	2
	Discoteuthis laciniosa			2	2	1		1	5		#	#	#	#	#	#	#	#	#	#	#	#		2	1	2	2	4	1	1	1	6	2
	Histioteuthis miranda			2	2	1		1	8		0	0	0	1	0	1	0	0	0	1	0	3		4	1	2	2	0	1	3	1	6	3
	Joubiniteuthis portieri			2	2	1		1	8		#	#	#	#	#	#	#	#	#	#	#	#		3	3	2	2	0	1	1	1	5	2
	Lycoteuthis lorigera			1	1	0		1	6		0	0	0	0	0	1	0	0	0	1	0	2		3	1	2	2	4	1	1	1	4	3
	Mastigoteuthis schmidti			2	2	1		1	3		1	0	0	0	0	1	0	0	0	0	0	2		2	1	2	2	3	1	3	1	6	2
	Neoteuthis thielei			1	1	0		1	8		#	#	#	#	#	#	#	#	#	#	#	#		3	2	2	2	3	1	3	1	3	4
	Octopoteuthis sicula			2	2	1		1	5		#	#	#	#	#	#	#	#	#	#	#	#		2	3	2	2	4	1	1	1	6	2
	Octopoteuthis danae			2	2	1		1	5		#	#	#	#	#	#	#	#	#	#	#	#		2	3	2	2	4	1	1	1	6	2
	Pyroteuthis margaritifera			1	1	0		1	6		#	#	#	#	#	#	#	#	#	#	#	#		2	1	2	2	1	1	1	0	0	3
	Ancistroteuthis lichtensteinii			1	1	0		1	6		0	0	0	1	0	0	0	0	0	1	0	2		4	#	#	2	#	#	#	#	#	#
	Pterygioteuthis giardi			1	1	0		1	6		#	#	#	#	#	#	#	#	#	#	#	#		2	1	2	2	3	1	1	0	0	3
	Cirroteuthis sp.			2	#	#		1	2		0	0	0	0	0	1	0	0	0	1	0	2		2	3	3	1	0	0	0	0	0	2
	Cirrothauma murrayi			2	#	#		1	2		0	0	0	0	0	1	0	0	0	1	0	2		2	2	3	1	0	0	0	1	1	2
	Opisthoteuthis sp.			2	#	#		1	2		0	0	0	0	0	1	0	0	0	1	0	2		2	3	2	2	0	0	0	0	0	5
	Grimpoteuthis sp.			2	#	#		1	2		0	0	0	0	1	0	1	0	0	0	0	2		2	3	2	2	0	0	0	0	0	3
	Haliphron atlanticus			2	2	2		0	0		0	0	0	1	0	1	0	0	0	0	0	2		4	2	2	1	0	0	0	1	3	4
	Amphitretus sp.			2	2	2		0	0		#	#	#	#	#	#	#	#	#	#	#	#		#	#	#	#	#	#	#	#	#	#
	Argonauta argo			2	1	2		0	0		0	0	0	0	0	1	0	0	0	1	1	3		1	3	1	#	#	#	#	#	#	#
	Scaeurgus unicirrhus			1	2	0		0	0		#	#	#	#	#	#	#	#	#	#	#	#		2	3	1	2	3	0	0	1	3	2
	Ocythoe tuberculata			2	1	3		0	0		0	1	0	0	0	1	0	0	0	1	0	3		2	3	2	2	0	0	0	1	6	2
	Vitreledonella richardi			2	2	2		0	0		#	#	#	#	#	#	#	#	#	#	#	#		1	3	2	2	0	0	0	1	6	4
	Vampyroteuthis infernalis			2	#	#		1	2		0	0	0	0	0	1	0	0	0	0	1	2		1	3	3	2	0	1	3	1	2	3

Cluster	Species	Ontogenesis	Onto_1	Onto_2	Onto_3	Hectocotylus	Hecto_1	Hecto_2	Penis	Pen_1	Spermatophore attachment	Sperm_1	Sperm_2	Sperm_3	Eggs' size	Eg_1	Eggs' grouping	Eg_2	Eg_3	Eggs' habit	Eg_4	Eg_5	Reproductive strategy	Rep_1	Spawning pattern	Spawn_1
1	Spirula spirula		1	0	2		1	1		0		1	1	1		1		1	3		2	0		1		3
1	Heteroteuthis (Heteroteuthis) dispar		1	0	1		1	3		0		2	3	3		1		1	3		2	0		1		3
1	Brachioteuthis riisei		1	1	0		0	0		1		1	1	2		1		1	1		1	1		2		2
1	Abralia (Asteroteuthis) veranyi		1	1	0		1	1		0		2	3	1		1		1	1		1	1		2		2
1	Abraliopsis (Abraliopsis) morisii		1	1	0		1	1		0		2	3	1		1		1	1		1	1		2		2
2	Sepia officinalis		2	0	0		1	1		0		1	1	1		3		1	3		2	0		1		3
2	Sepia elegans		2	0	0		1	1		0		1	1	1		2		1	3		2	0		1		3
2	Sepia orbignyana		2	0	0		1	1		0		1	1	1		2		1	3		2	0		1		3
3	Sepiola rondeletii		2	0	0		1	3		0		2	3	3		2		1	3		2	0		1		3
3	Sepiola affinis		2	0	0		1	3		0		2	3	3		2		1	3		2	0		1		3
3	Sepiola robusta		2	0	0		1	3		0		2	3	3		2		1	3		2	0		1		3
3	Sepietta oweniana		2	0	0		1	3		0		2	3	3		1		1	3		2	0		1		3
3	Rossia macrosoma		2	0	0		1	3		0		2	3	3		2		1	3		2	0		1		3
4	Loligo vulgaris		1	0	1		1	1		0		1	1	1		1		3	0		2	0		1		3
4	**Loligo (Alloteuthis) media**		1	0	1		1	1		0		1	1	1		1		3	0		2	0		1		3
4	Alloteuthis subulata		1	0	1		1	1		0		1	1	1		1		3	0		2	0		1		3
4	Lolliguncula (Lolliguncula) brevis		1	0	1		1	1		0		1	1	1		1		3	0		2	0		1		3
4	Sepioteuthis sepioidea		2	0	0		1	1		0		1	1	1		2		3	0		2	0		1		3
4	Loligo forbesii		1	0	1		1	1		0		1	1	1		1		3	0		2	0		1		3
5	Architeuthis dux		1	1	0		1	1		1		1	1	2		1		3	0		2	0		4		3
5	Illex illecebrosus		1	1	0		1	1		0		2	3	2		1		3	0		2	0		1		3
5	Illex coindetii		1	1	0		1	1		0		2	3	2		1		3	0		2	0		1		3
5	Todarodes sagittatus		1	1	0		1	1		0		1	1	2		1		3	0		2	0		1		3
5	Todaropsis eblanae		1	1	0		1	1		0		1	1	2		1		3	0		2	0		1		3
5	Onychoteuthis banksii		1	1	0		0	0		1		1	3	5		1		3	0		1	2		4		3
6	Chiroteuthis veranii veranii		1	1	0		0	0		1		2	3	4		1		3	0		1	3		4		3
6	Grimalditeuthis bonplandii		1	1	0		0	0		1		2	3	4		1		3	0		1	3		4		3
6	Taonius pavo		1	1	0		0	0		1		1	3	2		1		3	0		1	3		4		3
6	Galiteuthis glacialis		1	1	0		0	0		1		1	3	5		1		3	0		1	3		4		3
6	Teuthowenia megalops		1	1	0		0	0		1		1	3	5		1		3	0		1	3		4		3
6	Sandalops melancholicus		1	1	0		0	0		1		1	3	2		1		3	0		1	3		4		3
7	Cranchia scabra		1	1	0		1	1		0		2	3	2		1		3	0		1	3		4		3
7	Leachia pacifica		1	1	0		1	1		0		1	3	5		1		3	0		1	3		4		3
7	Helicocranchia papillata		1	1	0		0	0		1		1	3	2		1		3	0		1	3		4		3
7	Bathothauma lyromma		1	1	0		0	0		1		1	3	5		1		3	0		1	3		4		3
7	Egea inermis		1	1	0		0	0		1		1	3	2		1		3	0		1	3		4		3
7	Megalocranchia sp.		1	1	0		0	0		1		1	3	2		1		3	0		1	3		4		3
8	Gonatus (Gonatus) fabricii		1	1	0		0	0		1		1	1	2		2		3	0		3	1		4		3
8	Japetella sp.		1	0	2		0	0		0		2	3	4		1		2	0		3	1		3		4
8	**Eledonella** sp.		1	0	2		1	2		0		2	3	4		1		2	0		3	1		3		4
8	Tremoctopus violaceus		1	0	1		1	2		0		2	3	4		1		2	0		3	1		3		4
9	Octopus vulgaris		1	0	1		1	2		0		2	3	4		1		2	0		2	0		3		4
9	Octopus bimaculatus		1	0	1		1	2		0		2	3	4		1		2	0		2	0		3		4
9	Macrotritopus defilippi		1	0	1		1	2		0		2	3	4		1		2	0		2	0		3		4
9	Callistoctopus macropus		1	0	1		1	2		0		2	3	4		1		2	0		2	0		3		4
9	**"Octopus" salutii**		1	0	2		1	2		0		2	3	4		2		2	0		2	0		3		4
9	Enteroctopus dofleini		1	0	2		1	2		0		2	3	4		2		2	0		2	0		3		4
9	Pteroctopus tetracirrhus		1	0	2		1	2		0		2	3	4		2		2	0		2	0		3		4
10	Eledone moschata		2	0	0		1	2		0		2	3	4		3		2	0		2	0		3		4
10	Eledone cirrhosa		1	0	2		1	2		0		2	3	4		2		2	0		2	0		3		4
10	Bathypolypus sponsalis		2	0	0		1	2		0		2	3	4		3		2	0		2	0		3		1
10	**Benthoctopus piscatorum**		2	0	0		1	2		0		2	3	4		3		2	0		2	0		3		1
	Neorossia caroli		2	0	0		1	3		0		2	3	3		2		1	3		2	0		1		3
	Pickfordiateuthis pulchella		1	0	1		1	1		0		1	1	1		1		3	0		2	0		1		3
	Bathyteuthis sp.		1	0	2		0	0		1		#	#	#		1		3	0		1	3		4		3
	Chtenopteryx sicula		1	0	#		0	0		1		#	#	#		1		3	0		1	3		#		#
	Discoteuthis laciniosa		1	0	#		0	0		1		1	3	2		1		3	0		#	#		4		3
	Histioteuthis miranda		1	0	#		0	0		1		1	3	2		1		3	0		#	#		4		3
	Joubiniteuthis portieri		1	1	0		0	0		1		#	#	#		1		3	0		1	#		4		3
	Lycoteuthis lorigera		1	0	#		0	0		1		#	#	#		1		3	0		2	0		#		#
	Mastigoteuthis schmidti		1	1	0		0	0		1		#	#	#		1		3	0		1	3		4		3
	Neoteuthis thielei		1	0	#		0	0		1		#	#	#		1		3	0		1	3		#		#
	Octopoteuthis sicula		1	0	#		0	0		1		1	3	2		1		3	0		#	#		4		3
	Octopoteuthis danae		1	0	#		0	0		1		1	3	2		1		3	0		#	#		4		3
	Pyroteuthis margaritifera		1	1	0		1	1		0		2	3	1		1		3	0		1	2		2		2
	Ancistroteuthis lichtensteinii		1	1	0		0	0		1		#	#	#		1		3	0		2	0		4		3
	Pterygioteuthis giardi		1	1	0		1	1		0		2	3	1		1		3	0		1	2		2		2
	Cirroteuthis sp.		2	0	0		0	0		1		2	3	4		3		1	2		2	0		5		1
	Cirrothauma murrayi		2	0	0		0	0		1		2	3	4		3		1	2		2	0		5		1
	Opisthoteuthis sp.		2	0	0		0	0		1		2	3	4		3		1	2		2	0		5		1
	Grimpoteuthis sp.		2	0	0		0	0		1		2	3	4		3		1	2		2	0		5		1
	Haliphron atlanticus		1	0	#		1	2		0		2	3	4		1		2	0		3	1		3		4
	Amphitretus sp.		1	0	#		1	2		0		2	3	4		2		2	0		#	#		3		4
	Argonauta argo		1	1	0		1	2		0		2	3	4		1		2	0		3	2		6		1
	Scaeurgus unicirrhus		1	0	1		1	2		0		2	3	4		1		2	0		2	0		3		4
	Ocythoe tuberculata		1	0	#		1	2		0		2	3	4		1		2	0		3	3		3		4
	Vitreledonella richardi		1	0	#		1	2		0		2	3	4		2		2	0		3	3		3		4
	Vampyroteuthis infernalis		1	1	0		0	0		1		1	2	0		2		1	1		1	3		1		3

Cluster	Species	Vertical distribution (Nesis)	Neritic	Oceanic	Pelagic	Benthic	Vertical distribution (others)	Pelagic	Benthic	Neritic	Oceanic: Epipelagic	Oceanic: Mesopelagic	Oceanic: Bathypelagic	Oceanic: Abyssopelagic	Littoral system	On continental shelf	Deep sea system: Bathybenthic	Deep sea system: Abyssobenthic	Deep sea system: Hadobenthic
1	Spirula spirula		0	1	1	0		1	0	0	0	1	0	0	0	0	0	0	0
1	Heteroteuthis (Heteroteuthis) dispar		0	1	1	0		1	0	0	0	1	1	0	0	0	0	0	0
1	Brachioteuthis riisei		0	1	1	0		1	0	0	1	1	1	0	0	0	0	0	0
1	Abralia (Asteroteuthis) veranyi		0	1	1	0		1	0	0	1	1	0	0	0	0	0	0	0
1	Abraliopsis (Abraliopsis) morisii		0	1	1	0		1	0	0	1	1	0	0	0	0	0	0	0
2	Sepia officinalis		1	0	0	1		0	1	0	0	0	0	0	1	1	0	0	0
2	Sepia elegans		1	0	0	1		0	1	0	0	0	0	0	1	1	1	0	0
2	Sepia orbignyana		1	0	0	1		0	1	0	0	0	0	0	1	1	1	0	0
3	Sepiola rondeletii		1	0	0	1		1	1	0	0	0	0	0	1	1	1	0	0
3	Sepiola affinis		1	0	0	1		1	1	0	0	0	0	0	1	1	1	0	0
3	Sepiola robusta		1	0	0	1		1	1	0	0	0	0	0	1	1	1	0	0
3	Sepietta oweniana		1	0	0	1		1	1	0	0	0	0	0	1	1	1	0	0
3	Rossia macrosoma		1	0	0	1		1	1	0	0	0	0	0	1	1	1	0	0
4	Loligo vulgaris		1	0	1	0		1	0	1	1	1	0	0	0	0	0	0	0
4	**Loligo (Alloteuthis) media**		1	0	1	0		1	0	1	1	1	0	0	0	0	0	0	0
4	Alloteuthis subulata		1	0	1	0		1	0	1	0	0	0	0	0	0	0	0	0
4	Lolliguncula (Lolliguncula) brevis		1	0	1	0		1	0	1	0	0	0	0	0	0	0	0	0
4	Sepioteuthis sepioidea		1	0	1	0		1	0	1	0	0	0	0	0	0	0	0	0
4	Loligo forbesii		1	0	1	0		1	0	0	0	1	0	0	0	0	0	0	0
5	Architeuthis dux		0	1	1	0		1	0	0	0	1	0	0	0	0	0	0	0
5	Illex illecebrosus		0	1	1	0		1	0	1	1	1	0	0	0	0	0	0	0
5	Illex coindetii		0	1	1	0		1	0	1	1	1	0	0	0	0	0	0	0
5	Todarodes sagittatus		0	1	1	0		1	0	1	1	1	0	0	0	0	0	0	0
5	Todaropsis eblanae		0	1	1	0		1	0	1	1	1	0	0	0	0	0	0	0
5	Onychoteuthis banksii		0	1	1	0		1	0	0	1	1	0	0	0	0	0	0	0
6	Chiroteuthis veranii veranii		0	1	1	0		1	0	0	1	1	1	0	0	0	0	0	0
6	Grimalditeuthis bonplandii		0	1	1	0		1	0	0	0	1	1	0	0	0	0	0	0
6	Taonius pavo		0	1	1	0		1	0	0	1	1	1	0	0	0	0	0	0
6	Galiteuthis glacialis		0	1	1	0		1	0	0	1	1	0	0	0	0	0	0	0
6	Teuthowenia megalops		0	1	1	0		1	0	0	1	1	1	0	0	0	0	0	0
6	Sandalops melancholicus		0	1	1	0		1	0	0	1	1	1	0	0	0	0	0	0
7	Cranchia scabra		0	1	1	0		1	0	0	1	1	0	0	0	0	0	0	0
7	Leachia pacifica		0	1	1	0		1	0	0	1	1	1	0	0	0	0	0	0
7	Helicocranchia papillata		0	1	1	0		1	0	0	1	1	1	0	0	0	0	0	0
7	Bathothauma lyromma		0	1	1	0		1	0	0	1	1	1	0	0	0	0	0	0
7	Egea inermis		0	1	1	0		1	0	0	1	1	1	0	0	0	0	0	0
7	Megalocranchia sp.		0	1	1	0		1	0	0	1	1	1	0	0	0	0	0	0
8	Gonatus (Gonatus) fabricii		0	1	1	0		1	0	0	1	1	0	0	0	0	0	0	0
8	Japetella sp.		0	1	1	0		1	0	0	1	1	1	0	0	0	0	0	0
8	**Eledonella** sp.		0	1	1	0		1	0	0	0	1	1	0	0	0	0	0	0
8	Tremoctopus violaceus		0	1	1	0		1	0	0	1	1	0	0	0	0	0	0	0
9	Octopus vulgaris		1	0	0	1		0	1	0	0	0	0	0	1	1	0	0	0
9	Octopus bimaculatus		1	0	0	1		0	1	0	0	0	0	0	1	1	0	0	0
9	Macrotritopus defilippi		1	0	0	1		0	1	0	0	0	0	0	1	1	0	0	0
9	Callistoctopus macropus		1	0	0	1		0	1	0	0	0	0	0	1	1	1	0	0
9	**"Octopus" salutii**		1	0	0	1		0	1	0	0	0	0	0	0	1	1	0	0
9	Enteroctopus dofleini		1	0	0	1		0	1	0	0	0	0	0	1	1	0	0	0
9	Pteroctopus tetracirrhus		1	0	0	1		0	1	0	0	0	0	0	1	1	1	0	0
10	Eledone moschata		1	0	0	1		0	1	0	0	0	0	0	1	1	1	0	0
10	Eledone cirrhosa		1	0	0	1		0	1	0	0	0	0	0	1	1	1	0	0
10	Bathypolypus sponsalis		0	1	0	1		0	1	0	0	0	0	0	0	0	1	0	0
10	**Benthoctopus piscatorum**		0	1	0	1		0	1	0	0	0	0	0	0	0	1	0	0
	Neorossia caroli		0	1	0	1		1	1	0	0	0	0	0	0	0	1	0	0
	Pickfordiateuthis pulchella		1	0	1	0		1	0	1	0	0	0	0	0	0	0	0	0
	Bathyteuthis sp.		0	1	1	0		1	0	0	0	1	1	1	0	0	0	0	0
	Chtenopteryx sicula		0	1	1	0		1	0	0	1	1	1	0	0	0	0	0	0
	Discoteuthis laciniosa		0	1	1	0		1	0	0	1	1	0	0	0	0	0	0	0
	Histioteuthis miranda		0	1	1	0		1	0	0	1	1	1	0	0	0	0	0	0
	Joubiniteuthis portieri		0	1	1	0		1	0	0	1	1	1	0	0	0	0	0	0
	Lycoteuthis lorigera		0	1	1	0		1	0	0	1	1	1	0	0	0	0	0	0
	Mastigoteuthis schmidti		0	1	1	0		1	0	0	0	1	1	0	0	0	0	0	0
	Neoteuthis thielei		0	1	1	0		1	0	0	1	1	0	0	0	0	0	0	0
	Octopoteuthis sicula		0	1	1	0		1	0	0	1	1	1	0	0	0	0	0	0
	Octopoteuthis danae		0	1	1	0		1	0	0	1	1	1	0	0	0	0	0	0
	Pyroteuthis margaritifera		0	1	1	0		1	0	0	0	1	0	0	0	0	0	0	0
	Ancistroteuthis lichtensteinii		0	1	1	0		1	0	0	1	1	0	0	0	0	0	0	0
	Pterygioteuthis giardi		0	1	1	0		1	0	0	1	1	0	0	0	0	0	0	0
	Cirroteuthis sp.		0	1	1	0		1	0	0	0	0	1	1	0	0	0	0	0
	Cirrothauma murrayi		0	1	1	0		1	0	0	0	0	0	1	0	0	0	0	0
	Opisthoteuthis sp.		0	1	0	1		1	1	0	0	0	0	0	0	1	1	0	0
	Grimpoteuthis sp.		0	1	0	1		1	1	0	0	0	0	0	0	1	1	1	1
	Haliphron atlanticus		0	1	1	0		1	0	0	1	1	0	0	0	0	0	0	0
	Amphitretus sp.		0	1	1	0		1	0	0	0	1	0	0	0	0	0	0	0
	Argonauta argo		0	1	1	0		1	0	0	1	0	0	0	0	0	0	0	0
	Scaeurgus unicirrhus		1	0	0	1		0	1	0	0	0	0	0	0	1	1	0	0
	Ocythoe tuberculata		0	1	1	0		1	0	0	1	1	0	0	0	0	0	0	0
	Vitreledonella richardi		0	1	1	0		1	0	0	1	1	0	0	0	0	0	0	0
	Vampyroteuthis infernalis		0	1	1	0		1	0	0	0	1	1	0	0	0	0	0	0

																Polar										Temperate Shelf and Seas																							Temperate Upwelling							Tropical Upwelling									Tropical Coral																						
Cluster	Species	Horizontal distribution	Atlantic Northeast	Atlantic Western Central	Atlantic Eastern Central	Atlantic Southwest	Atlantic Southeast	Indian Western	Indian Eastern	Pacific Northwest	Pacific Northeast	Pacific Western Central	Pacific Eastern Central	Pacific Southwest	Pacific Southeast	Atlantic Antarctic	Indian Antarctic	Pacific Antarctic	Arctic Ocean	Bering Sea	Greenland Shelf	Iceland Shelf	Norwegian Shelf	Barents Sea	Kara Sea	Mediterranean Sea	Black Sea	North Temperate Atlantic	Newfoundland-Labrador Shelf	Scotian Shelf	Northeast U.S. Continental Shelf	Baltic Sea	Faroe Plateau	North Sea	Celtic-Biscay Shelf	Iberian Sea	North Temperate Indo-Pacific	Oyashio Current	Sea of Japan	Gulf of Alaska	Southern Ocean	South Brazilian Shelf	Patagonian Shelf	Southwest Australian Shelf	Southeast Australian Shelf	East-Central Australian Shelf	New Zealand Shelf	Horizontal distribution - continued	South Temperate Atlantic	Benguela Current	North Temperate Indo-Pacific	California Current	South Temperate Indo-Pacific	Humboldt Current	Agulhas Current	Eastern Tropical Atlantic	Canary Current	Guinea Current	Central Indo-Pacific	Northwest Australian Shelf	West-Central Australian Shelf	Eastern Indo-Pacific	Gulf of California	Pacific Central-American Coast	Western Tropical Atlantic	Southeast U.S. Continental Shelf	Gulf of Mexico	Caribbean Sea	North Brazilian Shelf	East Brazilian Shelf	Western Indo-Pacific	Agulhas Current	Somali Coastal Current	Red Sea	Arabian Sea	Bay of Bengal	Central Indo-Pacific	Kuroshio Current	South China Sea	Gulf of Thailand	Sulu-Celebes Sea	Indonesia Sea	North Australian Shelf	Northeast Australian Shelf/GB Reef	Eastern Indo-Pacific	Insular Pacific-Hawaiian	V_Geo
1	Spirula spirula		0	0	0	0	0	0	0	0	0	1	0	0	0	0	0	0	0	0	0	0	0	0	0	0	0		0	0	0	0	0	0	0	0		0	0	0		0	0	0	0	0	0			0		0		0	0		1	1		0	0		0	0		0	1	1	0	0		0	0	0	0	0		0	0	0	0	1	0	0		0	6
1	Heteroteuthis (Heteroteuthis) dispar		0	1	1	0	0	0	0	0	0	0	0	0	0	0	0	0	0	0	0	0	0	0	0	1	0		0	0	0	0	0	0	1	0		0	0	0		1	0	0	0	0	0			0		0		0	0		1	1		0	0		0	0		0	0	1	1	1		0	0	0	0	0		0	0	0	0	0	0	0		0	10
1	Brachioteuthis riisei		1	1	1	0	0	0	0	0	0	0	0	0	0	0	0	0	0	0	0	0	0	0	0	1	0		0	0	0	0	0	0	0	0		0	0	0		0	0	0	0	0	0			0		0		0	0		0	0		0	0		0	0		0	1	1	0	0		0	0	0	0	0		0	0	0	0	0	0	0		1	8
1	Abralia (Asteroteuthis) veranyi		0	1	0	0	0	0	0	0	0	0	0	0	0	0	0	0	0	0	0	0	0	0	0	1	0		0	0	0	0	0	0	0	0		0	0	0		0	0	0	0	0	0			1		0		0	0		1	1		0	0		0	0		0	1	0	0	0		0	0	0	0	0		0	0	0	0	0	0	0		0	6
1	Abraliopsis (Abraliopsis) morisii		0	1	0	0	0	0	0	0	0	0	0	0	0	0	0	0	0	0	0	0	0	0	0	0	0		0	0	0	0	0	0	0	0		0	0	0		0	0	0	0	0	0			0		0		0	0		0	0		0	0		0	0		0	1	1	0	0		0	0	0	0	0		0	0	0	0	0	0	0		0	3
2	Sepia officinalis		1	0	0	0	0	0	0	0	0	0	0	0	0	0	0	0	0	0	0	0	0	0	0	1	0		0	0	0	1	0	1	1	1		0	0	0		0	0	0	0	0	0			1		0		0	1		1	1		0	0		0	0		0	0	0	0	0		0	0	0	0	0		0	0	0	0	0	0	0		0	10
2	Sepia elegans		1	0	0	0	0	0	0	0	0	0	0	0	0	0	0	0	0	0	0	0	0	0	0	1	0		0	0	0	0	0	1	1	1		0	0	0		0	0	0	0	0	0			1		0		0	0		1	1		0	0		0	0		0	0	0	0	0		0	0	0	0	0		0	0	0	0	0	0	0		0	8
2	Sepia orbignyana		0	0	0	0	0	0	0	0	0	0	0	0	0	0	0	0	0	0	0	0	0	0	0	1	0		0	0	0	0	0	1	1	1		0	0	0		0	0	0	0	0	0			1		0		0	0		1	1		0	0		0	0		0	0	0	0	0		0	0	0	0	0		0	0	0	0	0	0	0		0	7
3	Sepiola rondeletii		0	0	0	0	0	0	0	0	0	0	0	0	0	0	0	0	0	0	0	0	0	0	0	1	0		0	0	0	0	0	1	1	1		0	0	0		0	0	0	0	0	0			0		0		0	0		1	0		0	0		0	0		0	0	0	0	0		0	0	0	0	0		0	0	0	0	0	0	0		0	5
3	Sepiola affinis		0	0	0	0	0	0	0	0	0	0	0	0	0	0	0	0	0	0	0	0	0	0	0	1	0		0	0	0	0	0	0	0	0		0	0	0		0	0	0	0	0	0			0		0		0	0		0	0		0	0		0	0		0	0	0	0	0		0	0	0	0	0		0	0	0	0	0	0	0		0	1
3	Sepiola robusta		0	0	0	0	0	0	0	0	0	0	0	0	0	0	0	0	0	0	0	0	0	0	0	1	0		0	0	0	0	0	0	0	0		0	0	0		0	0	0	0	0	0			0		0		0	0		0	0		0	0		0	0		0	0	0	0	0		0	0	0	0	0		0	0	0	0	0	0	0		0	1
3	Sepietta oweniana		0	0	0	0	0	0	0	0	0	0	0	0	0	0	0	0	0	0	0	0	0	0	0	1	0		0	0	0	0	0	1	1	1		0	0	0		0	0	0	0	0	0			0		0		0	0		1	0		0	0		0	0		0	0	0	0	0		0	0	0	0	0		0	0	0	0	0	0	0		0	5
3	Rossia macrosoma		0	0	0	0	0	0	0	0	0	0	0	0	0	0	0	0	0	0	0	0	0	0	0	1	0		0	0	0	0	0	1	1	1		0	0	0		0	0	0	0	0	0			0		0		0	0		1	0		0	0		0	0		0	0	0	0	0		0	0	0	0	0		0	0	0	0	0	0	0		0	5
4	Loligo vulgaris		0	0	0	0	0	0	0	0	0	0	0	0	0	0	0	0	0	0	0	0	0	0	0	1	0		0	0	0	0	0	1	1	1		0	0	0		0	0	0	0	0	0			1		0		0	0		1	1		0	0		0	0		0	0	0	0	0		0	0	0	0	0		0	0	0	0	0	0	0		0	7
4	**Loligo (Alloteuthis) media**		0	0	0	0	0	0	0	0	0	0	0	0	0	0	0	0	0	0	0	0	0	0	0	1	0		0	0	0	0	0	1	1	1		0	0	0		0	0	0	0	0	0			0		0		0	0		0	0		0	0		0	0		0	0	0	0	0		0	0	0	0	0		0	0	0	0	0	0	0		0	4
4	Alloteuthis subulata		0	0	0	0	0	0	0	0	0	0	0	0	0	0	0	0	0	0	0	0	0	0	0	1	0		0	0	0	0	0	1	1	1		0	0	0		0	0	0	0	0	0			0		0		0	0		1	0		0	0		0	0		0	0	0	0	0		0	0	0	0	0		0	0	0	0	0	0	0		0	5
4	Lolliguncula (Lolliguncula) brevis		0	0	0	0	0	0	0	0	0	0	0	0	0	0	0	0	0	0	0	0	0	0	0	0	0		0	0	0	0	0	0	0	0		0	0	0		0	0	0	0	0	0			0		0		0	0		0	0		0	0		0	0		1	1	1	1	1		0	0	0	0	0		0	0	0	0	0	0	0		0	5
4	Sepioteuthis sepioidea		0	0	0	0	0	0	0	0	0	0	0	0	0	0	0	0	0	0	0	0	0	0	0	0	0		0	0	0	0	0	0	0	0		0	0	0		0	0	0	0	0	0			0		0		0	0		0	0		0	0		0	0		1	0	1	0	0		0	0	0	0	0		0	0	0	0	0	0	0		0	2
4	Loligo forbesii		0	0	0	0	0	0	0	0	0	0	0	0	0	0	0	0	0	0	0	0	0	0	0	1	0		0	0	0	0	0	1	1	1		0	0	0		0	0	0	0	0	0			0		0		0	0		1	0		0	0		0	0		0	0	0	0	0		1	0	1	1	0		0	0	0	0	0	0	0		0	8
5	Architeuthis dux		1	1	1	0	0	0	0	0	0	0	0	0	0	0	0	0	0	0	0	1	1	0	0	0	0		1	1	1	0	1	1	1	0		0	0	0		0	0	0	0	0	0			0		0		0	0		1	0		0	0		0	0		1	0	0	0	0		0	0	0	0	0		0	0	0	0	0	0	0		0	14
5	Illex illecebrosus		0	0	0	0	0	0	0	0	0	0	0	0	0	0	0	0	0	0	1	1	0	0	0	0	0		1	1	1	0	1	0	1	0		0	0	0		0	0	0	0	0	0			0		0		0	0		0	0		0	0		0	0		1	0	0	0	0		0	0	0	0	0		0	0	0	0	0	0	0		0	9
5	Illex coindetii		1	0	1	0	0	0	0	0	0	0	0	0	0	0	0	0	0	0	0	0	0	0	0	1	1		0	0	0	0	1	1	1	1		0	0	0		0	0	0	0	0	0			1		0		0	0		1	1		0	0		0	0		1	1	1	0	0		0	0	0	0	0		0	0	0	0	0	0	0		0	14
5	Todarodes sagittatus		1	0	1	0	1	0	0	0	0	0	0	0	0	0	0	0	1	0	0	1	1	1	1	1	0		0	0	0	1	1	1	1	1		0	0	0		0	0	0	0	0	0			0		0		0	0		1	1		0	0		0	0		0	0	0	0	0		0	0	0	0	0		0	0	0	0	0	0	0		0	16
5	Todaropsis eblanae		0	0	0	0	0	0	0	0	0	0	0	0	0	0	0	0	0	0	0	0	0	0	0	1	0		0	0	0	0	0	1	1	1		0	0	0		0	0	0	0	1	0			1		0		0	0		1	1		1	1		0	0		0	0	0	0	0		0	0	0	0	0		0	0	0	0	0	0	1		0	11
5	Onychoteuthis banksii		1	1	1	1	1	1	1	1	0	1	1	1	1	0	0	0	0	0	0	0	1	1	0	0	0		1	0	0	1	1	1	1	1		0	0	0		1	1	0	0	1	0			1		0		1	1		1	1		1	1		0	1		0	1	1	1	1		1	1	1	1	1		0	1	1	1	1	1	1		1	48
6	Chiroteuthis veranii veranii		1	0	1	0	1	1	1	0	0	0	0	1	1	0	0	0	0	0	0	0	0	0	0	0	0		0	0	0	0	0	0	0	0		0	0	0		0	0	0	0	0	0			0		0		0	0		0	0		0	0		0	0		0	0	0	0	0		0	0	0	0	0		0	0	0	0	0	0	0		0	7
6	Grimalditeuthis bonplandii		0	1	1	0	0	0	0	0	0	0	1	0	0	0	0	0	0	0	0	0	0	0	0	0	0		0	0	0	0	0	0	0	0		0	0	0		0	0	0	0	0	0			0		0		0	0		0	0		0	0		0	0		0	0	0	0	0		0	0	0	0	0		0	0	0	0	0	0	0		1	4
6	Taonius pavo		1	1	1	1	1	1	1	1	1	1	1	1	1	0	0	0	0	0	0	0	1	0	0	0	0		0	0	0	0	0	0	0	0		0	0	0		0	0	0	0	0	0			0		0		0	0		0	0		0	0		0	0		0	0	0	0	0		0	0	0	0	0		0	0	0	0	0	0	0		0	15
6	Galiteuthis glacialis		0	0	0	0	0	0	0	0	0	0	0	0	0	1	1	1	0	0	0	0	0	0	0	0	0		0	0	0	0	0	0	0	0		0	0	0		0	0	0	0	0	0			0		0		0	0		0	0		0	0		0	0		0	0	0	0	0		0	0	0	0	0		0	0	0	0	0	0	0		0	3
6	Teuthowenia megalops		1	0	0	0	0	0	0	0	0	0	0	0	0	0	0	0	0	0	0	1	0	0	0	0	0		0	0	0	0	1	0	0	0		0	0	0		0	0	0	0	0	0			0		0		0	0		0	0		0	0		0	0		0	0	0	0	0		0	0	0	0	0		0	0	0	0	0	0	0		0	4
6	Sandalops melancholicus		0	0	0	0	0	0	0	0	0	0	0	0	0	0	0	0	0	0	0	0	0	0	0	0	0		0	0	0	0	0	0	0	0		0	0	0		0	0	0	0	0	0			0		0		0	0		0	0		0	0		0	0		0	0	0	0	0		0	0	0	0	0		0	0	0	0	0	0	0		1	1
7	Cranchia scabra		0	1	1	1	1	1	1	1	0	1	1	1	1	0	0	0	0	0	0	0	0	0	0	0	0		0	0	0	0	0	0	0	0		0	0	0		0	0	0	0	0	0			0		0		0	0		0	0		0	0		0	0		0	0	0	0	0		0	0	0	0	0		0	0	0	0	0	0	0		0	11
7	Leachia pacifica		0	1	1	1	1	1	1	1	0	1	1	1	1	0	0	0	0	0	0	0	0	0	0	0	0		0	0	0	0	0	0	0	0		0	0	0		0	0	0	0	0	0			0		0		0	0		0	0		0	0		0	0		0	0	0	0	0		0	0	0	0	0		0	0	0	0	0	0	0		0	11
7	Helicocranchia papillata		1	0	0	0	0	0	0	0	0	0	0	0	0	0	0	0	0	0	0	0	0	0	0	0	0		0	0	0	0	0	0	0	0		0	0	0		0	0	0	0	0	0			0		0		0	0		0	0		0	0		0	0		0	0	0	0	0		0	0	0	0	0		0	0	0	0	0	0	0		0	2
7	Bathothauma lyromma		0	1	1	1	1	1	1	1	0	1	1	1	1	0	0	0	0	0	0	0	0	0	0	0	0		0	0	0	0	0	0	0	0		0	0	0		0	0	0	0	0	0			0		0		0	0		0	0		0	0		0	0		0	0	0	0	0		0	0	0	0	0		0	0	0	0	0	0	0		0	11
7	Egea inermis		1	1	0	0	0	0	0	1	0	0	0	0	0	0	0	0	0	0	0	0	0	0	0	0	0		0	0	0	0	0	0	0	0		0	0	0		0	0	0	0	0	0			0		0		0	0		0	0		0	0		0	0		0	1	0	0	0		0	0	0	0	0		1	0	0	0	0	0	0		0	6
7	Megalocranchia sp.		0	0	0	0	0	0	0	0	0	0	0	0	0	0	0	0	0	0	0	0	0	0	0	0	0		0	0	0	0	0	0	0	0		0	0	0		0	0	0	0	0	0			0		0		0	0		0	0		0	0		0	0		0	0	0	0	0		0	0	0	0	0		1	0	0	0	0	0	0		0	1
8	Gonatus (Gonatus) fabricii		1	0	0	0	0	0	0	0	0	0	0	0	0	0	0	0	1	0	1	1	1	1	0	0	0		1	0	0	0	0	0	0	0		0	0	0		0	0	0	0	0	0			0		0		0	0		0	0		0	0		0	0		0	0	0	0	0		0	0	0	0	0		0	0	0	0	0	0	0		0	8
8	Japetella sp.		1	1	1	1	1	1	1	1	0	1	1	1	1	0	0	0	0	0	0	0	0	0	0	0	0		0	0	0	0	0	0	0	0		0	0	0		0	0	0	0	0	0			0		0		0	1		0	0		0	0		0	0		0	1	1	0	0		1	0	0	0	1		0	0	0	0	0	0	0		0	17
8	**Eledonella sp.**		0	1	1	1	1	1	1	1	0	1	1	1	1	0	0	0	0	0	0	0	0	0	0	0	0		0	0	0	0	0	0	0	0		0	0	0		0	0	0	0	0	0			0		0		0	0		0	0		0	0		0	0		0	0	0	0	0		0	0	0	0	0		0	0	0	0	0	0	0		0	11
8	Tremoctopus violaceus		1	1	1	0	0	0	0	0	0	0	0	0	0	0	0	0	0	0	0	0	0	0	0	1	0		0	0	0	0	0	0	0	1		0	0	0		0	0	0	0	0	0			0		0		0	0		0	0		0	0		0	0		0	0	1	0	0		0	0	0	0	0		0	0	0	0	0	0	0		0	7
9	Octopus vulgaris		0	1	1	0	0	1	0	0	0	0	0	0	0	0	0	0	0	0	0	0	0	0	0	1	0		0	0	0	0	0	1	1	1		0	0	0		1	0	0	0	0	0			1		0		0	1		1	1		0	0		0	0		1	1	1	1	1		1	1	1	1	1		0	1	1	0	0	0	0		0	24
9	Octopus bimaculatus		0	0	0	0	0	0	0	0	0	0	0	0	0	0	0	0	0	0	0	0	0	0	0	0	0		0	0	0	0	0	0	0	0		0	0	0		0	0	0	0	0	0			0		1		0	0		0	0		0	0		1	0		0	0	0	0	0		0	0	0	0	0		0	0	0	0	0	0	0		0	2
9	Macrotritopus defilippi		0	0	0	0	0	0	0	0	0	0	0	0	0	0	0	0	0	0	0	0	0	0	0	1	0		0	0	0	0	0	0	0	0		0	0	0		1	0	0	0	0	0			1		0		0	0		1	1		0	0		0	0		1	1	1	1	1		0	0	0	1	1		0	0	0	0	0	0	0		0	12
9	Callistoctopus macropus		0	1	0	0	0	1	1	0	0	0	1	0	0	0	0	0	0	0	0	0	0	0	0	1	0		0	0	0	0	0	0	0	0		0	0	0		0	0	0	0	0	0			0		0		0	0		0	0		1	0		1	0		0	1	1	1	1		1	1	1	1	1		0	1	1	1	1	0	0		1	22
9	"Octopus" salutii		0	0	0	0	0	0	0	0	0	0	0	0	0	0	0	0	0	0	0	0	0	0	0	1	0		0	0	0	0	0	0	0	1		0	0	0		0	0	0	0	0	0			0		0		0	0		0	0		0	0		0	0		0	0	0	0	0		0	0	0	0	0		0	0	0	0	0	0	0		0	2
9	Enteroctopus dofleini		0	0	0	0	0	0	0	0	0	0	0	0	0	0	0	0	0	1	0	0	0	0	0	0	0		0	0	0	0	0	0	0	0		0	1	1		0	0	0	0	0	0			0		1		0	0		0	0		0	0		0	0		0	0	0	0	0		0	0	0	0	0		0	0	0	0	0	0	0		0	4
9	Pteroctopus tetracirrhus		0	0	1	0	0	0	0	0	0	0	0	0	0	0	0	0	0	0	0	0	0	0	0	1	0		0	0	0	0	0	0	0	0		0	0	0		1	1	0	0	0	0			0		0		0	0		1	1		0	0		0	0		1	1	1	1	1		0	0	0	0	0		0	0	0	0	0	0	0		0	12
10	Eledone moschata		0	0	0	0	0	0	0	0	0	0	0	0	0	0	0	0	0	0	0	0	0	0	0	1	0		0	0	0	0	0	0	0	1		0	0	0		0	0	0	0	0	0			0		0		0	0		0	0		0	0		0	0		0	0	0	0	0		0	0	0	0	0		0	0	0	0	0	0	0		0	2
10	Eledone cirrhosa		0	0	0	0	0	0	0	0	0	0	0	0	0	0	0	0	0	0	0	1	1	0	0	1	0		0	0	0	0	1	1	1	1		0	0	0		0	0	0	0	0	0			0		0		0	0		0	0		0	0		0	0		0	0	0	0	0		0	0	0	0	0		0	0	0	0	0	0	0		0	7
10	Bathypolypus sponsalis		0	0	0	0	0	0	0	0	0	0	0	0	0	0	0	0	0	0	0	0	0	0	0	1	0		0	0	0	0	0	0	0	1		0	0	0		0	0	0	0	0	0			0		0		0	0		1	0		0	0		0	0		0	0	0	0	0		0	0	0	0	0		0	0	0	0	0	0	0		0	3
10	**Benthoctopus piscatorum**		0	0	0	0	0	0	0	0	0	0	0	0	0	0	0	0	0	0	1	1	0	0	0	0	0		1	1	1	0	1	0	1	0		0	0	0		0	0	0	0	0	0			0		0		0	0		0	0		0	0		0	0		0	0	0	0	0		0	0	0	0	0		0	0	0	0	0	0	0		0	7
**Species not included in the final Cluster**																																																																																										
	Neorossia caroli		0	0	0	0	0	0	0	0	0	0	0	0	0	0	0	0	0	0	0	0	0	0	0	1	0		0	0	0	0	0	0	0	0		0	0	0		0	0	0	0	0	0			0		0		0	0		1	1		0	0		0	0		0	0	0	0	0		0	0	0	0	0		0	0	0	0	0	0	0		0	3
	Pickfordiateuthis pulchella		0	0	0	0	0	0	0	0	0	0	0	0	0	0	0	0	0	0	0	0	0	0	0	0	0		0	0	0	0	0	0	0	0		0	0	0		0	0	0	0	0	0			0		0		0	0		0	0		0	0		0	0		0	0	1	0	0		0	0	0	0	0		0	0	0	0	0	0	0		0	1
	Bathyteuthis sp.		0	0	0	1	1	0	0	0	0	0	0	0	1	1	0	1	0	0	0	0	0	0	0	0	0		0	0	0	0	0	0	0	0		0	0	0		0	0	0	0	0	0			0		0		0	0		0	0		0	0		0	0		0	0	0	0	0		0	0	0	0	0		0	0	0	0	0	0	0		0	6
	Chtenopteryx sicula		0	0	0	0	0	0	0	0	0	0	0	0	0	0	0	0	0	0	0	0	0	0	0	1	0		0	0	0	0	0	0	0	0		0	0	0		0	0	0	0	0	0			0		0		0	0		0	0		0	0		0	0		0	0	0	0	0		0	0	0	0	0		0	0	0	0	0	0	0		0	1
	Discoteuthis laciniosa		0	1	1	1	1	1	1	1	0	1	1	1	1	0	0	0	0	0	0	0	0	0	0	0	0		0	0	0	0	0	0	0	0		0	0	0		0	0	0	0	0	0			0		0		0	0		0	0		0	0		0	0		0	0	0	0	0		0	0	0	0	0		0	0	0	0	0	0	0		0	11
	Histioteuthis miranda		0	0	0	0	0	1	0	0	0	1	1	1	1	0	0	0	0	0	0	0	0	0	0	0	0		0	0	0	0	0	0	0	0		0	0	0		0	0	0	1	1	1			0		0		0	0		0	0		0	0		0	0		0	0	0	0	0		1	1	0	1	0		0	0	0	0	0	0	0		1	12
	Joubiniteuthis portieri		0	1	1	1	1	0	0	0	0	0	0	0	0	0	0	0	0	0	0	0	0	0	0	0	0		0	0	0	0	0	0	0	0		0	1	0		0	0	0	0	0	0			0		0		0	0		0	0		0	0		0	0		0	0	1	0	0		0	0	0	0	0		1	0	0	0	0	0	0		1	8
	Lycoteuthis lorigera		0	0	0	0	0	1	0	0	0	0	0	1	0	0	0	0	0	0	0	0	0	0	0	0	0		0	0	0	0	0	0	0	0		0	0	0		0	0	0	0	0	1			0		0		0	0		0	0		0	0		0	0		0	0	0	0	0		0	0	0	0	0		0	0	0	0	0	0	0		0	3
	Mastigoteuthis schmidti		1	0	1	0	0	0	0	0	0	0	0	0	0	0	0	0	0	0	0	1	0	0	0	0	0		0	0	0	0	0	0	0	0		0	0	0		0	0	0	0	0	0			0		0		0	0		1	0		0	0		0	0		0	0	0	0	0		0	0	0	0	0		0	0	0	0	0	0	0		0	4
	Neoteuthis thielei		0	1	0	1	0	0	0	0	0	0	0	0	0	0	0	0	0	0	0	0	0	0	0	0	0		0	0	0	0	0	0	0	0		0	0	0		0	0	0	0	0	0			0		0		0	0		0	0		0	0		0	0		0	0	1	0	0		0	0	0	0	0		0	0	0	0	0	0	0		0	3
	Octopoteuthis sicula		0	1	1	1	1	1	1	0	0	1	0	0	0	0	0	0	0	0	0	0	0	0	0	1	0		0	0	0	0	0	0	0	0		0	0	0		0	0	0	0	0	0			0		0		0	0		0	0		0	0		0	0		0	0	0	0	0		0	0	0	0	0		0	0	0	0	0	0	0		0	8
	Octopoteuthis danae		0	1	1	0	0	0	0	0	0	0	0	0	0	0	0	0	0	0	0	0	0	0	0	0	0		0	0	0	0	0	0	0	0		0	0	0		0	0	0	0	0	0			0		0		0	0		0	0		0	0		0	0		0	0	0	0	0		0	0	0	0	0		0	0	0	0	0	0	0		0	2
	Pyroteuthis margaritifera		1	1	1	1	1	1	0	1	0	1	0	0	0	0	0	0	0	0	0	0	0	0	0	1	0		0	0	0	0	0	0	0	0		0	0	0		0	0	0	0	0	1			0		0		0	0		0	0		0	0		0	0		0	0	0	0	0		0	0	0	0	0		1	0	0	0	0	0	0		0	12
	Ancistroteuthis lichtensteinii		0	0	0	0	0	0	0	0	0	1	0	0	0	0	0	0	0	0	0	0	0	0	0	1	0		0	0	0	0	0	0	0	0		0	0	0		0	0	0	0	0	0			1		0		0	0		0	1		0	0		0	0		0	1	0	0	0		0	0	0	0	0		0	0	0	0	0	0	0		0	5
	Pterygioteuthis giardi		1	1	1	0	0	0	0	0	0	0	0	0	1	0	0	0	0	0	0	0	0	0	0	1	0		0	0	0	0	0	0	0	0		0	0	0		1	1	0	1	1	0			1		0		0	0		0	0		0	0		1	0		0	0	1	0	0		0	0	0	0	1		0	0	0	1	0	0	0		0	15
	Cirroteuthis sp.		0	0	0	0	0	0	0	0	0	0	0	0	0	0	0	0	1	0	0	0	0	0	0	0	0		0	0	0	0	0	0	0	0		0	0	0		0	0	0	0	0	0			0		0		0	0		0	0		0	0		0	0		0	0	0	0	0		0	0	0	0	0		0	0	0	0	0	0	0		0	1
	Cirrothauma murrayi		0	1	1	0	0	0	0	0	0	0	0	0	0	0	0	0	0	0	0	0	0	0	0	0	0		0	0	0	0	0	0	0	0		0	0	0		0	0	0	0	0	0			0		0		0	0		0	0		0	0		0	0		0	0	1	0	0		0	0	0	0	0		0	0	0	0	0	0	0		0	3
	Opisthoteuthis sp.		0	0	0	0	0	0	0	0	0	0	0	0	0	0	0	0	0	0	0	0	0	0	0	1	0		1	1	1	0	1	0	1	1		0	0	0		0	0	0	0	0	0			1		0		0	0		1	1		0	0		0	0		1	0	1	0	0		0	0	0	0	0		0	0	0	0	0	0	0		0	12
	Grimpoteuthis sp.		0	0	0	0	0	0	0	0	0	0	0	0	0	0	0	0	0	0	1	1	0	0	0	0	0		0	1	0	0	1	0	1	1		0	0	0		0	0	0	0	0	0			0		0		0	0		1	0		0	0		0	0		0	0	1	0	0		0	0	0	0	0		0	0	0	0	0	0	0		0	7
	Haliphron atlanticus		1	1	1	1	1	1	1	1	0	1	1	1	1	0	0	0	0	0	0	0	0	0	0	0	0		0	0	0	0	0	0	0	0		0	0	0		0	0	0	0	0	0			0		0		0	0		0	0		0	0		0	0		0	0	0	0	0		0	0	0	0	0		0	0	0	0	0	0	0		0	13
	Amphitretus sp.		0	0	0	0	0	1	1	1	0	1	1	1	1	0	0	0	0	0	0	0	0	0	0	0	0		0	0	0	0	0	0	0	0		0	0	0		0	0	0	0	0	0			0		0		0	0		0	0		0	0		0	0		0	0	0	0	0		0	0	0	0	0		0	0	0	0	0	0	0		1	8
	Argonauta argo		1	1	1	1	1	1	1	0	0	1	1	1	1	0	0	0	0	0	0	0	0	0	0	1	0		0	0	1	0	0	0	1	1		0	0	0		0	0	0	0	0	0			1		1		1	1		1	1		1	1		1	0		1	1	1	1	1		1	1	1	1	1		0	1	1	0	1	0	0		1	40
	Scaeurgus unicirrhus		0	0	0	0	0	0	0	0	0	0	0	0	0	0	0	0	0	0	0	0	0	0	0	1	0		0	0	0	0	0	0	0	1		0	0	0		1	0	0	0	0	0			1		0		0	0		1	1		0	0		0	0		1	1	1	1	1		0	0	0	0	0		0	0	0	0	0	0	0		0	11
	Ocythoe tuberculata		1	1	1	1	1	1	1	0	0	1	1	1	1	0	0	0	0	0	0	0	0	0	0	1	0		0	0	1	0	0	0	1	1		0	0	0		1	1	0	0	0	0			1		1		1	1		1	1		1	1		1	0		1	1	1	1	1		1	1	1	1	1		0	1	1	0	1	0	0		1	42
	Vitreledonella richardi		1	1	1	1	1	1	1	0	0	1	1	1	1	0	0	0	0	0	0	0	0	0	0	1	0		0	0	1	0	0	0	1	1		0	0	0		1	1	0	0	0	0			1		1		1	1		1	1		1	1		1	0		1	1	1	1	1		1	1	1	1	1		0	1	1	0	1	0	0		1	42
	Vampyroteuthis infernalis		1	1	1	1	1	1	1	1	0	1	1	1	1	0	0	0	0	1	0	0	0	0	0	0	0		0	0	0	0	0	0	1	1		1	0	1		0	0	1	0	1	1			1		1		1	1		1	1		1	1		0	0		0	1	1	1	1		1	1	0	1	1		1	0	0	1	1	0	1		0	41

Supplementary Table 4 – Factor scores after PCA

Cephalopod species and their factor scores (rounded to two digits), after regression (Gorsuch, 1983), colors (Orders) and symbols (taxa; see Table 1) coding utilized in Figure 2. Symbols for taxa in most cases refer to a given Family; in some cases, and depending on the numerosity, a given symbol is assigned to more than a single Family.

Color	Symbol	Species	Factor_1	Factor_2	Factor_3
	circle	*Spirula spirula*	-0.59	0.12	0.31
	circle	*Sepia officinalis*	-0.57	0.57	2.33
		Sepia elegans	-0.89	1.20	1.99
		Sepia orbignyana	-0.78	1.11	2.27
	Square	*Sepiola rondeleti*	-0.13	-0.17	-0.72
		Sepiola affinis	-0.68	1.87	0.02
		Sepiola robusta	-0.37	1.58	-0.30
		Sepietta oweniana	-0.60	1.82	0.01
		Rossia macrosoma	0.03	1.40	0.45
		Neorossia caroli	-0.27	-0.35	0.06
		Heteroteuthis (Heteroteuthis) dispar	-0.58	-0.52	0.18
	circle	*Loligo vulgaris*	-0.49	1.65	0.42
		Loligo (Alloteuthis) media	-0.61	-0.47	0.67
		Alloteuthis subulata	-1.03	1.09	0.97
		Lolliguncula (Lolliguncula) brevis	-0.31	0.33	0.95
		Sepioteuthis sepioidea	-0.87	-0.45	0.15
		Pickfordiateuthis pulchella	-0.73	-0.66	1.39
		Loligo forbesii	-0.67	-0.57	0.12
	circle	*Bathyteuthis sp.*	0.06	-0.25	2.22
		Chtenopteryx sicula	-0.70	-0.56	-0.48
	circle	*Architeuthis dux*	0.43	0.04	1.35
	circle	*Brachioteuthis riisei*	-0.68	2.04	-0.25
	down-pointing Triangle	*Chiroteuthis veranii veranii*	-0.55	-0.81	-0.51
		Grimalditeuthis bonplandii	-0.69	-1.31	-1.27
	square	*Cranchia scabra*	-0.80	-1.07	-0.94
		Leachia pacifica	-0.67	-0.33	-0.06
		Taonius pavo	-0.50	-1.18	-0.82
		Galiteuthis glacialis	-0.42	-1.37	-1.01
		Helicocranchia papillata	-0.58	-0.36	-0.44
		Bathothauma lyromma	-0.44	-1.12	-0.27
		Sandalops melancholicus	0.06	-0.46	-1.06
		Egea inermis	-0.61	-0.06	0.33
		Megalocranchia sp.	0.23	-0.02	-0.37
		Teuthowenia megalops	-0.55	-0.68	-1.24
	open circle	*Discoteuthis laciniosa*	-0.57	0.08	-1.34
		Abralia (Asteroteuthis) veranyi	-0.85	1.40	0.57

Color	Symbol	Species	Factor_1	Factor_2	Factor_3
		Abraliopsis (Abraliopsis) morisii	-0.56	-0.48	-0.80
		Gonatus (Gonatus) fabricii	-0.36	-1.08	-0.88
		Histioteuthis miranda	-0.42	-0.63	-0.31
		Joubiniteuthis portieri	0.06	0.87	-0.31
		Lycoteuthis lorigera	-0.61	-0.87	-0.39
		Mastigoteuthis schmidti	0.18	0.62	-1.21
		Neoteuthis thielei	-0.61	-0.55	-0.83
		Octopoteuthis sicula	-0.51	2.89	-0.19
		Octopoteuthis danae	-0.42	-0.10	-0.66
	circle	*Illex illecebrosus*	-0.50	-0.44	-0.25
		Illex coindetii	-0.37	1.59	-0.18
		Todarodes sagittatus	-0.49	1.15	-0.17
		Todaropsis eblanae	-0.29	0.96	-0.22
	up-pointing triangle	*Onychoteuthis banksii*	-0.46	0.46	-0.88
		Ancistroteuthis lichtensteini	-0.74	1.70	-0.29
	open square	*Pyroteuthis margaritifera*	-0.60	-0.28	-0.90
		Pterygioteuthis giardi	-0.62	-0.19	-0.75
	circle	*Cirroteuthis sp.*	2.12	2.40	-1.99
		Cirrothauma murrayi	3.77	1.74	-1.98
		Opisthoteuthis sp.	0.65	-0.49	-1.01
		Grimpoteuthis sp.	2.75	0.27	-2.15
	square	*Argonauta argo*	-0.16	-0.64	-0.04
		Haliphron atlanticus	-0.50	-0.88	-0.74
		Tremoctopus violaceus	-0.34	-0.68	0.00
		Ocythoe tuberculate	0.45	-0.48	-0.99
	up-pointing triangle	*Eledone moschata*	2.40	-0.17	1.97
		Eledone cirrhosa	1.21	-0.01	0.69
	down-pointing triangle	*Octopus vulgaris*	1.42	-0.18	0.76
		Octopus bimaculatus	1.19	-1.51	1.76
		"Octopus" salutii	0.83	-0.56	1.93
		Macrotritopus defilippi	0.48	-1.28	0.56
		Callistoctopus macropus	1.42	-0.28	1.14
		Pteroctopus tetracirrhus	0.64	-0.72	0.04
		Scaeurgus unicirrhus	0.17	-0.76	0.76
		Enteroctopus dofleini	2.73	-0.89	0.37
		Amphitretus sp.	0.17	0.29	0.16
		Japetella sp.	0.28	-0.93	0.14
		Eledonella sp.	-0.01	-0.93	-0.62
	square	*Vitreledonella richardi*	-0.35	-0.54	-0.11
		Bathypolypus sponsalis	2.11	-0.08	1.77
		Benthoctopus piscatorum	2.53	-0.60	0.38
	circle	*Vampyroteuthis infernalis*	-0.65	-1.20	0.76

Supplementary Table 5 – Differences between ‘cerebrotypes’ identified by clusters

Kruskal–Wallis one-way analysis of variance and post hoc pairwise comparisons (Dunn, 1964, as adopted in the calculation procedure by SPSS) for the relative proportions of each of the eight brain regions (i.e. brain-functional sets; Supraesophageal mass: INFF, VERT, BASAL; Suboesophageal mass: BRAC, PEDAL, PALL, CHRF; Optic Lobes: OPTIC) for species grouped in ten clusters identified after hierarchical cluster analysis (species N = 52; see also Table 3 and Figure 3).

Supplementary Table 5.1 – Kruskal-Wallis test statistics

	INFF	VERT	BASAL	BRAC	PEDAL	PALL	CHRF	OPTIC
$\chi^2(9)$	31.22	36.93	25.33	31.42	33.38	27.02	30.49	35.28
p	<0.001	<0.001	0.002	<0.001	<0.001	0.001	<0.001	<0.001

In addition to the asymptotic p, we also utilized the Monte Carlo exact module available in SPSS providing a Monte Carlo (MCp) corrected probability and a 95% CI of the MCp (CIMC).
In all instances MCp resulted < 0.001 (data not shown).

Supplementary Table 5.2 – Post hoc pairwise comparisons of distribution of proportions of each of the eight brain regions considered belonging to different species grouped into clusters (Fig. 3), after Kruskal-Wallis tests. Test statistic and p values are reported for each comparison; SE and standardized test statistics are omitted (after Dunn, 1964, as adopted in the calculation procedure by SPSS). The corrected p value is indicated (dark blue) whenever necessary; significance level = 0.05 (significant p values are in boldface).
Symmetrical values of pairwise comparisons are given in *italics*.

Supraoesophageal mass

INFF

Cluster	1	2	3	4	5	6	7	8	9	10
**1**		*7.20*<br>*0.515*	-2.80<br>0.770	*4.86*<br>*0.596*	*4.53*<br>*0.621*	*8.53*<br>*0.352*	*12.86*<br>*0.161*	-5.30<br>0.602	-19.80<br>**0.026**	-25.05<br>**0.014**
**2**	7.20<br>0.515		-10.00<br>0.366	2.33<br>0.826	-2.66<br>0.803	*1.33*<br>*0.901*	*5.66*<br>*0.597*	-12.50<br>0.280	-27.00<br>**0.010**	-32.25<br>**0.005**
**3**	*-2.80*<br>*0.770*	*-10.00*<br>*0.366*		*7.66*<br>*0.403*	*7.33*<br>*0.424*	*11.33*<br>*0.217*	*15.66*<br>*0.086*	-2.50<br>0.806	-17.00<br>0.055	-22.25<br>**0.029**
**4**	4.86<br>0.596	*2.33*<br>*0.826*	7.66<br>0.403		0.33<br>0.970	*3.66*<br>*0.675*	*8.00*<br>*0.361*	-10.16<br>0.299	-24.66<br>**0.003**	-29.91<br>**0.002**
**5**	4.53<br>0.621	*-2.66*<br>*0.803*	7.33<br>0.424	*0.33*<br>*0.970*		*4.00*<br>*0.648*	*8.33*<br>*0.314*	-9.83<br>0.315	-24.33<br>**0.004**	-29.58<br>**0.002**
**6**	8.53<br>0.352	1.33<br>0.901	11.33<br>0.217	3.66<br>0.675	4.00<br>0.648		*4.33*<br>*0.620*	-13.83<br>0.157	-28.33<br>**0.035**	-33.58<br>**0.027**
**7**	12.86<br>0.161	5.66<br>0.597	15.66<br>0.086	8.00<br>0.361	8.33<br>0.314	4.33<br>0.620		-18.16<br>0.063	-32.66<br>**0.005**	-37.92<br>**0.005**

Supplementary Table 5.2 *continued* - INFF

Cluster	1	2	3	4	5	6	7	8	9	10
**8**	*-5.30*<br>*0.602*	*-12.50*<br>*0.280*	*-2.50*<br>*0.806*	*-10.16*<br>*0.299*	*-9.83*<br>*0.315*	*-13.83*<br>*0.157*	*-18.16*<br>*0.063*		-14.50<br>0.127	-19.75<br>0.065
**9**	*-19.80*<br>***0.026***	*-27.00*<br>***0.010***	*-17.00*<br>*0.055*	*-24.66*<br>***0.003***	*-24.33*<br>***0.004***	*-28.33*<br>***0.035***	*-32.66*<br>***0.005***	*-14.50*<br>*0.127*		-5.25<br>0.580
**10**	*-25.05*<br>***0.014***	*-32.25*<br>***0.005***	*-22.25*<br>***0.029***	*-29.91*<br>***0.002***	*-29.58*<br>***0.002***	*-33.58*<br>***0.027***	*-37.92*<br>***0.005***	*-19.75*<br>*0.065*	*-5.25*<br>*0.580*	

VERT

Cluster	1	2	3	4	5	6	7	8	9	10
**1**		-28.73<br>0.009	-0.10<br>0.992	-14.90<br>0.104	-2.06<br>0.822	*14.10*<br>*0.124*	*7.35*<br>*0.423*	*5.35*<br>*0.599*	-19.11<br>**0.031**	-20.65<br>**0.042**
**2**	*-28.73*<br>*0.009*		*28.63*<br>***0.010***	*13.83*<br>*0.197*	*26.66*<br>***0.013***	*42.83*<br>***0.003***	*36.08*<br>***0.034***	*34.06*<br>***0.003***	*9.619*<br>*0.358*	*8.08*<br>*0.485*
**3**	*-0.10*<br>*0.992*	28.63<br>**0.010**		-14.80<br>0.107	-1.96<br>0.830	*14.20*<br>*0.122*	*7.45*<br>*0.417*	*5.45*<br>*0.592*	-19.01<br>**0.032**	-20.55<br>**0.043**
**4**	*-14.90*<br>*0.104*	13.83<br>0.197	*-14.80*<br>*0.107*		*12.83*<br>*0.142*	*29.00*<br>***0.041***	*22.25*<br>***0.011***	*20.25*<br>***0.038***	-4.21<br>0.617	-5.75<br>0.557
**5**	*-2.06*<br>*0.822*	26.66<br>**0.013**	*-1.96*<br>*0.830*	12.83<br>0.142		*16.16*<br>*0.065*	*9.41*<br>*0.282*	*7.41*<br>*0.448*	-17.05<br>**0.043**	-18.58<br>0.057
**6**	14.10<br>0.124	42.83<br>**0.003**	14.20<br>0.122	29.00<br>**0.041**	16.16<br>0.065		-6.75<br>0.440	-8.75<br>0.371	-33.21<br>**0.004**	-34.75<br>**0.017**
**7**	7.35<br>0.423	36.08<br>**0.034**	7.45<br>0.417	22.25<br>**0.011**	9.41<br>0.282	*-6.75*<br>*0.440*		-2.00<br>0.838	-26.46<br>**0.002**	-28.00<br>**0.004**
**8**	5.35<br>0.599	34.06<br>**0.003**	5.45<br>0.592	20.25<br>**0.038**	7.41<br>0.448	*-8.75*<br>*0.371*	*-2.00*<br>*0.838*		-24.46<br>**0.010**	-26.00<br>**0.015**
**9**	*-19.11*<br>***0.031***	9.619<br>0.358	*-19.01*<br>***0.032***	*-4.21*<br>*0.617*	*-17.05*<br>***0.043***	*-33.21*<br>***0.004***	*-26.46*<br>***0.002***	*24.46*<br>***0.010***		-1.54<br>0.872
**10**	*-20.65*<br>***0.042***	8.08<br>0.485	*20.55*<br>***0.043***	*-5.75*<br>*0.557*	*-18.58*<br>*0.057*	*-34.75*<br>***0.017***	*-28.00*<br>***0.004***	*-26.00*<br>***0.015***	*-1.54*<br>*0.872*	

BASAL

Cluster	1	2	3	4	5	6	7	8	9	10
**1**		-4.80<br>0.665	-6.20<br>0.518	*9.36*<br>*0.307*	*2.7*<br>*0.769*	*26.53*<br>***0.004***	*17.03*<br>*0.063*	*19.95*<br>*0.050*	*13.77*<br>*0.121*	-3.05<br>0.764
**2**	*-4.80*<br>*0.665*		-1.40<br>0.899	*15.56*<br>*0.090*	*8.90*<br>*0.332*	*32.73*<br>***0.018***	*23.23*<br>***0.011***	*26.15*<br>***0.010***	*19.97*<br>***0.024***	*3.15*<br>*0.757*
**3**	*-6.20*<br>*0.518*	*-1.40*<br>*0.899*		*14.16*<br>*0.186*	*7.50*<br>*0.484*	*31.33*<br>***0.003***	*21.83*<br>***0.042***	*24.75*<br>***0.032***	*18.57*<br>*0.076*	*1.75*<br>*0.880*
**4**	9.36<br>0.307	14.16<br>0.186	15.56<br>0.090		-6.66<br>0.446	*17.16*<br>*0.050*	*7.66*<br>*0.381*	*10.58*<br>*0.279*	*4.40*<br>*0.601*	-12.41<br>0.204
**5**	2.7<br>0.769	7.50<br>0.484	8.90<br>0.332	*-6.66*<br>*0.446*		*23.83*<br>***0.006***	*14.33*<br>*0.101*	*17.25*<br>*0.078*	*11.07*<br>*0.189*	-5.75<br>0.557
**6**	26.53<br>**0.004**	31.33<br>**0.003**	32.73<br>**0.018**	17.16<br>0.050	23.83<br>**0.006**		-9.50<br>0.278	-6.58<br>0.501	-12.76<br>0.130	-29.58<br>**0.002**
**7**	17.03<br>0.063	21.83<br>**0.042**	23.23<br>**0.011**	7.66<br>0.381	14.33<br>0.101	*-9.50*<br>*0.278*		*2.91*<br>*0.766*	-3.26<br>0.699	-20.06<br>**0.040**
**8**	19.95<br>0.050	24.75<br>**0.032**	26.15<br>**0.010**	10.58<br>0.279	17.25<br>0.078	*-6.58*<br>*0.501*	2.91<br>0.766		-6.18<br>0.515	-23.00<br>**0.032**
**9**	13.77<br>0.121	18.57<br>0.076	19.97<br>**0.024**	4.40<br>0.601	11.07<br>0.189	*-12.76*<br>*0.130*	*-3.26*<br>*0.699*	*-6.18*<br>*0.515*		-16.82<br>0.077
**10**	*-3.05*<br>*0.764*	1.75<br>0.880	3.15<br>0.757	*-12.41*<br>*0.204*	*-5.75*<br>*0.557*	*-29.58*<br>***0.002***	*-20.06*<br>***0.040***	*-23.00*<br>***0.032***	*-16.82*<br>*0.077*	

Supplementary Table 5.2 *continued*

Suboesophageal mass

BRAC

Cluster	1	2	3	4	5	6	7	8	9	10
**1**		-1.10<br>0.921	-10.90<br>0.255	4.40<br>0.632	-13.10<br>0.153	4.98<br>0.587	4.40<br>0.632	-0.85<br>0.933	-24.81<br>**0.005**	-28.10<br>**0.006**
**2**	-1.10<br>0.921		-9.80<br>0.376	5.50<br>0.608	-12.00<br>0.283	6.08<br>0.570	5.50<br>0.608	0.25<br>0.963	-23.71<br>**0.023**	-27.00<br>**0.020**
**3**	-10.90<br>0.255	-9.80<br>0.376		15.30<br>0.095	-2.20<br>0.811	15.88<br>0.083	15.30<br>0.095	10.05<br>0.323	-13.91<br>0.117	-17.20<br>0.091
**4**	4.40<br>0.632	5.50<br>0.608	15.30<br>0.095		-17.50<br>**0.045**	0.58<br>0.947	0.0<br>1.000	-5.25<br>0.591	-29.14<br>0.024	-32.50<br>0.040
**5**	-13.10<br>0.153	-12.00<br>0.283	-2.20<br>0.811	-17.50<br>**0.045**		18.08<br>0.039	17.50<br>**0.045**	12.25<br>0.210	-11.71<br>0.165	-15.00<br>0.125
**6**	4.98<br>0.587	6.08<br>0.570	15.88<br>0.083	0.58<br>0.947	18.08<br>0.039		-0.58<br>0.947	-5.83<br>0.551	-29.79<br>0.018	-33.08<br>0.032
**7**	4.40<br>0.632	5.50<br>0.608	15.30<br>0.095	0.0<br>1.000	17.50<br>**0.045**	-0.58<br>0.947		-5.25<br>0.591	-29.21<br>0.024	-32.50<br>0.040
**8**	-0.85<br>0.933	0.25<br>0.963	10.05<br>0.323	-5.25<br>0.591	12.25<br>0.210	-5.83<br>0.551	-5.25<br>0.591		-23.96<br>**0.012**	-27.25<br>**0.011**
**9**	-24.81<br>**0.005**	-23.71<br>**0.023**	-13.91<br>0.117	-29.14<br>0.024	-11.71<br>0.165	-29.79<br>0.018	-29.21<br>0.024	-23.96<br>**0.012**		-3.28<br>0.729
**10**	-28.10<br>**0.006**	-27.00<br>**0.020**	-17.20<br>0.091	-32.50<br>0.040	-15.00<br>0.125	-33.08<br>0.032	-32.50<br>0.040	-27.25<br>**0.011**	-3.28<br>0.729	

PEDAL

Cluster	1	2	3	4	5	6	7	8	9	10
**1**		-9.26<br>0.402	-17.20<br>0.073	0.73<br>0.936	-8.93<br>0.330	10.73<br>0.242	7.90<br>0.389	-1.10<br>0.914	-21.60<br>0.015	-27.60<br>0.007
**2**	-9.26<br>0.402		-7.93<br>0.473	10.00<br>0.351	0.33<br>0.975	20.00<br>0.062	17.16<br>0.109	8.16<br>0.480	-12.33<br>0.238	-18.33<br>0.133
**3**	-17.20<br>0.073	-7.93<br>0.473		17.93<br>0.051	6.26<br>0.368	27.93<br>**0.002**	25.10<br>**0.006**	16.10<br>0.113	-4.40<br>0.620	-10.40<br>0.306
**4**	0.73<br>0.936	10.00<br>0.351	17.93<br>0.051		-9.67<br>0.269	10.00<br>0.253	7.16<br>0.413	-1.83<br>0.851	-22.33<br>**0.008**	-28.33<br>**0.004**
**5**	-8.93<br>0.330	0.33<br>0.975	6.26<br>0.368	-9.67<br>0.269		19.67<br>**0.025**	16.83<br>0.054	7.83<br>0.423	-12.66<br>0.133	-18.66<br>0.056
**6**	10.73<br>0.242	20.00<br>0.062	27.93<br>**0.002**	10.00<br>0.253	19.67<br>**0.025**		-2.83<br>0.746	-11.83<br>0.226	-32.33<br>0.008	-38.33<br>0.004
**7**	7.90<br>0.389	17.16<br>0.109	25.10<br>**0.006**	7.16<br>0.413	16.83<br>0.054	-2.83<br>0.746		-9.00<br>0.358	-29.50<br>0.021	-35.50<br>0.013
**8**	-1.10<br>0.914	8.16<br>0.480	16.10<br>0.113	-1.83<br>0.851	7.83<br>0.423	-11.83<br>0.226	-9.00<br>0.358		-20.50<br>**0.031**	-25.50<br>**0.013**
**9**	-21.60<br>0.015	12.33<br>0.238	-4.40<br>0.620	-22.33<br>**0.008**	-12.66<br>0.133	-32.33<br>0.008	-29.50<br>0.021	-20.50<br>**0.031**		-6.00<br>0.528
**10**	-27.60<br>0.007	-18.33<br>0.133	-10.40<br>0.306	-28.33<br>**0.004**	-18.66<br>0.056	-38.33<br>0.004	-35.50<br>0.013	-25.50<br>**0.013**	6.00<br>0.528	

Supplementary Table 5.2 *continued*

PALL

Cluster	1	2	3	4	5	6	7	8	9	10
**1**		-12.33<br>0.265	-17.60<br>0.066	4.50<br>0.624	-8.167<br>0.373	7.00<br>0.446	0.0<br>1.000	1.50<br>0.883	-17.28<br>0.061	-29.50<br>0.004
**2**	-12.33<br>0.265		-5.27<br>0.634	16.83<br>0.116	4.16<br>0.697	19.33<br>0.071	12.33<br>0.250	13.83<br>0.232	-4.95<br>0.636	-17.16<br>0.138
**3**	-17.60<br>0.066	-5.27<br>0.634		22.10<br>0.016	9.44<br>0.304	24.60<br>0.007	17.60<br>0.055	19.10<br>0.060	0.31<br>0.972	-11.90<br>0.242
**4**	4.50<br>0.624	16.83<br>0.116	22.10<br>0.016		-12.67<br>0.148	2.50<br>0.775	-4.50<br>0.607	-3.00<br>0.759	-21.78<br>0.010	-34.00<br>0.023
**5**	-8.167<br>0.373	4.16<br>0.697	9.44<br>0.304	-12.67<br>0.148		15.16<br>0.083	8.16<br>0.351	9.67<br>0.323	-9.12<br>0.279	-21.33<br>0.029
**6**	7.00<br>0.446	19.33<br>0.071	24.60<br>0.007	2.50<br>0.775	15.16<br>0.083		-7.00<br>0.424	-5.50<br>0.574	-24.28<br>0.004	-36.50<br>0.009
**7**	0.0<br>1.000	12.33<br>0.250	17.60<br>0.055	-4.50<br>0.607	8.16<br>0.351	-7.00<br>0.424		1.50<br>0.878	-17.28<br>0.040	-29.50<br>0.003
**8**	1.50<br>0.883	13.83<br>0.232	19.10<br>0.060	-3.00<br>0.759	9.67<br>0.323	9.66<br>0.323	1.50<br>0.878		-18.78<br>0.048	-31.00<br>0.004
**9**	-17.28<br>0.061	-4.95<br>0.636	0.31<br>0.972	-21.78<br>0.010	-9.12<br>0.279	-24.28<br>0.004	-17.28<br>0.040	-18.78<br>0.048		-12.21<br>0.198
**10**	-29.50<br>0.004	-17.16<br>0.138	-11.90<br>0.242	-34.00<br>0.023	-21.33<br>0.029	-36.50<br>0.009	-29.50<br>0.003	-31.00<br>0.004	-12.21<br>0.198	

CHRF

Cluster	1	2	3	4	5	6	7	8	9	10
**1**		-19.67<br>0.085	-16.00<br>0.095	-11.90<br>0.195	-13.90<br>0.130	8.60<br>0.349	14.02<br>0.127	16.47<br>0.105	1.46<br>0.870	-4.65<br>0.647
**2**	-19.67<br>0.085		3.06<br>0.782	7.17<br>0.504	5.16<br>0.630	27.67<br>0.010	33.08<br>0.002	35.54<br>0.002	20.52<br>0.050	14.42<br>0.213
**3**	-16.00<br>0.095	3.06<br>0.782		4.10<br>0.655	2.10<br>0.819	24.60<br>0.007	30.17<br>0.046	32.47<br>0.001	17.46<br>0.049	11.35<br>0.264
**4**	-11.90<br>0.195	7.17<br>0.504	4.10<br>0.655		-2.00<br>0.819	20.50<br>0.019	25.92<br>0.003	28.37<br>0.004	13.36<br>0.113	7.25<br>0.459
**5**	-13.90<br>0.130	5.16<br>0.630	2.10<br>0.819	-2.00<br>0.819		22.50<br>0.010	27.92<br>0.001	30.37<br>0.002	15.36<br>0.069	9.25<br>0.344
**6**	8.60<br>0.349	27.67<br>0.010	24.60<br>0.007	20.50<br>0.019	22.50<br>0.010		5.42<br>0.536	7.87<br>0.421	-7.14<br>0.397	-13.25<br>0.176
**7**	14.02<br>0.127	33.08<br>0.002	30.17<br>0.046	25.92<br>0.003	27.92<br>0.001	5.42<br>0.536		2.46<br>0.802	-12.56<br>0.136	-18.67<br>0.056
**8**	16.47<br>0.105	35.54<br>0.002	32.47<br>0.001	28.37<br>0.004	30.37<br>0.002	7.87<br>0.421	2.46<br>0.802		-15.02<br>0.114	-21.12<br>0.049
**9**	1.46<br>0.870	20.52<br>0.050	17.46<br>0.049	13.36<br>0.113	15.36<br>0.069	-7.14<br>0.397	-12.56<br>0.136	-15.02<br>0.114		-6.11<br>0.520
**10**	-4.65<br>0.647	14.42<br>0.213	11.35<br>0.264	7.25<br>0.459	9.25<br>0.344	-13.25<br>0.176	-18.67<br>0.056	-21.12<br>0.049	-6.11<br>0.520	

Supplementary Table 5.2 *continued*

Optic Lobes

OPTIC

Cluster	1	2	3	4	5	6	7	8	9	10
**1**		12.87<br>0.245	14.20<br>0.138	-0.88<br>0.923	7.45<br>0.417	-13.47<br>0.142	-8.30<br>0.366	-5.55<br>0.585	19.49<br>**0.028**	27.95<br>**0.006**
**2**	12.87<br>0.245		1.33<br>0.904	-13.75<br>0.199	-5.42<br>0.613	-26.33<br>**0.014**	-21.16<br>**0.048**	-18.42<br>0.112	6.62<br>0.527	15.08<br>0.193
**3**	14.20<br>0.138	1.33<br>0.904		-15.08<br>0.100	-6.75<br>0.462	-27.67<br>**0.003**	-22.50<br>**0.014**	-19.75<br>0.052	5.28<br>0.551	13.75<br>0.176
**4**	-0.88<br>0.923	-13.75<br>0.199	-15.08<br>0.100		8.33<br>0.341	-12.58<br>0.150	-7.42<br>0.397	-4.67<br>0.633	20.38<br>**0.016**	28.83<br>**0.003**
**5**	7.45<br>0.417	-5.42<br>0.613	-6.75<br>0.462	8.33<br>0.341		-20.92<br>0.017	-15.75<br>0.072	-13.00<br>0.154	12.04<br>0.153	20.50<br>**0.036**
**6**	-13.47<br>0.142	-26.33<br>**0.014**	-27.67<br>**0.003**	-12.58<br>0.150	-20.92<br>0.017		5.17<br>0.555	7.91<br>0.418	32.95<br>**0.004**	41.42<br>**0.001**
**7**	-8.30<br>0.366	-21.16<br>**0.048**	-22.50<br>**0.014**	-7.42<br>0.397	-15.75<br>0.072	5.17<br>0.555		2.75<br>0.779	27.79<br>**0.044**	36.25<br>**0.009**
**8**	-5.55<br>0.585	-18.42<br>0.112	-19.75<br>0.052	-4.67<br>0.633	13.00<br>0.154	7.91<br>0.418	2.75<br>0.779		25.04<br>**0.006**	33.50<br>**0.002**
**9**	19.49<br>**0.028**	6.62<br>0.527	5.28<br>0.551	20.38<br>**0.016**	12.04<br>0.153	32.95<br>**0.004**	27.79<br>**0.044**	25.04<br>**0.006**		8.64<br>0.373
**10**	27.95<br>**0.006**	15.08<br>0.193	13.75<br>0.176	28.83<br>**0.003**	20.50<br>**0.036**	41.42<br>**0.001**	36.25<br>**0.009**	33.50<br>**0.002**	8.64<br>0.373	

Data resulted not-normally distributed, after Shapiro-Wilk test (Zar, 1999), in a number of cases: INFF, clusters 3 and 6; VERT, clusters 3 and 5; BRAC, cluster 5; PEDAL, cluster 3; PALL, cluster 9 (clusters 2 and 5 only marginally significant); CHRF, clusters 3 and 4 (cluster 9 only marginally significant).
In all other cases Shapiro-Wilk test confirmed the normal distribution of data.

Supplementary Table 6 – Correspondence between species included by Lindgren et al. (2012) and this study

Species are listed in alphabetical order. We search for correspondence of taxa between the tree of Lindgren et al. (2012) and the 78 species considered in this study (see Table 1). Matching of species was found only in 38 cases. Correspondence at genus level is indicated, whenever the case; other species because considered sister to a given taxon are also listed. We did not found representatives in the relative Families for: *Bathothauma lyromma*, sister to *Helicocranchia*; *Helicocranchia papillate,* sister to *Bathotauma*; *Pickfordiateuthis pulchella*; *Pteroctopus tetracirrhus*; *Sandalops melancholicus, s*ister to *Bathotauma* + *Megalocranchia*; *Scaeurgus unicirrhus*.

Lindgren et al. (2012)	Species matching (this study)	Genus matching (this study)	Other taxa	Notes
Abralia_veranyi	*Abralia (Asteroteuthis) veranyi*			
Abraliopsis_pacificus		*Abraliopsis (Micrabralia) ?morisi*		
Adelieledone_piatkowski				
Adelieledone_polymorpha				
Amphioctopus_aegina				
Ancistroteuthis_lichtensteinii	*Ancistroteuthis lichtensteini*			
Architeuthis_dux	*Architeuthis dux*			
Architeuthis_sp_ARL_2008				
Architeuthis_sp_JMS_2004				
Argonauta_nodosa		*Argonauta argo*	*Ocythoe tuberculata*	Sister to *Argonauta*
Asperoteuthis_nesisi				
Bathypolypus_arcticus				
Bathypolypus_sp_JMS_2004		*Bathypolypus sponsalis*		
Bathypolypus_valdiviae				
Bathyteuthis_abyssicola				

Lindgren et al. (2012)	Species matching (this study)	Genus matching (this study)	Other taxa	Notes
Bathyteuthis_berryi				
Bathyteuthis_sp_A		*Bathyteuthis sp.*		
Batoteuthis_skolops				
Benthoctopus_eureka				
Benthoctopus_johnsonianus				
Benthoctopus_normani				
Benthoctopus_sp_A_JMS_2004		*Benthoctopus piscatorum*		
Benthoctopus_sp_JMS_2004				
Benthoctopus_yaquinae				
Berryteuthis_anonychus				
Berryteuthis_magister				
Bolitaena_pygmaea		*Eledonella sp.*		
Brachioteuthis_sp_2		*Brachioteuthis riisei*		
Brachioteuthis_sp_3				
Callistoctopus_ornatus				
Chiroteuthis_calyx				
Chiroteuthis_mega				
Chiroteuthis_veranyi	*Chiroteuthis (Chiroteuthis) veranyi veranyi*			
Chtenopteryx_sicula	*Chtenopteryx sicula*			
Chtenopteryx_sp_ARL_2008				
Cirrothauma_murrayi	*Cirrothauma murrayi*		*Cirroteuthis sp.*	Sister to *Cirrothauma*
Cistopus_sp_JMS_2004				
Cranchia_scabra	*Cranchia scabra*			
Cycloteuthis_sirventyi				
Discoteuthis_discus				
Discoteuthis_laciniosa	*Discoteuthis laciniosa*			

Lindgren et al. (2012)	Species matching (this study)	Genus matching (this study)	Other taxa	Notes
Doryteuthis_opalescens				
Doryteuthis_pealeii				
Dosidicus_gigas				
Eledone_cirrhosa	*Eledone cirrhosa*	*Eledone moschata*		
Enoploteuthis_higginsi				
Enoploteuthis_leptura				
Enteroctopus_dofleini	*Enteroctopus dofleini*			
Eucleoteuthis_luminosa				
Euprymna_berryi				
Euprymna_hyllebergi				
Euprymna_scolopes				
Euprymna_tasmanica				
Galiteuthis_armata				
Galiteuthis_sp_JMS_2004		*Galiteuthis glacialis*		
Gonatopsis_octopedatus				
Gonatopsis_sp_ARL_2008				
Gonatus_antarcticus				
Gonatus_fabricii	*Gonatus (Gonatus) fabricii*			
Graneledone_antarctica				
Graneledone_boreopacifica				
Graneledone_verrucosa				
Grimalditeuthis_bonplandi	*Grimalditeuthis bonplandi*			
Haliphron_atlanticus	*Haliphron atlanticus*			
Hapalochlaena_maculosa				
Heterololigo_bleekeri				
Heteroteuthis_hawaiiensis		*Heteroteuthis (Heteroteuthis) dispar*		

Lindgren et al. (2012)	Species matching (this study)	Genus matching (this study)	Other taxa	Notes
Histioteuthis_bonellii				
Histioteuthis_corona				
Histioteuthis_hoylei				
Histioteuthis_miranda	*Histioteuthis miranda*			
Histioteuthis_oceanica				
Histioteuthis_reversa				
Idiosepius_notoides				
Idiosepius_pygmaeus				
Illex_coindetii	*Illex coindetii*	*Illex illecebrosus*		
Japetella_diaphana	*Japetella sp.*			
Joubiniteuthis_portieri	*Joubiniteuthis portieri*			
Kondakovia_sp_ARL_2008				
Leachia_atlantica		*Leachia (Pyrgopsis) pacifica*		
Leachia_lemur				
Lepidoteuthis_grimaldii				
Loligo_forbesii	*Loligo? forbesii*	*Loligo (Alloteuthis) media*		
Loligo_vulgaris	*Loligo (Loligo) vulgaris vulgaris*	*Loligo (Alloteuthis) subulata*		
Loliolus_sp_JMS_2004				
Lolliguncula_brevis	*Lolliguncula (Lolliguncula) brevis*			
Lolliguncula_diomedeae				
Lycoteuthis_lorigera	*Lycoteuthis lorigera*			
Magnapinna_sp_ARL_2008				
Mastigoteuthis_agassizii				
Mastigoteuthis_hjorti				
Mastigoteuthis_magna		*Mastigoteuthis ?schmidti*		
Megaleledone_setebos				

Lindgren et al. (2012)	Species matching (this study)	Genus matching (this study)	Other taxa	Notes
Megalocranchia_fisheri				
Megalocranchia_sp_ARL_2008		*Megalocranchia sp.*	*Egea inermis*	Sister to *Megalocranchia*
Mesonychoteuthis_hamiltoni				
Metasepia_tullbergi				
Moroteuthis_knipovitchi				
Nautilus_macromphalus				
Nautilus_pompilius				
Neoteuthis_thielei	*Neoteuthis thielei*			
Notonykia_sp_ARL_2008				
Octopoteuthis_megaptera		*Octopoteuthis danae*		
Octopoteuthis_nielseni				
Octopoteuthis_sicula	*Octopoteuthis sicula*			
Octopus_berrima				
Octopus_bimaculoides		*Octopus (Octopus) bimaculatus*		
Octopus_cyanea		*Octopus (Octopus) defilippi*		
Octopus_kaurna		*Octopus (Octopus) macropus*		
Octopus_ocellatus		*Octopus (Octopus) salutii*		
Octopus_rubescens				
Octopus_vulgaris	*Octopus (Octopus) vulgaris*			
Ommastrephes_bartramii				
Onychoteuthis_banksii	*Onychoteuthis banksii*			
Onychoteuthis_sp_B3_JMS_2004				
Onykia_carriboea				
Onykia_robusta				
Opisthoteuthis_massyae		*Opisthoteuthis sp.*	*Grimpoteuthis sp.*	Sister to *Opisthoteuthis*
Ornithoteuthis_antillarum				

Lindgren et al. (2012)	Species matching (this study)	Genus matching (this study)	Other taxa	Notes
Pareledone_aequipapillae				
Pareledone_albimaculata				
Pareledone_aurata				
Pareledone_charcoti				
Pareledone_cornuta				
Pareledone_felix				
Pareledone_panchroma				
Pareledone_serperastrata				
Pareledone_subtilis				
Pareledone_turqueti				
Pholidoteuthis_adami				
Planctoteuthis_levimana				
Psychroteuthis_glacialis				
Pterygioteuthis_gemmata				
Pterygioteuthis_giardi_hoylei	*Pterygioteuthis giardi giardi*		*Pyroteuthis margaritifera*	Sister to *Pterygioteuthis*
Pterygioteuthis_microlampas				
Rondeletiola_minor				
Rossia_macrosoma	*Rossia macrosoma*		*Neorossia caroli*	Sister to *Rossia*
Rossia_pacifica				
Rossia_palpebrosa				
Selenoteuthis_scintillans				
Sepia_apama				
Sepia_elegans	*Sepia (Rhombosepion) elegans*			
Sepia_esculenta				
Sepia_kobiensis				
Sepia_latimanus				

Lindgren et al. (2012)	Species matching (this study)	Genus matching (this study)	Other taxa	Notes
Sepia_lorigera				
Sepia_lycidas		*Sepia (Rhombosepion) orbignyana*		
Sepia_officinalis	*Sepia (Sepia) officinalis*			
Sepia_pardex				
Sepia_peterseni				
Sepia_pharaonis				
Sepia_recurvirostra				
Sepia_sp_SI0604				
Sepiadarium_austrinum				
Sepiadarium_kochi				
Sepiella_inermis				
Sepiella_japonica				
Sepietta_neglecta		*Sepietta oweniana*		
Sepietta_obscura				
Sepiola_affinis	*Sepiola affinis*			
Sepiola_atlantica				
Sepiola_birostrata				
Sepiola_intermedia				
Sepiola_ligulata				
Sepiola_robusta	*Sepiola robusta*			
Sepiola_sp_JMS_2004		*Sepiola rondeleti*		
Sepiolina_nipponensis				
Sepioloidea_lineolata				
Sepioteuthis_australis				
Sepioteuthis_lessoniana		*Sepioteuthis sepioidea*		
Spirula_spirula	*Spirula spirula*			

Lindgren et al. (2012)	Species matching (this study)	Genus matching (this study)	Other taxa	Notes
Stauroteuthis_gilchristi				
Stauroteuthis_syrtensis				
Sthenoteuthis_oualaniensis				
Stoloteuthis_leucoptera				
Taningia_danae				
Taonius_pavo	*Taonius pavo*			
Teuthowenia_megalops	*Teuthowenia megalops*			
Thaumeledone_gunteri				
Thaumeledone_peninsulae				
Thaumeledone_rotunda				
Thysanoteuthis_rhombus				
Todarodes_pacificus		*Todarodes sagittatus*		
Todaropsis_eblanae	*Todaropsis eblanae*			
Tremoctopus_violaceus	*Tremoctopus violaceus violaceus*			
Uroteuthis_chinensis				
Uroteuthis_noctiluca				
Uroteuthis_sp_JMS_2004				
Vampyroteuthis_infernalis	*Vampyroteuthis infernalis*			
Velodona_togata				
Vitreledonella_richardi	*Vitreledonella richardi*		*Amphitretus sp.*	
Watasenia_scintillans				

Considerations taken for the phylogenetic PCA and subsequent analysis

To attempt to control for phylogenetic dependence/independence of traits here considered (brains' diversity) and possibly ruling out bias in detecting relationships and inaccurate estimates of correlations (Rezende and Diniz-Filho, 2012), we ran phylogenetic principal component analysis (pPCA, Revell, 2009) to account for the phylogenetic relationship between species. We utilized as reference tree the multigene phylogeny based on maximum likelihood analysis published by Lindgren et al. (2012). The original tree comprises 188 taxa (see Fig. 1 in Lindgren et al., 2012), however only 38 over 78 species matched in our dataset (see Supplementary Table 6).

To derive the phylogeny to input in the "phyloPCA" (pPCA, Revell, 2009) we pruned the tree including exclusively the 38 matching species (Supplementary Figure 1).

For pPCA we utilized the phytools package (https://CRAN.R-project.org/package=phytools; September, 2020) using the following setting: method="lambda", mode="corr", rotate="varimax".

Data for the brain functional sets were included, for the corresponding species.

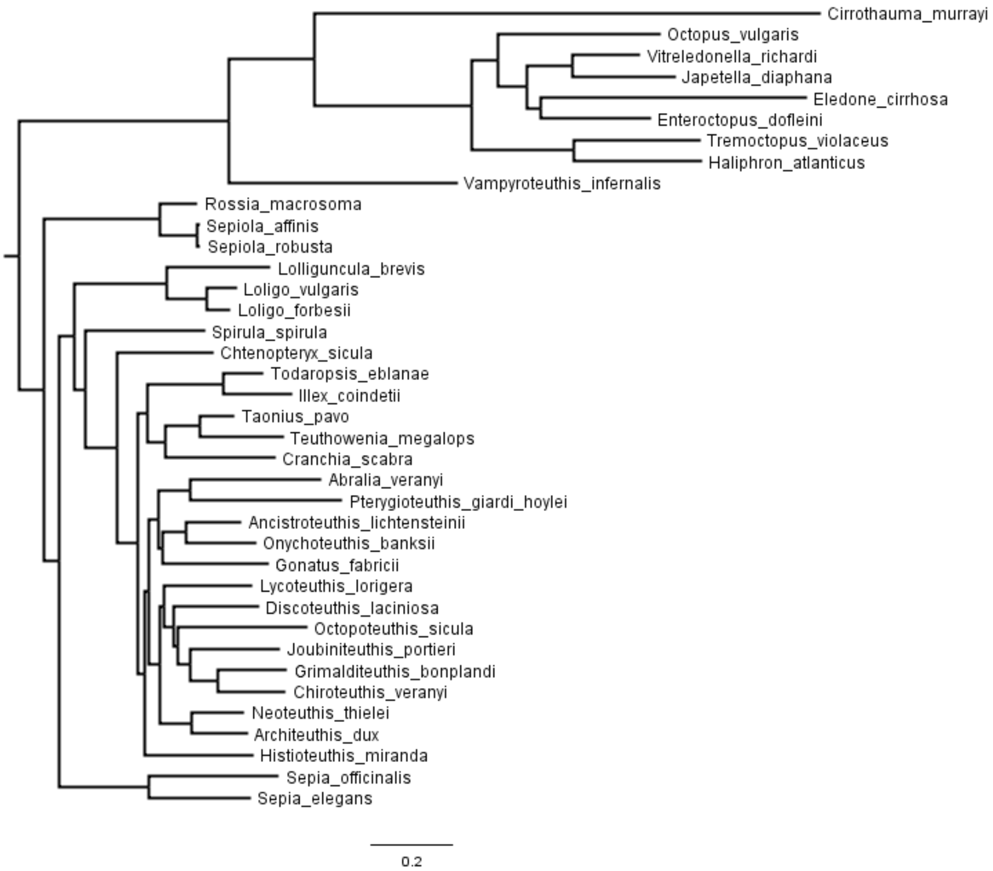

Supplementary Figure 1. Pruned tree after Lindgren et al. (2012) after considering the 38 species found as corresponding to the list of organisms included in this study; see also Table 1 and Supplementary Table 6.

Scores resulting after pPCA for the first three components were extracted as shown in Supplementary Table 7 below (species listed in alphabetical order).

Supplementary Table 7. Phylogenetic PCA scores of species, only the values for the first three components are included.

	pPC1	pPC2	pPC3
Abralia veranyi	-0.06915	-0.73652	-0.31956
Ancistroteuthis lichtensteinii	-0.15503	-0.48481	-0.95828
Architeuthis dux	-1.27657	0.257592	1.634141
Chiroteuthis veranyi	1.969548	0.527644	-0.1202
Chtenopteryx sicula	1.963534	0.238822	-0.23484
Cirrothauma murrayi	-7.8373	4.591601	-1.68105
Cranchia scabra	2.877321	0.665529	-0.25219
Discoteuthis laciniosa	1.492348	0.280381	-0.86167
Eledone cirrhosa	-2.64373	2.022464	0.677339

	pPC1	pPC2	pPC3
Enteroctopus dofleini	-4.52213	3.346694	1.009412
Gonatus fabricii	2.036626	0.746599	-0.18195
Grimalditeuthis bonplandi	3.009387	0.82136	-0.41808
Haliphron atlanticus	2.017084	0.839285	-0.27681
Histioteuthis miranda	1.457313	0.313625	0.040498
Illex coindetii	-0.7607	-0.40843	-0.80431
Japetella diaphana	0.186273	1.660904	0.306251
Joubiniteuthis portieri	-0.77659	0.630642	-0.45304
Loligo forbesii	1.651305	0.037413	0.353011
Loligo vulgaris	-0.94065	-0.75734	-0.37677
Lolliguncula brevis	-0.29568	-0.12151	0.606935
Lycoteuthis lorigera	2.071244	0.5365	0.020921
Neoteuthis thielei	1.917181	0.526561	-0.51336
Octopoteuthis sicula	-1.78484	-0.93576	-1.19859
Octopus vulgaris	-2.97122	1.822172	0.529291
Onychoteuthis banksii	0.728493	0.043432	-0.68537
Pterygioteuthis giardia	1.585731	0.271784	-0.45433
Rossia macrosoma	-1.65028	-0.42527	-0.07517
Sepia elegans	-0.50969	-1.39818	0.899703
Sepia officinalis	-0.75731	-1.23419	1.552166
Sepiola affinis	-0.58436	-0.66868	-0.94593
Sepiola robusta	-0.702	-0.30786	-0.96709
Spirula spirula	0.781595	0.096941	0.105507
Taonius pavo	2.378843	0.832133	-0.00746
Teuthowenia megalops	2.110621	0.666058	-0.57947
Todaropsis eblanae	-0.3088	-0.15012	-0.46873
Tremoctopus violaceus	1.196267	0.410326	0.084436
Vampyroteuthis infernalis	1.863827	0.352184	0.973275
Vitreledonella richardi	1.155245	0.661702	0.001166

Following phylogenetic PCA we ran again the cluster analysis, but utilizing a further reduced dataset. In fact, from the 38 species utilized in the pPCA we excluded the ones not included in the original cluster analysis (Figure 3). The final number of species used to perform the further cluster analysis resulted to be 24. The cluster (see Supplementary Figure 2) was obtained using “pvclust” (Clustering method Ward’s minimum variance method, with dissimilarities squared before clustering; Ward.D2 in

“pvclust” package; number of bootstraps=100,000).

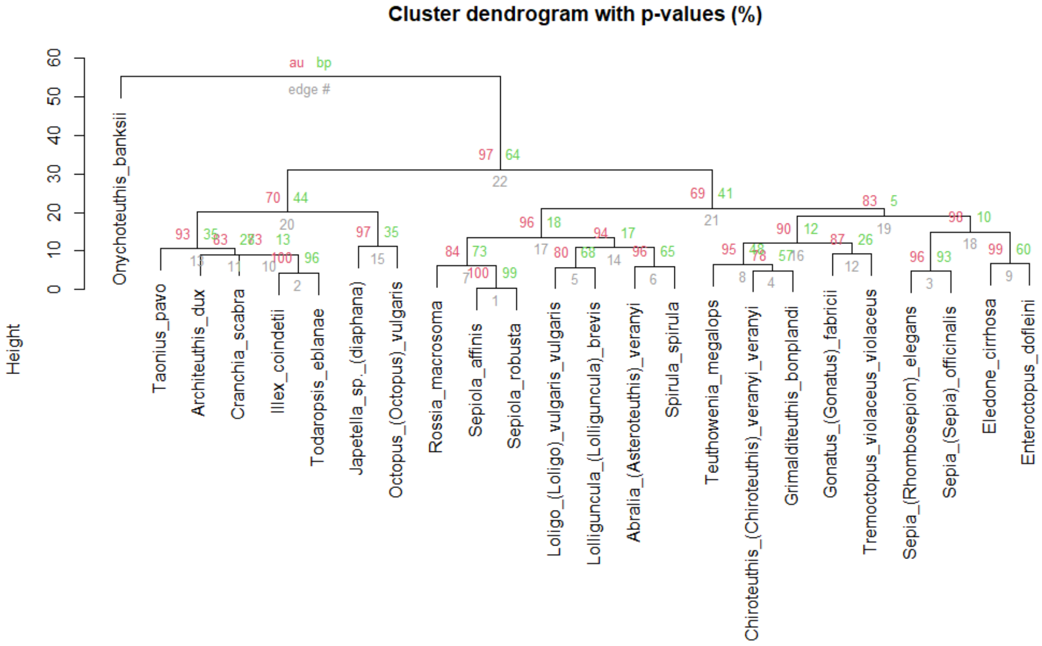

Supplementary Figure 2. Hierarchical cluster (Ward’s Method) of the 24 species found as corresponding to the list of organisms included in this study after pruning Lindgren et al. (2012) tree (see Supplementary Figure 1) and the ones included in the final dendrogram of Figure 3. The topology of the resulting dendrogram is different from the one produced by our original cluster analysis (see Figure 3), but also from the phylogenetic tree (refer to Supplementary Figure 1) and the original by Lindgren et al. (2012). See main text for details.

List of References

Abbott, N.J., and Pichon, Y. (1987). The glial blood-brain barrier of crustacea and cephalopods: a review. *J. Physiol. Paris* 82**,** 304-313.

Arkhipkin, A. (2004). Diversity in growth and longevity in short-lived animals: squid of the suborder Oegopsina. *Marine and Freshwater Research* 55**,** 341-355.

Basagaña, X., Barrera-Gómez, J., Benet, M., Antó, J.M., and Garcia-Aymerich, J. (2013). A Framework for Multiple Imputation in Cluster Analysis. *American Journal of Epidemiology* 177(7)**,** 718-725. doi: 10.1093/aje/kws289.

Boletzky, S.v. (1981). Réflexions sur les stratégies de reproduction chez les Céphalopodes. *Bull. Soc. Zool. Fr* 106**,** 293-304.

Boletzky, S.v. (1998). Cephalopod eggs and egg masses. *Oceanogr. Mar. Biol. Annu. Rev* 36**,** 341-371.

Boluki, S., Zamani Dadaneh, S., Qian, X., and Dougherty, E.R. (2019). Optimal clustering with missing values. *BMC Bioinformatics* 20(12)**,** 321. doi: 10.1186/s12859-019-2832-3.

Borrelli, L. (2007). *Testing the contribution of relative brain size and learning capabilities on the evolution of Octopus vulgaris and other cephalopods.* PhD PhD Thesis, Stazione Zoologica Anton Dohrn, Italy & Open University, UK.

Budelmann, B.U. (1995). "The cephalopod nervous system: what evolution has made of the molluscan design," in *The Nervous Systems of Invertebrates: An Evolutionary and Comparative Approach,* eds. O. Breidbach & W. Kutsch. (Basel, Switzerland: Birkhäuser Verlag), p. 115-138.

Bullock, T.H. (1965). "Mollusca: Cephalopoda," in *Structure and Function in the Nervous Systems of Invertebrates,* eds. T.H. Bullock & G.A. Horridge. (San Francisco and London: W.H. Freeman and Company), p. 1433-1515.

Clark, D.A., Mitra, P.P., and Wang, S.S.-H. (2001). Scalable architecture in mammalian brains. *Nature* 411**,** 189-193.

Clarke, M.R. (1986). *A Handbook for the identification of cephalopod beaks.* Oxford: Oxford University Press.

Clarke, M.R. (1988). "Evolution of buoyancy and locomotion in recent cephalopods," in *The Mollusca. Paleontology and Neontology of Cephalopods,* eds. M.R. Clarke & E.R. Trueman. (San Diego: Academic Press, Inc.), p. 203-213.

Clarke, M.R., Denton, E.J., and Gilpin-Brown, J.B. (1979). On the use of ammonium for buoyancy in squids. *J. Mar. Biol. Assoc. U. K* 59**,** 259-276.

Clarke, M.R., and Maddock, L. (1988). "Beaks of living coleoid Cephalopoda," in *The Mollusca. Paleontology and Neontology of Cephalopods,* eds. M.R. Clarke & E.R. Trueman. (San Diego: Academic Press, Inc.), p. 123-131.

Deryckere, A., and Seuntjens, E. (2018). The Cephalopod Large Brain Enigma: Are Conserved Mechanisms of Stem Cell Expansion the Key? *Frontiers in Physiology* 9(1160). doi: 10.3389/fphys.2018.01160.

Dickel, L., Chichery, M.P., and Chichery, R. (1997). Postembryonic maturation of the vertical lobe complex and early development of predatory behavior in the cuttlefish (*Sepia officinalis*). *Neurobiol. Learn. Mem* 67**,** 150-160.

Dickel, L., Darmaillacq, A.S., Poirier, R., Agin, V., Bellanger, C., and Chichery, R. (2006). Behavioural and neural maturation in the cuttlefish *Sepia officinalis*. *Vie et Milieu* 56**,** 89-95.

Dunn, O.J. (1964). Multiple Comparisons Using Rank Sums. *Technometrics* 6(3)**,** 241-252. doi: 10.1080/00401706.1964.10490181.

Everitt, B., Landau, S., and Leese, M. (2001). *Cluster Analysis.* London: Edward Arnold Publishers Ltd.

Everitt, B.S. (1993). *Cluster Analysis.* London: Edward Arnold Publishers Ltd.

Fiero, M.H., Huang, S., Oren, E., and Bell, M.L. (2016). Statistical analysis and handling of missing data in cluster randomized trials: a systematic review. *Trials* 17(1)**,** 72. doi: 10.1186/s13063-016-1201-z.

Forsythe, J.W. (1993). "A working hypothesis on how seasonal temperature change may impact the field growth of young cephalopods," in *Recent Advances in Cephalopod Fisheries Biology,* eds. T. Okutani, R.K. O'Dor & T. Kubodera. (Tokyo: Tokai University Press), p. 133-143.

Forsythe, J.W. (2004). Accounting for the effect of temperature on squid growth in nature: from hypothesis to practice. *Marine and Freshwater Research* 55**,** 331-339.

Forsythe, J.W., and Hanlon, R.T. (1988). Effect of temperature on laboratory growth, reproduction and life span of *Octopus bimaculoides*. *Mar. Biol* 98**,** 369-379.

Forsythe, J.W., and Van Heukelem, W.F. (1987). "Growth," in *Cephalopod Life Cycles. Comparative Reviews,* ed. P.R. Boyle. (London: Academic Press), p. 135-156.

Frösch, D. (1971). Quantitative Untersuchungen am Zentralnervensystem der Schlüpfstadien von zehn mediterranen Cephalopodenarten. *Rev. Suisse Zool* 78**,** 1069-1122.

Giuditta, A., Libonati, M., Packard, A., and Prozzo, N. (1971). Nuclear counts in the brain lobes of *Octopus vulgaris* as a function of body size. *Brain Research* 25**,** 55-62.

Gorsuch, R.L. (1983). *Factor Analysis.* Hillsdale, NJ: Lawrence Erlbaum Associates.

Gould, S.J. (1966). Allometry and size in ontogeny and phylogeny. *Biol. Rev* 41**,** 587-640.

Guerra, Á. (1979). Fitting a von Bertalanffy expression to *Octopus vulgaris* growth. *Investig. Pesq* 43**,** 319-326.

Hanlon, R.T., and Messenger, J.B. (1996). *Cephalopod Behaviour.* Cambridge: Cambridge University Press.

Hanlon, R.T., and Messenger, J.B. (2018). *Cephalopod Behaviour.* Cambridge: Cambridge University Press.

Hochberg, F.G., Nixon, M., and Toll, R.B. (1992). "Family Accounts: Order Octopoda Leach, 1818," in *"Larval" and Juvenile Cephalopods: A Manual for Their Identification,* eds. M.J. Sweeney, C.F.E. Roper, K.M. Mangold, M.R. Clarke & S.v. Boletzky. (Washington, D.C.: Smithsonian Institution), p. 213-280.

Hochner, B., Shomrat, T., and Fiorito, G. (2006). The octopus: a model for a comparative analysis of the evolution of learning and memory mechanisms. *The Biological Bulletin* 210(3)**,** 308-317.

Huber, R., van Staaden, M.J., Kaufman, L.S., and Liem, K.F. (1997). Microhabitat use, trophic patterns, and the evolution of brain structure in African cichlids. *Brain Behav. Evol.* 50**,** 167-182.

Hughes, R.A., Heron, J., Sterne, J.A.C., and Tilling, K. (2019). Accounting for missing data in statistical analyses: multiple imputation is not always the answer. *International Journal of Epidemiology* 48(4)**,** 1294-1304. doi: 10.1093/ije/dyz032.

ICES (2019). *Interim Report of the Working Group on Cephalopod Fisheries and Life History (WGCEPH).* Copenhagen, Denmark: International Council for the Exploration of the Sea.

Jaaro, H., and Fainzilber, M. (2006). Building complex brains - missing pieces in an evolutionary puzzle. *Brain Behav. Evol.* 68**,** 191-195.

Jereb, P., and Roper, C. (2005). *Cephalopods of the world. An annotated and illustrated catalogue of species known to date. Volume 1. Chambered Nautiluses and Sepioids (Nautilidae, Sepiidae, Sepiolidae, Sepiadariidae, Idiosepiidae and Spirulidae).* Rome, Italy: FAO, Food and Agriculture Organization of the United Nations.

Jereb, P., and Roper, C. (2010). *Cephalopods of the world. An annotated and illustrated catalogue of species known to date. Volume 2. Myopsid and Oegopsid Squids.* Rome, Italy: FAO.

Jereb, P., Roper, C., Norman, M., and Finn, J. (2016). *Cephalopods of the World. An Annotated and Illustrated Catalogue of Species Known to Date. Volume 3. Octopods and Vampire Squids.* Roma, Italy: FAO, Food and Agriculture Organization of the United Nations.

Kandel, E.R. (1979). *Behavioral Biology of Aplysia. A contribution to the comparative study of opisthobranch molluscs.* San Francisco: W.H. Freeman and Company.

Katz, P.S. (2007). Evolution and development of neural circuits in invertebrates. *Current Opinion in Neurobiology* 17(1)**,** 59-64. doi: https://doi.org/10.1016/j.conb.2006.12.003.

Kear, A.J. (1992). The diet of Antarctic squid: comparison of conventional and serological gut contents analyses. *J. Exp. Mar. Biol. Ecol* 156**,** 161-178.

Killam, D.E., and Clapham, M.E. (2018). Identifying the ticks of bivalve shell clocks: seasonal growth in relation to temperature and food supply. *Palaios* 33(5)**,** 228-236.

Laptikhovsky, V.V. (2002). Ecology of decapod cephalopod reproduction (Cephalopoda: Teuthida, Sepiida). *Zoologicheskii Zhurnal* 81**,** 1319-1328.

Lazareth, C.E., Lasne, G., and Ortlieb, L. (2006). Growth anomalies in *Protothaca thaca* (Mollusca, Veneridae) shells as markers of ENSO conditions. *Climate Research* 30**,** 263-269.

Lindgren, A.R., Pankey, M.S., Hochberg, F.G., and Oakley, T.H. (2012). A multi-gene phylogeny of Cephalopoda supports convergent morphological evolution in association with multiple habitat shifts in the marine environment. *BMC Evolutionary Biology* 12(1)**,** 129.

Lipinski, M.R., and Roeleveld, M.A. (1990). Minor extension of the von Bertalanffy growth theory. *Fisheries Research (Amsterdam)* 9**,** 367-371.

Lordan, C., Burnell, G.M., and Cross, T.F. (1998). The diet and ecological importance of *Illex coindetii* and *Todaropsis eblanae* (Cephalopoda: Ommastrephidae) in Irish waters. *S. Afr. J. Mar. Sci* 20**,** 153-163.

Lu, C.C., Guerra, Á., Palumbo, F., and Summers, W.C. (1992). "Family Accounts: Order Sepioidea Naef, 1916," in *"Larval" and Juvenile Cephalopods: A Manual for their Identification,* eds. M.J. Sweeney, C.F.E. Roper, K.M. Mangold, M.R. Clarke & S.v. Boletzky. (Washington, D.C.: Smithsonian Institution), p. 21-36.

Lu, C.C., and Williams, R. (1994). Contribution to the biology of squid in the Prydz Bay region, Antarctica. *Antarctic Science* 6**,** 223-229.

MacArthur, R.H., and Wilson, E.O. (1967). *The theory of island biogeography.* Princeton, N.J.: Princeton University Press.

Maddock, L., and Young, J.Z. (1984). Some dimensions of the angular acceleration receptor systems of cephalopods. *J. Mar. Biol. Assoc. U. K* 64**,** 55-79.

Maddock, L., and Young, J.Z. (1987). Quantitative differences among the brains of cephalopods. *J. Zool* 212**,** 739-767.

Mangold, K. (1987). "Reproduction," in *Cephalopod Life Cycles. Comparative Reviews,* ed. P.R. Boyle. (London: Academic Press), p. 157-200.

Manly, C.A., and Wells, R.S. (2015). Reporting the Use of Multiple Imputation for Missing Data in Higher Education Research. *Research in Higher Education* 56(4)**,** 397-409. doi: 10.1007/s11162-014-9344-9.

Mares, S., Ash, L., and Gronenberg, W. (2005). Brain allometry in bumblebee and honey bee workers. *Brain Behav. Evol.* 66**,** 50-61.

Marino, L. (1998). A comparison of encephalization between odontocete cetaceans and anthropoid primates. *Brain Behav. Evol.* 51**,** 230-238.

Messenger, J. (1979). The nervous system of *Loligo*: IV. The peduncle and olfactory lobes. *Philosophical Transactions of the Royal Society of London B: Biological Sciences* 285(1008)**,** 275-309.

Messenger, J.B. (1973). Learning in the cuttlefish, *Sepia*. *Animal Behaviour* 21(4), 801-826. doi: https://doi.org/10.1016/S0003-3472(73)80107-1.

Messenger, J.B. (1996). Neurotransmitters of cephalopods. *Invertebr. Neurosci* 2, 95-114.

Messenger, J.B., and Young, J.Z. (1999). The radular apparatus of cephalopods. *Philos. Trans. R. Soc. Lond. B* 354, 161-182.

Moltschaniwskyj, N.A. (2004). Understanding the process of growth in cephalopods. *Marine and Freshwater Research* 55, 379-386.

Nesis, K.N. (1987). *Cephalopods of the world: squids, cuttelfishes, octopuses, and allies.* Neptune City, N.J.: T.F.H. Publications.

Nesis, K.N. (1995). Mating, spawning, and death in oceanic cephalopods: a review. *Ruthenica* 6, 23-64.

Nesis, K.N. (2002). Life style strategies of recent cephalopods: a review. *Bull. Mar. Sci* 71, 561-579.

Nesis, K.N. (2003). Distribution of recent cephalopoda and implications for Plio-Pleistocene events. *Berliner Palaeobiologische Abhandlungen* 3, 199-224.

Nixon, M. (1987). "Cephalopod diets," in *Cephalopod Life Cycles. Comparative Reviews.,* ed. P.R. Boyle. (London: Academic Press, Inc.), p. 201-219.

Nixon, M. (1998). "The radulae of Cephalopoda," in *Systematics and Biogeography of Cephalopods,* eds. N.A. Voss, M. Vecchione, R.B. Toll & M.J. Sweeney. (Washington, D.C.: Smithsonian Institution), p. 39-53.

Nixon, M., and Dilly, P.N. (1977). Sucker surfaces and prey capture. *Symp. Zool. Soc. Lond* 38, 447-511.

Nixon, M., and Young, J.Z. (2003). *The brains and lives of Cephalopods.* New York: Oxford University.

O'Shea, S., Lu, C.C., and Clarke, M.C. (2006). "Description of paralarval *Architeuthis* and Onychoteuthidae squid (Cephalopoda: Teuthoidea) from New Zealand waters", in: *CIAC 2006 Meeting*, (Hobart, Tasmania: CIAC 2006 Meeting), p.

Olkowicz, S., Kocourek, M., Lučan, R.K., Porteš, M., Fitch, W.T., Herculano-Houzel, S., and Němec, P. (2016). Birds have primate-like numbers of neurons in the forebrain. *Proceedings of the National Academy of Sciences* 113(26), 7255-7260. doi: 10.1073/pnas.1517131113.

Owen, R. (1832). *Memoir on the Pearly Nautilus (Nautilus Pompilius, Linn.).* London: Richard Taylor.

Packard, A., and Albergoni, V. (1970). Relative growth, nucleic acid content and cell numbers of the brain in *Octopus vulgaris* (Lamarck). *Journal of Experimental Biology* 52(3), 539-552.

Pauly, D., Zeller, D., and Palomares, M.L.D. 2020. Sea Around Us Concepts, Design and Data. *Sea Around Us Project. A global database on marine fisheries, ecosystems and biodiversity* [Online]. [Accessed May, 2020].

Pecl, G.T., Steer, M.A., and Hodgson, K.E. (2004). The role of hatchling size in generating the intrinsic size-at-age variability of cephalopods: extending the Forsythe Hypothesis. *Marine and Freshwater Research* 55**,** 387-394.

Pelseneer, P. (1888). Sur la valeur morphologique des bras et la composition du système nerveux central des Céphalopodes. *Archives de Biologie* 8**,** 723-756.

Pianka, E.R. (1970). On r- and K-selection. *Am. Nat* 104**,** 592-597.

Plän, T. (1987). *Funktionelle Neuroanatomie sensorisch/motorischer loben im gehirn von Octopus vulgaris.* Doktorgrades der Naturwissehschaften (Dr. Rer. Nat.), Universität Regensburg.

Ponte, G. (2012). *Distribution and preliminary functional analysis of some modulators in the cephalopod mollusc Octopus vulgaris.* PhD PhD Thesis, Università della Calabria, Italy; Stazione Zoologica Anton Dohrn, Napoli, Italy;.

Ponte, G., and Fiorito, G. (2015). "Immunohistochemical Analysis of Neuronal Networks in the Nervous System of *Octopus vulgaris*," eds. A. Merighi & L. Lossi.), p. 61-77.

Portmann, A. (1947). Études sur la cérébralisation chez les oiseaux. II. Les indices intracérébraux. *Alauda* 15**,** 1-15.

Quetglas, A., González, M., and Franco, I. (2005). Biology of the upper-slope cephalopod *Octopus salutii* from the western Mediterranean Sea. *Mar. Biol* 146**,** 1131-1138.

Revell, L.J. (2009). Size-Correction and Principal Components for Interspecific Comparative Studies. *Evolution* 63(12)**,** 3258-3268. doi: https://doi.org/10.1111/j.1558-5646.2009.00804.x.

Rezende, E.L., and Diniz-Filho, J.A.F. (2012). Phylogenetic Analyses: Comparing Species to Infer Adaptations and Physiological Mechanisms. *Comprehensive Physiology* 2**,** 639-674. doi: https://doi.org/10.1002/cphy.c100079.

Richardson, C.A. (2001). Molluscs as archives of environmental change. *Oceanogr. Mar. Biol. Annu. Rev* 39**,** 103-164.

Ringvold, H., and Taite, M. (2018). Using citizen science to obtain data on large, floating gelatinous spheres from NE Atlantic, attributed to egg mass of ommastrephid squid (Oegopsida, Cephalopoda, Mollusca). *Marine Biology Research* 14(7)**,** 672-681. doi: 10.1080/17451000.2018.1504165.

Rocha, F., Guerra, A., and Gonzalez, A.F. (2001). A review of reproductive strategies in cephalopods. *Biological Reviews* 76(3)**,** 291-304.

Roper, C.F.E., and Sweeney, M.J. (1992). "Family Accounts: Family Brachioteuthidae Pfeffer, 1908," in *"Larval" and Juvenile Cephalopods: A Manual for their Identification,* eds. M.J. Sweeney, C.F.E. Roper, K.M. Mangold, M.R. Clarke & S.v. Boletzky. (Washington, D.C.: Smithsonian Institution), p. 157-159.

Roper, C.F.E., Sweeney, M.J., and Nauen, C.E. (1984). *FAO Species Catalogue, vol. 3. Cephalopods of the world. An annotated and illustrated catalogue of species of interest to fisheries.*: FAO Fisheries Synopsis.

Sea Around Us. 2016. Web Products: Large Marine Ecosystems. *Sea Around Us Project. A global database on marine fisheries and ecosystems* [Online]. Available: GFLib: FolderLuPhD-S.

Seibel, B.A., Goffredi, S.K., Thuesen, E.V., Childress, J.J., and Robinson, B.H. (2004). Ammonium content and buoyancy in midwater cephalopods. *J. Exp. Mar. Biol. Ecol* 313**,** 375-387.

Semmens, J.M., Pecl, G.T., Villanueva, R., Jouffre, D., Sobrino, I., Wood, J.B., and Rigby, P.R. (2004). Understanding octopus growth: patterns, variability and physiology. *Marine and Freshwater Research* 55**,** 367-377.

Sherman, K., and Duda, A.M. (1999). An ecosystem approach to global assessment and management of coastal waters. *Mar. Ecol. Prog. Ser* 190**,** 271-287.

Shigeno, S., Andrews, P.L.R., Ponte, G., and Fiorito, G. (2018). Cephalopod Brains: An Overview of Current Knowledge to Facilitate Comparison With Vertebrates. *Front. Physiol.* 9**,** 952. doi: 10.3389/fphys.2018.00952.

Shigeno, S., Kidokoro, H., Tsuchiya, K., Segawa, S., and Yamamoto, M. (2001). Development of the brain in the oegopsid squid, *Todarodes pacificus*: an atlas from hatchling to juvenile. *Zoological Science* 18**,** 1081-1096.

Stephan, H., and Pirlot, P. (1970). Volumetric comparisons of brain structures in bats. *Zeitschrift für zoologische Systematik und Evolutionsforschung* 8**,** 200-236.

Tsuchiya, K. 2000. *Abralia veranyi* (Ruppell 1844). *World Wide Web electronic publication: http://tolweb. org/Abralia_veranyi/19654* [Online].

Uyeno, T.A., and Kier, W.M. (2005). Functional morphology of the cephalopod buccal mass: a novel joint type. *J. Morphol* 264**,** 211-222.

Vijai, D. (2016). Egg Masses of Flying Squids (Cephalopoda: Ommastrephidae). *Journal of Shellfish Research* 35(4)**,** 1007-1012, 1006.

Villanueva, R., Perricone, V., and Fiorito, G. (2017). Cephalopods as predators: a short journey among behavioral flexibilities, adaptions, and feeding habits. *Frontiers in Physiology* 8**,** 598.

Wagstaff, K. (2004). "Clustering with Missing Values: No Imputation Required," in *Classification, Clustering, and Data Mining Applications,* eds. D. Banks, F.R. McMorris, P. Arabie & W. Gaul. (Berlin, Heidelberg: Springer Berlin Heidelberg), p. 649-658.

Wells, M.J. (1978). *Octopus: physiology and behaviour of an advanced invertebrate.* Springer Science & Business Media.

Wirz, K. (1959). Étude biométrique du système nerveux des Céphalopodes. *Bull. Biol. Fr. Belg* 93**,** 78-117.

Wood, J.B., and Day, C.L. 2003. CephBase. The Cephalopod Page. *World Wide Web electronic publication:* *http://www.cephbase.utmb.edu* [Online]. Available: GFLib: FolderLuPhD-C.

Wood, J.B., and Day, C.L. 2006. CephBase. *World Wide Web electronic publication:* *http://www.cephbase.utmb.edu* [Online]. Available: GFLib: FolderLuPhD-C.

Yamamoto, M., Shimazaki, Y., and Shigeno, S. (2003). Atlas of the embryonic brain in the pygmy squid, *Idiosepius paradoxus*. *Zoological Science* 20**,** 163-179.

Yamazaki, A., Yoshida, M., and Uematsu, K. (2002). Post-hatching development of the brain in *Octopus ocellatus*. *Zoological Science* 19**,** 763-771.

York, R.A., Byrne, A., Abdilleh, K., Patil, C., Streelman, T., Finger, T.E., and Fernald, R.D. (2019). Behavioral evolution contributes to hindbrain diversification among Lake Malawi cichlid fish. *Scientific Reports* 9(1)**,** 19994. doi: 10.1038/s41598-019-55894-1.

Young, E.R., Vecchione, M., and Mangold, K. (2019). *Tree of Life Cephalopoda Cuvier 1797. Octopods, squids, nautiluses, etc.* [Online]. http://tolweb.org/Cephalopoda/19386/2019.03.26: The Tree of Life Web Project. [Accessed May, 2020 2020].

Young, J.Z. (1963). The number and sizes of nerve cells in *Octopus*. *Proc. Zool. Soc. Lond* 140**,** 229-254.

Young, J.Z. (1965). The central nervous system of *Nautilus*. *Philosophical Transactions of the Royal Society of London B: Biological Sciences* 249(754)**,** 1-25.

Young, J.Z. (1971). *The anatomy of the nervous system of Octopus vulgaris.* London, UK: Oxford University Press.

Young, J.Z. (1974). The central nervous system of *Loligo* I. The optic lobe. *Philos. Trans. R. Soc. Lond. B* 267**,** 263-302.

Young, J.Z. (1976). The nervous system of *Loligo*. II. Suboesophageal centres. *Philosophical Transactions of the Royal Society B: Biological Sciences* 274(930)**,** 101-167.

Young, J.Z. (1977a). Brain, behaviour and evolution of cephalopods. *Symp. Zool. Soc. Lond.* 38**,** 377-434.

Young, J.Z. (1977b). The nervous system of *Loligo* III. Higher motor centres: the basal supraoesophageal lobes. *Philos. Trans. R. Soc. Lond. B* 276**,** 351-398.

Young, J.Z. (1979). The nervous system of *Loligo*: V. The vertical lobe complex. *Philosophical Transactions of the Royal Society of London B: Biological Sciences* 285(1009)**,** 311-354.

Young, R.E., and Harman, R.F. (1988). "Larva", "paralarva" and "subadult" in cephalopod terminology. *Malacologia* 29**,** 201-207.

Young, R.E., and Mangold, K.M. 1996a. *Pterygioteuthis giardi* Fischer 1896. *World Wide Web electronic publication: http://tolweb. org/Pterygioteuthis_giardi/19751* [Online].

Young, R.E., and Mangold, K.M. 1996b. *Pyroteuthis margaritifera* (Ruppell, 1844). *World Wide Web electronic publication:* *http://tolweb. org/Pyroteuthis_margaritifera/19758* [Online].

Young, R.E., and Mangold, K.M. 1999a. *Egea* Joubin, 1933. *Egea inermis* Joubin, 1933. *World Wide Web electronic publication:* *http://tolweb. org/Egea_inermis/19563* [Online].

Young, R.E., and Mangold, K.M. 1999b. *Galiteuthis glacialis* (Chun 1906). *World Wide Web electronic publication:* *http://tolweb. org/Galiteuthis_glacialis/19572* [Online].

Young, R.E., and Vecchione, M. 2001. *Chtenopteryx sicula* (Verany 1851). *World Wide Web electronic publication:* *http://tolweb. org/Chtenopteryx_sicula/19441* [Online].

Zar, J.H. (1999). *Biostatistical analysis.* Upper Saddle River, N.J.: Prentice Hall.

Zullo, L. (2004). *Functional organisation of the sensory-motor areas in the SNC of Octopus vulgaris.* PhD, Università degli Studi di Napoli Federico II. Facoltà di Scienze Matematiche, Fisiche e Naturali.
